# Expression based biomarkers and models to classify early and late-stage samples of Papillary Thyroid Carcinoma

**DOI:** 10.1371/journal.pone.0231629

**Published:** 2020-04-23

**Authors:** Sherry Bhalla, Harpreet Kaur, Rishemjit Kaur, Suresh Sharma, Gajendra P. S. Raghava

**Affiliations:** 1 Department of Computational Biology, Indraprastha Institute of Information Technology, New Delhi, India; 2 Centre for Systems Biology and Bioinformatics, Panjab University, Chandigarh, India; 3 Bioinformatics Centre, CSIR-Institute of Microbial Technology, Chandigarh, India; 4 CSIR-Central Scientific Instruments Organization, Chandigarh, India; International Centre for Genetic Engineering and Biotechnology, INDIA

## Abstract

**Introduction:**

Recently, the rise in the incidences of thyroid cancer worldwide renders it to be the sixth most common cancer among women. Commonly, Fine Needle Aspiration biopsy predominantly facilitates the diagnosis of the nature of thyroid nodules. However, it is inconsiderable in determining the tumor’s state, i.e., benign or malignant. This study aims to identify the key RNA transcripts that can segregate the early and late-stage samples of Thyroid Carcinoma (THCA) using RNA expression profiles.

**Materials and methods:**

In this study, we used the THCA RNA-Seq dataset of The Cancer Genome Atlas, consisting of 500 cancer and 58 normal (adjacent non-tumorous) samples obtained from the Genomics Data Commons (GDC) data portal. This dataset was dissected to identify key RNA expression features using various feature selection techniques. Subsequently, samples were classified based on selected features employing different machine learning algorithms.

**Results:**

Single gene ranking based on the Area Under the Receiver Operating Characteristics (AUROC) curve identified the *DCN* transcript that can classify the early-stage samples from late-stage samples with 0.66 AUROC. To further improve the performance, we identified a panel of 36 RNA transcripts that achieved F1 score of 0.75 with 0.73 AUROC (95% CI: 0.62–0.84) on the validation dataset. Moreover, prediction models based on 18-features from this panel correctly predicted 75% of the samples of the external validation dataset. In addition, the multiclass model classified normal, early, and late-stage samples with AUROC of 0.95 (95% CI: 0.84–1), 0.76 (95% CI: 0.66–0.85) and 0.72 (95% CI: 0.61–0.83) on the validation dataset. Besides, a five protein-coding transcripts panel was also recognized, which segregated cancer and normal samples in the validation dataset with F1 score of 0.97 and 0.99 AUROC (95% CI: 0.91–1).

**Conclusion:**

We identified 36 important RNA transcripts whose expression segregated early and late-stage samples with reasonable accuracy. The models and dataset used in this study are available from the webserver CancerTSP (http://webs.iiitd.edu.in/raghava/cancertsp/).

## Introduction

The last few decades have witnessed a sharp upsurge in the prevalence of thyroid cancer worldwide, and the incidence rate of thyroid malignancy is still increasing, making it the sixth most common cancer in women as per recent cancer statistics of 2019 [1]. The exposure to radiation and environmental carcinogens are the possible factors implicated for its rise [2]. Histopathologically, there are four types of thyroid cancers, stated as Papillary, Follicular, Medullary, and Anaplastic. Together, Papillary Thyroid Carcinoma (PTC) and Follicular Thyroid Carcinoma (FTC) are known as Differentiated Thyroid Cancer (DTC) and constitute the majority of thyroid malignancy as well as the most common endocrine malignancy [3]. According to the American Cancer Society, the survival rate of early-stage (stage I and stage II) PTC patients is nearly 100%, but the same reduces to 55% in stage 4 [4]. These statistics indicate the need for methods or biomarkers for early detection of thyroid cancer. In this regard, Fine Needle Aspiration (FNA) biopsy of the thyroid nodule, along with subsequent cytological categorization is a reference method. It has been observed that the diagnostic precision of FNA has been subjected to the skill of the operator, intrinsic characteristics of nodules, and cytology interpretation [5]. One of the limitations includes its limited capability to identify follicular lesions [6]. Due to these limitations of FNA cytology, several immunohistochemical markers have been projected, and their efficacy in thyroid cancer diagnosis is still being evaluated. *HBME-1* with *CK19* combination [7] and *LGALS3* [8] have shown promising results as diagnostic biomarkers. Furthermore, the overexpression of *EGFR* has been found to be associated with the severity of the disease [9]. Inferences of these studies point out the biomarker potential of these genetic entities. In spite of accumulating knowledge of genetic alterations accompanying the thyroid cancer incidences in the last 20 years [10], the genomics-based thyroid cancer diagnosis is yet to be realized.

The increasing availability of genomics data has paved the way for a deeper understanding of cancer biology in terms of clinical, diagnostic, and therapeutic capabilities. One such resource is The Cancer Genome Atlas (TCGA), a public endeavor aimed at establishing a comprehensive catalog of genomic alterations occurring in cancers inferred from large-scale genome sequencing of cancerous tissues accompanied by multidimensional analyses [11]. The various types and levels of data like the mRNA expression, genomic mutations, copy number variations, gene fusions, etc. are available for several cancer types.

Analysis of TCGA thyroid cancer samples has described the genomic landscape of thyroid cancer. This study expanded the set of driver genes to incorporate genes like *EIF1AX*, *PPM1D*, and *CHEK2* and several gene fusions. On the basis of multidimensional data analysis, this study proposed the reclassification of thyroid cancers into molecular subtypes that better reflect the underlying molecular signaling pathways, which will further lead to better disease management [12]. Besides, several studies have also tried to reanalyze this data to understand the association of genomic features with survival and progression [13–16]. For instance, Chai et al. have shown that the higher expression of *BRAF* is associated with high tumor aggressiveness regardless of the *BRAF* mutation status. This indicates that both expression and mutation status, are important in determining the prognostic risk [16]. Another study has classified the benign and malignant thyroid tumors using RNA expression of FNA samples with a training dataset of 137 samples and 48 samples of the validation dataset and achieved the specificity of 84% on the validation dataset [17]. In literature, it has been shown that the methylation status of markers like *RASSF1*, *DAPK1*, and *ESR1* has been significantly associated with thyroid cancer subtypes and early detection of thyroid cancer [18]. Furthermore, high expression of *VDR* has been observed to be associated with classic and tall cell subtype, stage IV, and low recurrence-free survival of thyroid cancer [13]. Moreover, previously, it has been shown that transforming growth factor, *CDH1*, *COL1A1*, *CTNNA1*, *ITGA3*, and *FN1* were differentially expressed between benign and malignant nodules of thyroid cancer [19].

Taken together, previous literature mainly focused on the understanding of the association between pathogenesis and progression of the disease with the genomic features and its alterations. Certainly, this helps the researchers and clinicians for a better understanding of the pathogenesis of thyroid cancer. There is still a need for genomic features that are capable of detection of disease at an early stage to improve the outcome of PTC patients. Undoubtedly, it will facilitate the clinicians in choosing the appropriate therapeutic treatment and management of the patients. Although, in the recent past, various genomic features like RNA expression and methylated CpG sites are explored for stage identification of different malignancies [20–22]. To the best of the authors’ knowledge, there is still a lacuna in the understanding between genomic features and stage identification of PTC. Thus, the current study is an attempt for the stage prediction of PTC based on RNA-Seq data of the patients employing machine learning techniques.

In the present study, we have scrutinized the important RNA transcripts that have a reasonable distinguishing capability of segregating early-stage samples from late-stage samples of PTC using various types of bioinformatics analyses. First, we ranked RNA transcripts based on their discriminatory power to classify early and late-stage samples on the basis of the expression threshold. Their gene ontology and pathway analysis were done to ascertain the biological role of key transcripts in transitioning from early to late stage. Next, multiple transcripts were used to develop models that can categorize early and late-stage samples with high precision. Further, the multiclass model was developed to distinguish the normal, early and late-stage samples. Additionally, we have tried to deduce the signature with the minimum number of transcripts capable of distinguishing cancer and normal samples with high accuracy. Eventually, we provide a public domain webserver (CancerTSP) for discrimination of the early and late-stage along with cancerous from the non-cancerous state of the samples based on machine learning models developed in the study.

## Methods

### Datasets

The RNA-Seq data (HTSeq-FPKM, 500 THCA samples, and 58 normal or adjacent non-tumorous samples obtained from 500 PTC patients) was retrieved from the Genomic Data Commons (GDC) data portal (https://portal.gdc.cancer.gov/). Notably, there were adjacent non-tumorous or normal samples available for 58 patients out of 500 patients only. In addition, manifest, metadata, clinical data, biospecimen files were also downloaded from the GDC data portal to obtain clinical information of the patients using Biospecimen Core Resource (BCR) IDs of patients. For every sample, mRNA expression of 60,483 RNA transcripts was reported in terms of FPKM (Fragments Per Kilobase Million) values. To ascertain the importance of the different types of transcripts, we segregated transcripts into subtypes like protein-coding, LincRNA, snoRNA, snRNA, and miRNA, etc. transcripts using annotation from GENCODE v22 (S1 Table).

### Datasets for prediction models

#### Training and validation datasets

Of the total 500 THCA samples, 281 were of stage 1, 52 of stage 2, 112 of stage 3, and 55 samples were of stage 4. As in stage 1 and stage 2, the tumor is still confined to the thyroid and has not spread to the central compartment of lymph nodes; therefore, we combined stage 1 and stage 2 samples as early-stage samples [23]. In stage 3 and stage 4, cancer spreads to lymph nodes including other organs; therefore, we combined stage 3 and stage 4 samples into late-stage samples. Thus, our stage classification dataset contains 333 early and 167 late-stage samples. This approach has been previously implemented in various similar types of studies [20–22]. We divided this dataset into training and validation dataset with 80:20 ratio, which was already applied in different studies to develop stage prediction tools for renal cancer and liver cancer, i.e., CancerCSP and CancerLSP, and *in silico* tools to predict viral siRNA efficacy and anti-fungal peptides, i.e., VIRsiRNApred and Antifp [21, 22, 24, 25]. The 80% of stage 1 and 2 samples were labeled as the early training set, while, rest of 20% samples from stage 1 and 2 were used as a validation dataset for early-stage samples. Similarly, training and validation datasets for late-stage samples were created. We used, training dataset for selecting the features and selecting the best parameters for various machine learning algorithms using grid search. Finally, the models were developed on the training dataset using best-obtained parameters and were validated on the validation dataset. The clinical features of the patients are shown in S1 Fig.

In addition to stage classification, prediction models were also developed for discrimination of cancer and normal tissue samples. Towards this, a dataset comprised of 500 cancer samples and 58 normal or adjacent non-tumorous samples was used. Additionally, multiclass classification prediction models were developed for the categorization of normal, early, and late-stage samples. The dataset used for multiclass classification comprised of 58 normal, 333 early-stage, and 167 late-stage samples. These datasets were further subdivided into training and validation datasets in a ratio of 80:20, similar to stage classification models.

#### External validation dataset

To assess the classification performance of the top performing set of features or RNA transcripts, eventually, the performance was evaluated on the external validation dataset with accession GSE48953, obtained from Gene Expression Omnibus (GEO) database [26]. GSE48953 data is based on high throughput sequencing (RNA-Seq) [27] and consists of expression profiling of 20 PTC patients including 17 early-stage patients (stage 1) and 3 late-stage patients (stage 3). To validate the models on the external validation dataset, first, the TCGA dataset was log2 transformed. Subsequently, the GSE48953 expression data was quantile normalized by using the TCGA training dataset as reference (target set) employing the *FSQN* R package [28].

### Data processing

The contribution of the batch effect in the TCGA-THCA expression data was checked using TCGA Batch Effects Viewer [29]. Further, the FPKM values had a wide range of variation; the values were log2 transformed after the addition of 1.0 as a constant number to each of the values. Adding the constant one ensured that all of the transformed values would be positive. This approach is common in the literature related to the analysis of RNA expression [20, 30]. Thereafter, features with low variance (less than 0.25) were removed by employing *caret* package in R, followed by the Z-score normalization of data. The Eqs (1) and (2) were used for log transformation and normalization of data, respectively.

x=log2(FPKM+1) (1)

$$Z-score=\frac{x-\mu }{\sigma }$$ (2)

In Eq (2), *Z-score* is the normalized scaled and centered score, *x* is the log-transformed transcript expression, μ is the mean of transcript’s expression in the training dataset, and σ is the standard deviation of a transcript in the training dataset. The log2 transformed validation data was *Z-score* normalized by taking the mean and standard deviation of training features.

### Features filtering using threshold-based models

In the current study, we have considered the expression values of RNA transcripts as features for the analysis. In order to identify the classification potential of each RNA transcript, we employed a simple expression threshold-based approach similar to our previous study [21]. Briefly, in this approach, for every transcript, we designated a threshold, which determines whether a sample is in the early or late stage of cancer. The threshold was selected by iterating from the minimum to maximum expression of that transcript across all the patients. The threshold which gives maximum AUROC of classification between early and late-stage sample was reported. Briefly, if the mean expression of a transcript is greater in the early-stage than late-stage, and the log2 FPKM value of that transcript is found to be higher than the selected threshold for a given sample, then we assign that sample as early-stage otherwise late-stage. While, if the transcript’s average log2 FPKM value is greater in late-stage as compared to the early-stage, and the log2 FPKM of that transcript is greater than the threshold for a given sample, then we assign that sample as the late-stage sample otherwise as to the early-stage. Subsequently, threshold-based models were developed for each feature. Eventually, they were ranked based on their performance in the segregation of samples into different classes. Using the method mentioned above, AUROC was also calculated for cancer vs. normal samples.

### Feature selection

To further improve the classification accuracy and to develop multiple-genes classification models, we used state-of-the-art techniques to select relevant features. First, we performed feature selection by employing an attribute evaluator named ‘SymmetricalUncertAttributeSetEval’ with the search method of ‘FCBFSearch’ of WEKA. The FCBF (Fast Correlation-Based Feature) algorithm uses mainly correlation to identify important discriminating features in high-dimensional datasets in reduced feature space [31]. Secondly, we employed the sklearn.feature_selection. F_ANOVA method of feature selection using the Scikit-learn package [32]. This method selects the features by computing F-statistics.

Third, we applied two more advanced feature selection methods for the features (both protein-coding and non-coding transcripts together), which performed best in comparison to other features. One was the (Support Vector Classifier) SVC with the L1 penalty using Scikit-learn [33], and the other was a Wrapper approach for feature selection. In a wrapper-based approach, human opinion dynamics optimizer has been used as a search algorithm to search through the space of possible feature subsets with the objective of maximizing MCC on the training set. This is an iterative algorithm in which each candidate solution represents a feature subset. The solution is encoded using a 100-bit binary vector where 1 and 0 indicate the presence and absence of a feature in a subset, respectively. The quality of features selected is evaluated using support vector machine (SVM) and 10-fold cross-validation. The details of the algorithm can be found in publications [34, 35]. The algorithm has been implemented in MATLAB® using LIBSVM and CODO (an open-source library hosted on https://github.com/rishemjit/CODO).

### Implementation of machine learning techniques

We have developed machine learning models using two software; Scikit-learn package and Waikato Environment for Knowledge Analysis (WEKA) [36]. We employed SVC using Scikit-learn and used the Radial Basis Function (RBF) kernel of SVC at different parameters; g ∈ [10^−3^–10], c ∈ [1–10] using grid search for optimizing the SVC performance. In addition, random forests, sequential minimal optimization (SMO), Naïve Bayes, and J48 were employed using WEKA software.

### Cross-validation technique

The validation is an indispensable part of evaluating the performance of a prediction method. In this direction, the ten-fold cross-validation technique is exploited to calculate the performance of early vs. late-stage and cancer vs. normal classification models. Here, the dataset is randomly divided into ten sets, from which nine sets are used as training sets and the leftover tenth set as a testing dataset. This process is repeated ten times in such a manner that each set is used once as a testing dataset.

### Performance measures

In the present study, the performance of different models was measured by employing threshold-dependent and threshold-independent parameters. In case of threshold-dependent parameters, sensitivity (Sens), specificity (Spec), overall accuracy (Acc (%)), Matthews correlation coefficient (MCC), Precision, Recall and F1 score were calculated by using Eqs (3)–(9), respectively:

$$Sensitivity\left(Sens\right)=\frac{TP}{TP+FN}*100$$ (3)

$$Specificity\left(Spec\right)=\frac{TN}{TN+FP}*100$$ (4)

$$Accuracy\left(Acc\right)=\frac{TP+TN}{TP+FP+TN+FN}*100$$ (5)

$$MCC=\frac{\left(TP*TN\right)\left(FP*FN\right)}{\sqrt{\left(TP+FN\right)\left(TN+FP\right)\left(TN+FN\right)}}$$ (6)

$$Precision=\frac{TP}{TP+FP}$$ (7)

$$Recall=\frac{TP}{TP+FN}$$ (8)

$$F1\;score=2*\frac{Precision*Recall}{Precision+Recall}$$ (9)

where FP, FN, TP, and TN are false positive predictions, false negative predictions, true positive and true negative, respectively.

We also calculated a threshold-independent parameter called AUROC value, which is computed from the receiver operating characteristic (ROC) plot in this study. The ROC curve is produced by plotting true positive rate against the false positive rate at different thresholds. In addition, to ascertain the reliability of prediction, we also calculated PPV (Positive Predictive Value) and NPV (Negative Predictive Value) at various thresholds using the probability score obtained by employing SVC.

### Multiclass classification

The multiclass classification was implemented using the approach of one vs. rest multiclass SVC classifier employing the Scikit-learn package [37].

### Functional enrichment of genes

Enrichment of genes was done using the Enrichr tool [38, 39]. Only those terms were selected for which adjusted p-value was less than 0.05. The Enrichr tool applies the Fisher’s exact test along with the adjustment using Bonferroni correction to give adjusted p-values.

## Results

**Fig 1:**
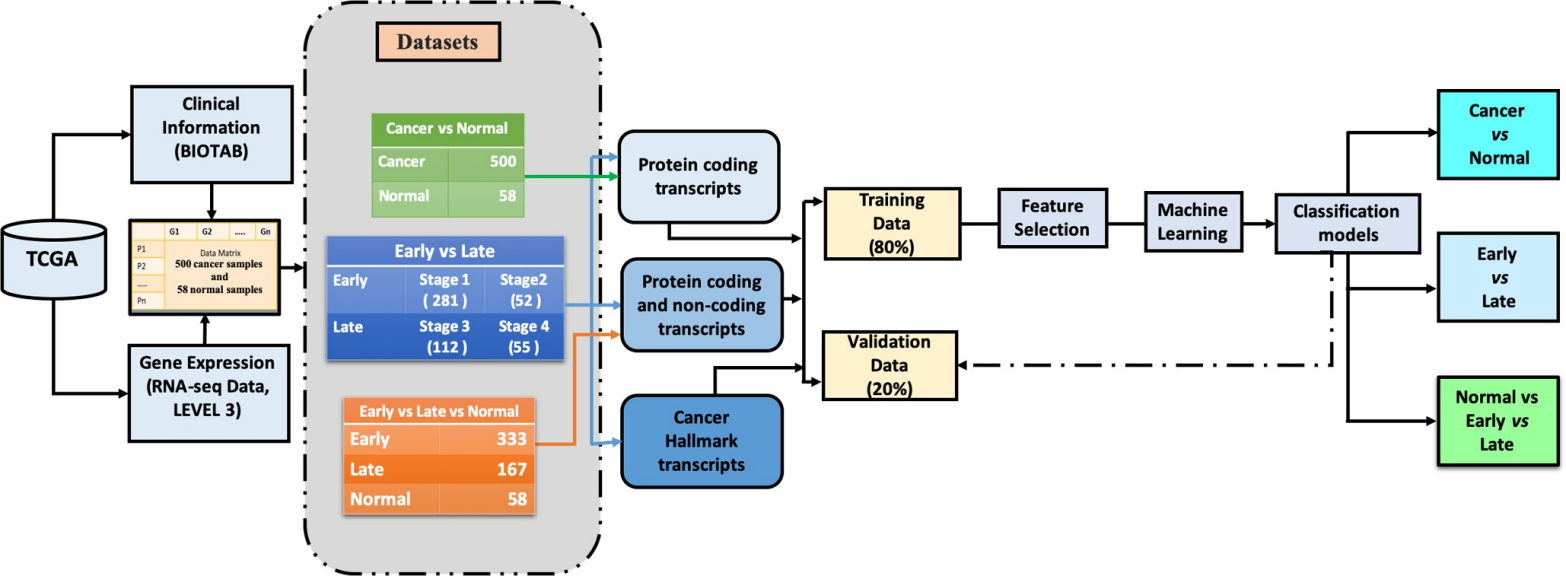
The overall flow of the study, including the number of samples in each class and types of feature selections explored for the development of machine learning models. The green arrows represent that only protein-coding features have been explored to segregate cancer and normal samples. The blue arrows indicate that both protein-coding and non-protein coding features individually and in combination have been explored to segregate early vs late-stage samples. For multiclass classification, protein-coding and non-protein coding transcripts in combination are explored to segregate early, late and normal samples.

The primary objective of the current study is the identification of the key genomic entities from 60,483 RNA transcripts that can segregate early vs. late-stage samples and tumor vs. non-tumor samples of PTC based on extensive bioinformatics analysis. Subsequently, *in silico* prediction models were developed using key RNA transcripts implementing various machine learning techniques. Notably, in the current study, we have considered the expression values of RNA transcripts as features for the analysis. To rule out the batch effects in the subsequent analysis, we calculated the Dispersion Separability Criterion (DSC) value. The DSC value for the TCGA-THCA data is 0.26 (p-value <0.0005), which is less than 0.5, a commonly used DSC threshold for indicating batch effects (S2 Fig). This indicates that there were no strong batch effects in the TCGA-THCA expression data. The overall workflow of this study is presented in Fig 1, and the results are explained in the following sections.

### Single RNA transcript-based stage classification

To rank the classification potential of each RNA transcript for segregating early and late-stage samples, we developed a stage classification method using the expression threshold of each RNA transcript (see methods). Briefly, in this approach, for every transcript/feature from 60,483 RNA transcripts, we designated a threshold, which determines whether a sample is in the early or late stage of cancer. This threshold was chosen by iterating from the minimum to maximum value of the expression for that transcript across all the patients. Here, a stage was assigned to a sample if the expression of an RNA transcript was more than the threshold; in case the RNA transcript was overexpressed in samples of that stage. Subsequently, RNA transcripts were ranked based on the discriminatory power in terms of AUROC of the threshold model. Eventually, we obtained 179 transcripts which had an AUROC score greater than equal to 0.60 and named as THCA-EL-AUROC. We have selected a cut off value of 0.60 to select the maximum features which performed better than random features (AUROC = 0.50). The THCA-EL-AUROC panel contains key transcripts that can help to discriminate early-stage samples from late-stage samples. Thus, they can be further explored for their potential biomarker capability in stage identification of PTC samples (S2 Table and S3 Fig), although more research is needed to confirm this hypothesis. The *DCN* protein (overexpressed in late-stage) coding transcript shows the highest AUROC of 0.66. It is a proteoglycan whose role is well established in discriminating benign and metastatic thyroid and other tumors [40, 41]. Out of 179 transcripts, 166 are protein-coding, six are lincRNA, and the other seven belong to other classes of non-coding transcripts (S2 Table). The 34 out of 179 RNA transcripts have a significant adjusted p-value of less than 0.05 (S2 Table).

The 166 protein-coding transcripts are significantly enriched in nine oncogenic signatures from MSigDB Database [42] (S3 Table) that points out these genes have also been previously implicated in many cancers. Additionally, the 166 protein-coding transcripts are enriched in many pathways of the KEGG database, such as Focal adhesion pathway (5% genes, adjusted p-value = 4.0e-5), PI3K-Akt signaling pathway (3.5% genes, adjusted p-value = 0.001), Proteoglycans in cancer (3% genes, adjusted p-value = 0.007). Further, the enriched terms of gene ontology for 166 protein-coding transcripts are mainly related to matrix organization and collagen binding (S4 Fig).

### Stage classification model using multiple RNA transcripts

As shown in the above section and S2 Table, individual 179 RNA transcripts (THCA-EL-AUROC) have limited ability to classify early and late-stage samples with maximum AUROC 0.66. Therefore, to develop a model that can classify the stage of samples with high precision, we used the expression of multiple RNA transcripts. The stage classification models based on THCA-EL-AUROC (179 RNA transcripts) features were developed implementing a number of machine learning techniques. As shown in S4 Table, the SVC model achieved F1 score of 0.69 with AUROC of 0.72 (95% CI: 0.67–0.78) on training data and F1 score of 0.67 on the validation data with AUROC of 0.70 (95% CI: 0.59–0.82). There is a marginal improvement in the performance of the models developed using multiple transcripts (AUROC 0.70, S4 Table) as compared to the single gene model (AUROC 0.66 for DCN gene). We further tried few other intricate feature selection methods taking different subsets of transcripts in the following sections to further improve the performance of our models.

### Protein-coding transcripts

From the previous results, it is evident that protein-coding transcripts were the major type of transcripts in THCA-EL-AUROC signature, therefore in this analysis, at first, we selected 19,814 protein-coding transcripts from 60,483 transcripts. Subsequently, different feature selection techniques like FCBF-WEKA (Fast correlation-based feature selection method present in WEKA) [43] and F_ANOVA [32] applied on these protein-coding transcripts. Thereafter, the prediction models were developed based on the selected set of features employing different machine learning techniques like SVC, RF, SMO, Naïve Bayes, and J48. The SVC model based on 37 features (selected by FCBF-WEKA) is the top performer with F1 score of 0.75 and 0.79 AUROC (95% CI: 0.74–0.84) on training data and F1 score of 0.72 with 0.66 AUROC (95% CI: 0.54–0.77) on the validation (THCA-EL-PC, Table 1) using 37 features obtained using SVC. There was a marginal increase in the performance in terms of accuracy, but the number of features was reduced to a reasonable extent as compared to THCA-EL-AUROC. The performance using other algorithms like Random Forest, Naïve Bayes, SMO, and J48 was lower on the validation dataset, as shown in Table 1.

**Table 1:** The performance measures of the prediction models developed based on 37-protein-coding mRNA feature set (THCA-EL-PC) selected by FCBF-WEKA feature selection method on training and validation dataset by implementing various machine-learning algorithms.

Classifier	Dataset	TP	FP	TN	FN	Recall (%)	Precision (%)	Spec (%)	Acc (%)	MCC	AUROC (95% CI)	F1 Score
**SVC**	Training	219	52	81	46	82.64	0.81	60.9	75.38	0.44	0.79 (0.74–0.84)	0.75
	Validation	57	18	16	11	83.82	0.76	47.06	71.57	0.33	0.66 (0.54–0.77)	0.72
**SMO**	Training	244	68	65	21	92.08	0.78	48.87	77.64	0.47	0.70 (0.66–0.75)	0.78
	Validation	60	24	10	8	88.24	0.71	29.41	68.63	0.22	0.59 (0.50–67)	0.69
**J48**	Training	180	47	86	85	67.92	0.79	64.66	66.83	0.31	0.66 (0.61–0.72)	0.66
	Validation	50	16	18	18	73.53	0.76	52.94	66.67	0.26	0.66 (0.55–0.77)	0.67
**NB**	Training	190	39	94	75	71.7	0.83	70.68	71.36	0.4	0.77 (0.72–0.82)	0.71
	Validation	47	15	19	21	69.12	0.76	55.88	64.71	0.24	0.63 (0.51–0.75)	0.46
**RF**	Training	225	52	81	40	84.91	0.81	60.9	76.88	0.47	0.8 (0.75–0.85)	0.76
	Validation	52	18	16	16	76.47	0.74	47.06	66.67	0.24	0.60 (0.47–0.73)	0.5

TP: True Positive; FP: False Positive; TN: True Negative; FN: False Negative; Spec: Specificity; Acc: Accuracy; MCC: Matthews Correlation Coefficient; AUROC: Area under Receiver operating characteristic curve; CI: Confidence Interval.

To understand the interaction network among the key identified protein-coding genes, interaction analysis was performed in the STRING database [44] (S5 Fig) with THCA-EL-PC transcripts. On adding less than ten indirect nodes, we observed three important clusters enriched in different pathways. *HIST1H2BJ*, the transcript present in our signature forms a cluster, and this cluster is enriched in nucleosome cellular component. This cluster has also shown to be related to the progression of prostate cancer [45]. Another cluster of three genes is enriched in the dihydrolipoyl dehydrogenase complex (FDR <0.01), out of which *DBT* is present in our original signature. In addition, one more cluster of three genes is a part of the checkpoint clamp complex, out of which *RAD1* is present in the original signature, and is involved in DNA damage response [46].

In addition, the top 100 features (S5 Table) were selected using the F_ANOVA feature selection method. The SVC based model has achieved F1 score of 0.71 with AUROC of 0.73 (95% CI: 0.68–0.79) on the training data, and F1 score of 0.68 with 0.71 AUROC (95% CI: 0.60–0.82) is obtained on the validation data (THCA-EL-F-PC, S6 Table).

### Cancer hallmark based transcripts

Hanahan and Weinberg uncovered the importance of eight biological processes that played a vital role in tumor growth and metastatic propagation and called them as cancer hallmark processes [47]. Thus, genes involved in these processes could also act as key signature markers. Here, we have tried to ascertain only relevant transcripts from this subset of cancer hallmark genes.

To develop prediction models based on the cancer hallmark genes, initially, 4,814 cancer hallmark specific genes extracted from 60,483 transcripts. Subsequently, the number of features was reduced using various features selection techniques followed by the development of prediction models. Towards this, the 15 transcripts selected by FCBF-WEKA (THCA-EL-H) from cancer hallmark genes were used to develop models. The F1 score of 0.68 with AUROC of 0.71 (95% CI: 0.66–0.77) is attained on the training data, while the F1 score of 0.69 with AUROC of 0.73 (95% CI: 0.61–0.85) is obtained on the validation dataset (S7 Table). Out of 15 transcripts, two transcripts *PROC* and *NLK* (adjusted p-value = 0.002) are involved in the developmental pathway of the Wnt signaling, and are shown to be dysregulated in cancer [48]. The other two genes *CYSLTR1* and *ADRB1*, are enriched in GPCRs terms (adjusted p-value = 0.04). *CYSLTR1* is upregulated in colon cancer patients and associated with poor prognosis [49]. A similar performance is obtained on 50 genes selected using the F_ANOVA method (THCA-EL-FH, S8 Table).

### Protein-coding and non-coding transcripts

Further to ascertain the role of both coding and non-coding transcripts, we explored all the 60,483 transcripts to identify relevant features that can segregate early and late stage samples. The 78 transcripts (THCA-EL-All-WEKA) were chosen using FCBF-WEKA based feature selection algorithm. The SVC model based on the THCA-EL-All-WEKA panel performed well and attained F1 score of 0.78 and 0.86 AUROC (95% CI: 0.83–0.90) on the training dataset and F1 score of 0.70 with 0.73 AUROC (95% CI: 0.63–0.84) on the validation dataset (S9 Table). Among the 78 selected features, 28 are protein-coding transcripts, 12 are long non-coding RNA, 12 are antisense transcripts, 11 are processed pseudogenes, and others are different non-coding RNAs (S10 Table).

Nest we applied another feature selection method called F_ANOVA to select top 100 features. The SVC model using these 100 features achieved F1 score of 0.72 with 0.77 AUROC (95% CI: 0.66–0.77) on the training data and F1 score of 0.63 with 0.68 AUROC (95% CI: 0.56–0.79) on the validation data (THCA-EL-All-F, S12 Table).

Additionally, the top 100 features selected using the F_ANOVA were further subjected to the second stage of feature selection. In this stage, a wrapper-based approach combining human opinion dynamics optimizer and SVC has been employed (see methods for details). The number of features was reduced to 27 (S13 Table, THCA-EL-CODO). It achieved F1 score of 0.59 and 0.72 AUROC (95% CI: 0.67–0.78) on the training set and F1 score of 0.58 and 0.73 AUROC (95% CI: 0.62–0.84) on the validation set using the Naïve Bayes Classifier (S14 Table).

From the above analysis, it has been observed that the prediction models based on both protein-coding and non-coding transcripts gave higher performance as compared to protein-coding and cancer hallmark protein-coding transcripts alone. One of our recent studies has shown that the prediction model based on the SVC-L1 feature selection method achieved higher performance with the minimum number of features [50]. Hence, we performed feature selection using the SVC with L1 penalty (see methods). SVC-L1 method resulted in 36 transcripts (shown in S15 Table). The SVC classifier based on the THCA-EL-SVC-L1 features attained F1 score of 0.75 with 0.73 AUROC (95% CI: 0.62–0.84) (Table 2) on the validation data. Notably, the prediction model based on 36 features is the best model among all the prediction models developed using different feature sets in classifying early and late-stage samples in terms of the number of features, accuracy, and F1 score on the validation dataset.

**Table 2:** The performance measures of the prediction models developed based 36-full feature set (THCA-EL-SVC-L1) selected by SVC-L1 on training and validation dataset by implementing various machine-learning algorithms.

Classifier	Dataset	TP	FP	TN	FN	Recall (%)	Precision	Spec (%)	Acc (%)	MCC	AUROC (95% CI)	F1 Score
**SVC**	Training	228	17	116	37	86.04	0.93	87.22	86.43	0.71	0.93 (0.91–0.96)	0.86
	Validation	52	10	24	16	76.47	0.84	70.59	74.51	0.45	0.73 (0.62–0.84)	0.75
**SMO**	Training	252	30	103	13	95.09	0.89	77.44	89.2	0.75	0.86 (0.82–90)	0.89
	Validation	59	16	18	9	86.76	0.79	52.94	75.49	0.42	0.7 (0.60–0.79))	0.75
**J48**	Training	178	52	81	87	67.17	0.77	60.9	65.08	0.27	0.66 (0.60–0.71)	0.65
	Validation	51	17	17	17	75	0.75	50	66.67	0.25	0.62 (0.50–0.73)	0.67
**NB**	Training	239	39	94	26	90.19	0.86	70.68	83.67	0.63	0.87 (0.83–0.91)	0.84
	Validation	58	15	19	10	85.29	0.79	55.88	75.49	0.43	0.72 (0.62–0.83)	0.75
**RF**	Training	197	32	101	68	74.34	0.86	75.94	74.87	0.48	0.84 (0.80–0.88)	0.73
	Validation	46	11	23	22	67.65	0.81	67.65	67.65	0.34	0.75 (0.64–0.85)	0.69

TP: True Positive; FP: False Positive; TN: True Negative; FN: False Negative; Spec: Specificity; Acc: Accuracy; MCC: Matthews Correlation Coefficient; AUROC: Area under Receiver operating characteristic curve; CI: Confidence Interval.

Further, we also calculated PPV and NPV on various thresholds of the SVC probability score (Table 3). On the training data, for the SVC score greater than 0.90, 161 early-stage samples are correctly predicted out of a total of 170 samples predicted as early-stage samples (PPV = 94.71%). In the case of late-stage samples, 60 out of 64 late-stage predicted samples are correct (NPV = 93.75%). In the case of validation data, the PPV is 85.71%, and the NPV is 66.67% (Table 3). This shows that at the SVC score of 0.90, there is a high probability of correct positive (early-stage) and negative (late-stage) prediction. At the threshold of 0.70, at which we presented other performance measures in Table 2, the PPV for training data is 93.03%, and NPV is 94.51%, while, in case of validation, the PPV is 83.87%, and NPV is 63.64% (Table 3).

**Table 3:** The performance of SVC based model at the different threshold in term of the probability of correct prediction, developed using 36-full feature set (THCA-EL-SVC-L1) on training and validation dataset.

Threshold/ Cut-offs	Prediction of Early-stage			Prediction of Late-stage		
	Total Predictions	Correct Prediction	PPV	Total Predictions	Correct Prediction	NPV
**Performance of Training Dataset**						
1.00	16	16	100.00	6	6	100.00
0.95	136	131	96.32	42	40	95.24
0.90	170	161	94.71	64	60	93.75
0.85	199	188	94.47	71	67	94.37
0.80	219	207	94.52	80	76	95.00
0.75	233	221	94.85	86	81	94.19
0.70	244	227	93.03	91	86	94.51
0.65	254	235	92.52	98	90	91.84
0.60	261	240	91.95	106	97	91.51
**Performance of Validation Dataset**						
1.00	0	0	0.00	0	0	0.00
0.95	28	25	89.29	9	7	77.78
0.90	42	36	85.71	12	8	66.67
0.85	47	39	82.98	15	9	60.00
0.80	54	45	83.33	16	10	62.50
0.75	58	48	82.76	19	12	63.16
0.70	62	52	83.87	22	14	63.64
0.65	65	53	81.54	22	14	63.64
0.60	70	56	80.00	25	16	64.00

PPV: Positive Predictive Value; NPV: Negative Predictive Value.

One of the advantages of this prediction model is that it resulted in a more balanced sensitivity and specificity along with higher F1 score on a smaller number of features (36 features, THCA-EL-SVC-L1) as compared to 78 features (THCA-EL-All-WEKA) selected by WEKA. These 36 transcripts consist of 17 protein-coding genes, six long non-coding RNAs, and rest other types of non-coding RNA transcripts (S15 Table). The *TERT* gene in this signature has been an important oncogene in the case of PTC [51]. Overexpression of *TERT* induced by the MAP pathway has shown to aggravate tumor development [52].

This model is deemed as the paramount model in our analysis for binary classification for early and late-stage samples. This also points out that both protein-coding and non-coding transcripts play an important role in tumor development.

#### Independent validation

Eventually, to assess the classification potential of these features, validation performance is also evaluated on the external validation dataset, i.e., GSE48953, in addition to the independent dataset from TCGA. The GSE48953 dataset contains only 18 common features with that of 36-features signature. Therefore, we developed a model based on only those common 18 features. This model correctly predicted 70.6% (12 samples out of 17 samples) of early samples and 100% (3 samples) of late-stage samples of external validation dataset, as shown in Table 4. These results further strengthen and validate the classification potential of our signature for the segregation of early and late-stage samples.

**Table 4:** The performance measures of the SVC based model, developed using 18-features set on training (TCGA dataset) and external validation (GSE48953) dataset.

Dataset	TP	FP	TN	FN	Recall (%)	Precision	Spec (%)	Acc (%)	MCC	AUROC (95% CI)	F1 Score
**Training**	220	50	83	45	83.02	0.81	62.41	76.13	0.46	0.81 (0.76–0.85)	0.76
**ExternalValidation**	12	0	3	5	70.59	1.00	100	75	0.51	0.78 (0.58–9.98)	0.75

TP: True Positive; FP: False Positive; TN: True Negative; FN: False Negative; Sens: Sensitivity; Spec: Specificity; Acc: Accuracy; MCC: Matthews Correlation Coefficient; AUROC: Area under Receiver operating characteristic curve; CI: Confidence Interval.

**Fig 2:**
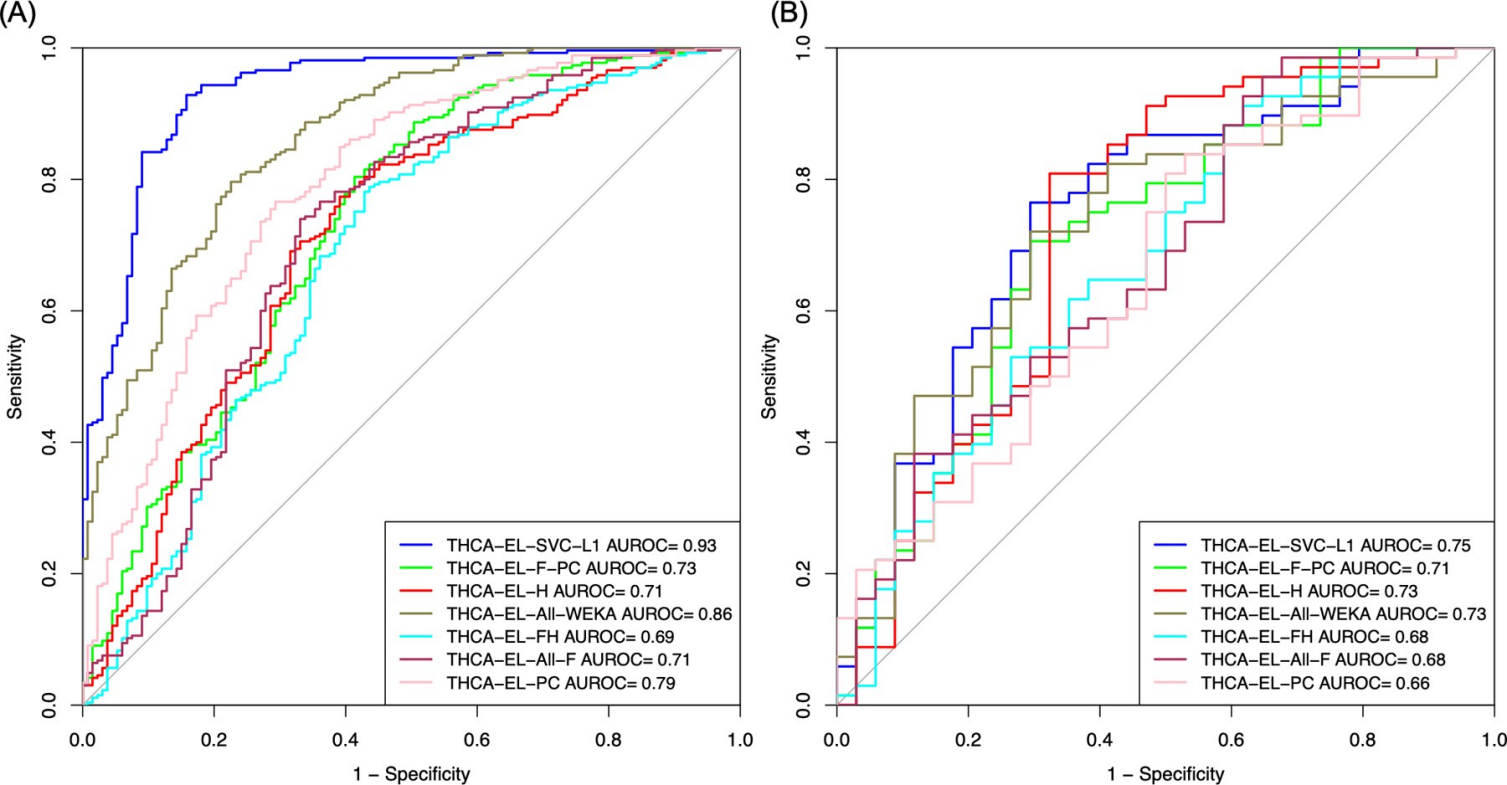
The AUROC plot comparing the performance of prediction models based on different feature sets for segregating early and late-stage tissue samples of (A) Training dataset and (B) Validation dataset.

The performance of different prediction models based on various feature sets for the segregation of early-stage and late-stage samples of training and validation datasets in terms of AUROC curves is depicted in Fig 2.

### Multiclass classification

One of the limitations of binary classification is that it would force even normal samples into either early or late-stage samples. Therefore, we implemented the multiclass classification by considering the normal samples available in TCGA for thyroid cancer.

From the above analysis, it has been observed that the prediction model developed based on features selected by the SVC-L1 method has higher performance with the minimum number of features. Therefore, we employed the SVC-L1 to select 107 RNA-transcripts (THCA-NEL-M, S16 Table) from 60,483 transcripts and classified normal, early and late-stage samples. This model obtained F1 score of 0.99, 0.88, 0.77 (Normal, Early, and Late) on the training dataset with AUROC of 0.99 (95% CI: 0.98–0.99), 0.93 (95% CI: 0.92–0.94), and 0.91 (95% CI: 0.90–0.93), respectively. The same model on the validation data attained F1 score of 0.88, 0.78 and 0.55 (Normal, Early and Late) with AUROC of 0.95 (95% CI: 0.84–1), 0.76 (95% CI: 0.66–0.85), and 0.72 (95% CI: 0.61–0.83), respectively (Table 5).

**Table 5:** The performance measures of the multiclass prediction model developed based on 107 features selected by SVC-L1 on training and validation dataset by implementing SVC.

	Training Data							
Class	Recall (%)	Precision (%)	Spec (%)	Acc (%)	MCC	AUROC (95% CI)	F1 Score	Number of samples
**Normal**	100	98	99	99	0.98	0.99(0.98–0.99)	0.99	46
**Early**	88	86	79	86	0.72	0.93(0.92–0.94)	0.88	265
**Late**	77	81	92	87	0.67	0.91(0.90–0.93)	0.77	133
	**Validation Data**							
**Normal**	92	92	99	98	0.91	0.95 (0.84–1.00)	0.88	12
**Early**	79	75	58	74	0.44	0.76 (0.66–0.85)	0.78	68
**Late**	53	58	87	75	0.34	0.72 (0.61–0.83)	0.55	34

Spec: Specificity; Acc: Accuracy; MCC: Matthews Correlation Coefficient; AUROC: Area under Receiver operating characteristic curve.

### Discrimination of cancer vs. normal samples

#### Single gene-based models

In this section, we ranked RNA transcripts on this basis of their AUROC for categorizing the PTCs samples from the non-tumorous samples. First, out of 60,483 features, those features with a variance of less than 0.02 were removed; thus, the number of transcripts reduced to 24,334. Among 24,334 transcripts, there are 8,180 RNA transcripts, which have AUROC of 0.6 or higher. Further, to identify highly discriminatory RNA transcripts, we selected 426 RNA transcripts having AUROC of 0.85 or greater (S17 Table). The overlapping sense transcript *RP11-363E7*.*4* and protein-coding transcript *FAM84A* show AUROC of 0.96 and 0.95, respectively, for classifying cancer and normal samples. Gene Ontology pathway enrichment analysis for 386 protein-coding transcripts from the top 426 transcripts is shown in S6 Fig. Gene enrichment analysis revealed that 11 genes from the 386 transcripts are involved in the axon guidance pathway from KEGG. These genes comprising axon guidance pathway have been shown to play an important role in tumorigenesis [53]. Enrichment analysis shows that eight genes from 386 signatures have been also found in lung cancer-specific 86 genes defined in KEGG. Further, many of the genes are enriched in biological processes that regulate the expression of non-coding RNAs. The enriched cellular components’ terms are mostly related to interleukin receptor complexes, T-cell receptor complexes, and plasma membrane components. Serum IL-2 has been shown to discriminate patients with active thyroid cancer from the healthy with a sensitivity of 98%, and specificity of 58% [54]. Besides the protein-coding genes, 17 long non-coding RNAs were identified with AUROC above 0.85.

Among 17 lincRNAs, the *PTCSC3* has been reported as highly thyroid-specific, and found to be downregulated in thyroid tumor tissues and thyroid cell lines [55]. *LINC00936*, *RP11-774O3*.*3* and *LINC00205* have been observed to be involved in other cancers [56, 57]. Taken together, it points out the literature validation of the key signatures identified in our study.

*Protein-coding RNA transcript-based signatures*. Our next goal was to develop a prediction model based on the least number of only protein-coding RNA features to classify cancer and normal samples with high precision. Therefore, we selected the top five features *RELN*, *RASSF9*, *PLA2R1*, *MMRN1*, and *RPS6KA5* using F_ANOVA (THCA-CN-F). The prediction models were developed based on these five RNA-transcripts using various machine-learning algorithms. The SVC model attained F1 score of 0.98 and 0.97 AUROC (95% CI: 0.93–1) on the training dataset and F1 score of 0.97 and 0.99 AUROC (95% CI: 0.91–1) on the validation dataset (Table 6). As there was a large difference in the number of cancer samples and normal samples, we down-sampled the larger dataset and selected only 58 cancer samples corresponding to the 58 normal samples (THCA-CN-P). Subsequently, leave-one-out SVC model was developed using the same five features and obtained 0.99 AUROC. Also, we selected random 58 cancer samples (THCA-CN-R) and developed a leave-one-out cross-validation SVC model and obtained 0.96 AUROC (S7 Fig). As there is no outsized change in the performance by down-sampling the cancer samples, we incorporated the model with all the 500 samples as the final model.

**Table 6:** The performance measures of prediction models developed based on 5-protein coding transcripts (THCA-CN-F) feature set selected by F_ANOVA feature selection method for discriminating cancer and normal patients on training and independent validation dataset.

Classifier	Dataset	TP	FP	TN	FN	Recall (%)	Precision (%)	Spec (%)	Acc (%)	MCC	AUROC (95% CI)	F1 Score
**SVC**	Training	396	4	42	4	99.00	0.99	91.3	98.21	0.9	0.97 (0.93–1)	0.98
	Independent Validation	97	0	12	3	97.00	1.00	100.00	97.32	0.88	0.99 (0.91–1)	0.97
**SMO**	Training	396	6	40	4	99.00	0.99	86.96	97.76	0.88	0.93 (0.88–0.98)	0.98
	Validation	97	0	123	97	97.00	1.00	100.00	97.32	0.88	0.98 (0.96–1)	0.87
**J48**	Training	397	9	37	3	99.25	0.98	80.43	97.31	0.85	0.85 (0.76–95)	0.97
	Validation	97	3	9	3	97.00	0.97	75.00	94.64	0.72	0.87 (0.74–0.99)	0.87
**NB**	Training	383	3	43	17	95.75	0.99	93.48	95.52	0.80	0.95 (0.91–0.99)	0.96
	Validation	91	0	12	9	91.00	1.00	100.00	91.96	0.72	0.96 (0.93–0.99)	0.92
**RF**	Training	393	5	41	7	98.25	0.99	89.13	97.31	0.86	0.97 (0.93–1)	0.97
	Validation	95	0	12	5	95.00	1.00	100	95.54	0.82	0.99 (0.97–1)	0.96

TP: True Positive; FP: False Positive; TN: True Negative; FN: False Negative; Sens: Sensitivity; Spec: Specificity; Acc: Accuracy; MCC: Matthews Correlation Coefficient; AUROC: Area under Receiver operating characteristic curve; CI: Confidence Interval.

Gene enrichment analysis revealed that *RELN* and *RPS6KA5* are associated with activation of cyclic AMP (cAMP) response element-binding protein (*CREB*) transcription factor (adjusted p-value<0.01), which is responsible for tumor initiation, progression, and metastasis [58]. *RELN* is an extracellular glycoprotein that plays a vital role in neuronal migration and has been shown to be downregulated in many cancers [59, 60].

### Web server implementation

We established a web server, CancerTSP (Thyroid cancer stage prediction), that implements models developed in the present study for investigation and estimation of cancer stage from the transcripts’ expression data. The CancerTSP consists of two key modules consisting of prediction and analysis.

The prediction module consists of two modules for predicting the stage of the THCA cancer sample. One of the models is based on protein-coding features (THCA-EL-PC), and the other model is based on both protein-coding and non-protein coding transcripts (THCA-All-SVC-L1). We also provide a third prediction module based on the THCA-NEL-M features, which can predict whether the sample is normal, early-stage, or late-stage sample. The results are displayed on the score thresholds that exhibited a minimum difference in recall and specificity with maximum accuracy. The user can change the threshold for less or more stringent results in terms of recall and specificity. The lower threshold will increase the recall but decrease the specificity and a higher threshold will do the opposite to prediction outcomes (S8 Fig).

The user needs to provide transcript expression (FPKM values) of potential biomarker genes for every patient. The number of patients corresponds to the number of columns in a file. The output includes a list of patients and corresponding predicting stage of cancer (early or late-stage) along with the prediction score (probability value).

Another module is dedicated for analysis which is helpful in evaluating the role of each transcript in discrimination of early-stage from the late-stage. This module gives p-value (calculated using Wilcoxon rank test) for each transcript that signifies whether the transcript’s expression varies in the early and late-stage significantly. It also gives expression threshold and classifying AUROC of each transcript along with the average expression of that gene in the early and late-stage of cancer. The CancerTSP webserver is available from URL http://webs.iiitd.edu.in/raghava/cancertsp/ for public use.

## Discussion

The current study is an attempt for the identification of reliable RNA expression-based genomic markers that are capable of segregating early-stage patients from late-stage patients of thyroid cancer. Despite the benefits of FNA for diagnosing papillary, medullary, and anaplastic thyroid cancer, it has limited utility in determining the stage and benign or malignant status of thyroid tumors. In addition, some FNA results suggest but not definitively diagnose papillary thyroid cancer [61]. The diagnosis of patients at an early stage aids the application of adequate treatments and disease management which eventually improves the outcome of the patients. With the advent of genomics technology, publicly available cancer patients’ expression data from resources like GDC and GEO has expedited the search for expression-based molecular markers capable of reliable diagnosis in clinical settings.

In the current study, we tried to understand how well (prediction power in terms of AUROC) the expression of a gene or RNA transcript can predict the stage of the PTC tumor samples. First, we ranked all the transcripts on the basis of AUROC, calculated based on simple expression-based threshold models. Here, the expression of a single gene, i.e., *DCN*, at the threshold of 3.01 (log2 FPKM), showed maximum AUROC of 0.66. The *DCN* gene is a member of the extracellular small leucine-rich proteoglycan family present in connective tissues. Arnaldi et al. showed that *DCN* could be a potential diagnostic marker and therapeutic target for PTC [41]. It also has been shown that increased expression of *DCN* leads to decreased adhesion and increased migration of glioma cells by downregulation of TGF-β signaling [62].

Furthermore, the gene-enrichment analysis of 166 protein-coding genes from the THCA-EL-AUROC signature set revealed their significant enrichment in various KEGG pathways including the Focal adhesion pathway, PI3K-Akt signaling pathway, and Proteoglycans in cancer, etc. Notably, the Focal adhesion kinase has already been shown to be overexpressed in thyroid cancers [63]. There is a plethora of literature that indicates that PI3K-Akt signaling pathway components are dysregulated in cancers [64–66]. Further, there is extensive remodeling of tumor stroma, which is related with noticeable variations in proteoglycans expression and structural variability. Proteoglycans mainly contribute to the formation of a matrix for tumor growth affecting tissue organization [67]. Thus, the previous literature and enrichment analysis indicate that these prioritized genes are involved in various cancer progression related processes and, therefore, can be explored as potential biomarkers of stage classification. However, more research on large cohorts is warranted to confirm this hypothesis.

Next, various combinations are tested to elucidate potential biomarker subset for segregating early and late-stage samples. Towards this, we explored various feature spaces like protein-coding transcripts only, cancer hallmark transcripts, and both types of transcripts (protein-coding and non-coding transcripts) from the 60,483 RNA transcripts. The SVC model based on the THCA-EL-All-WEKA (78 features), resulted in F1 score of 0.70 on the validation data. The various types of features in this signature reveal the role of various non-coding transcripts along with protein-coding transcripts in the progression of cancer. Out of 28 protein-coding, five genes; *TERT* [68], *FLT4* [69], *DUSP6* [70], *USP10* [71] and *POMC* [72] have already been implicated in thyroid cancer. miR-3196 has also been found to be downregulated in PTC non-metastasized patients [73]. This shows that many components out of 78 signatures have already been implicated in the PTC and other malignancies. These genes can be further investigated to reveal their role as biomarkers for early-stage of PTC. The SVC model based on 36-features set (THCA-EL-SVC-L1) selected using the SVC-L1 feature selection method, is the top performer in categorizing early and late-stage samples of the validation dataset with F1 score of 0.75, and resulted in the reduction of the features nearly half as compared to 78 features. Further, the performance of 18 features from this panel was also validated using cross-platform normalization on the external validation data. 70% early-stage samples (Sensitivity) and 100% late-stage samples (specificity) from the external validation dataset were correctly predicted. One of the most studied genes, *TERT*, is part of this signature and its promoter mutations are closely associated with aggressive clinicopathological characteristics and poor prognosis in PTC earlier [74]. Next, we also developed the multiclass machine learning prediction models to distinguish normal, early, and late samples. The SVC model based on the THCA-NEL-M signature of 107 transcripts attained F1 score of 0.88, 0.78, and 0.55 for normal, early, and late stage classes, respectively on the validation dataset.

Additionally, RNA transcripts having high prediction capability in terms of AUROC for categorizing cancer and normal samples also have been derived. Interestingly overlapping sense transcript *RP11-363E7*.*4* showed the highest AUROC of 0.96 in classifying cancer samples from normal samples. It has been already demonstrated in the literature that sense to antisense transcript ratio increases in cancer [75]. Other protein-coding transcript *FAM84A* shows 0.95 AUROC and has already been reported to play a role in metastasis of liver and colon cancer [76, 77]. In this study, AUROC of most of the signatures to segregate cancer and normal samples have a similar range as reported by earlier studies, which further validates our findings [78]. Further, using five protein-coding transcripts (THCA-CL-PC), we were able to classify cancer samples from normal samples in the validation dataset with F1 score of 0.97.

Eventually, a web server CancerTSP is developed, where the user can provide the transcripts’ expression (FPKM values) and can predict whether the cancer is in the early or late stage. This type of application where expression of transcripts is used to demarcate the early and late stage of cancer using machine learning can provide better understandings about the role of diverse transcripts responsible for the development of cancer from early to the late stage. Hence, this resource will help the scientific community in making preliminary hypotheses regarding cancer progression.

## Conclusion

In conclusion, 36 RNA-transcripts based SVC prediction model attained considerable performance in segregating the early-stage and late-stage PTC tissue samples with F1 score of 0.75. In addition, prediction models based on five protein-coding transcripts categorized tumorous and non-tumorous samples of patients with high F1 score of 0.97. Eventually, all prediction models based on identified candidate markers are integrated into CancerTSP webserver for the classification of early-stage from late-stage and PTC tumors from normal samples to facilitate the research community engaged in this field. We anticipate the current study might prove to be helpful in recognition of the potential of these new transcriptomic markers for early diagnosis of PTC. Additionally, further investigation of these markers on larger cohorts is required to confirm their potential clinical utility.

### Limitation of the study

In this study, we have scrutinized potential transcriptomic signatures to distinguish early and late-stage samples of PTC. One of the limitations associated with these signatures is that they are derived from tissue samples only, which is an invasive technique for biomarker discovery. Further, the external dataset used in the current study contains only 20 samples. Thus, field can be advanced by adopting non-invasive biomarkers from specimens like blood, urine, cell-free DNA, etc. along with the validation on large sampled cohorts to confirm their clinical utility.

## Supporting information

S1 Table Number of different types of 60,483 transcripts according to GENCODE version 22. (DOCX)

S2 Table Transcripts with AUROC greater than 0.60 for differentiation between early and late stage samples. (DOCX)

S3 Table Oncogenic signatures enriched in the transcripts showing area under the curve differentiation between early and late stage. (DOCX)

S4 Table Performance measures of 179 transcripts set selected by expression threshold based AUROC ranking on training model and independent validation dataset by implementing SVC using Scikit and various other machine-learning algorithms. (DOCX)

S5 Table List of 100 protein coding transcripts selected using F_ANOVA. (DOCX)

S6 Table Performance measures of 100 protein coding mRNA feature set (THCA-EL-F-PC) selected by F_ANOVA method on training model and independent validation dataset by implementing various machine-learning algorithms. (DOCX)

S7 Table Performance of SVC based models and WEKA based models on the hallmark transcripts selected by WEKA (THCA-EL-H) on training dataset and independent validation dataset. (DOCX)

S8 Table Performance measures of 50 hallmark protein transcripts (THCA-EL-FH) feature set selected by F_ANOVA method on training model and independent validation dataset by implementing SVC using Scikit and various other machine-learning algorithms using WEKA. (DOCX)

S9 Table Performance measures of 78-full feature set (THA-EL-All-WEKA) selected by FCBF-WEKA on training model and independent validation dataset by implementing various machine-learning algorithms. (DOCX)

S10 Table Features selected from WEKA based feature selection using all types of transcripts (THCA-EL-All). (DOCX)

S11 Table The 100 F_ANOVA features selected from all types of transcripts (THCA-EL-All-F). (DOCX)

S12 Table Performance measures of 100 (THCA-EL-All-F) all transcripts set selected by F_ANOVA method on training model and independent validation dataset by implementing SVC using Scikit and various other machine-learning algorithms using WEKA. (DOCX)

S13 Table 27 transcripts (THCA-EL-CODO) selected using F_ANOVA at first step and a wrapper based approach combining human opinion dynamics optimizer and SVC at second steps. (DOCX)

S14 Table Performance measures of 27-full features (THCA-EL-CODO) set selected by human opinion dynamics wrapper approach on training model and independent validation dataset. (DOCX)

S15 Table 36 features selected using SVC-L1 feature selection. (DOCX)

S16 Table 107 RNA transcripts selected using SVC L1 for multiclass classification (THCA-NEL-M). (DOCX)

S17 Table Top genes showing AUROC of > = 0.85 for differentiation between cancer and normal samples. (DOCX)

S1 Fig Clinical characteristics of patients used in the study. (PNG)

S2 Fig The Dispersion Separability Criterion (DSC) value for THCA TCGA calculated using TCGA batch effects viewer. (PNG)

S3 Fig Top transcripts with highest AUROC of differentiation in early and late stage samples. (TIFF)

S4 Fig Gene ontology analysis of 166 enriched protein coding transcripts having AUROC of differentiating early and late stage patients greater than 0.6. (PNG)

S5 Fig Interaction network in the string database of connected nodes out of 37 protein coding features used for early and late stage classification. The coloured nodes represent the genes in the signature and white nodes are indirect neighbours added to the network.
(PNG)

S6 Fig Gene ontology and pathway analysis of 386 enriched protein coding transcripts having AUC of differentiating Cancer and normal stage patients greater than 0.85. (PNG)

S7 Fig The AUROC plot comparing performance obtained using 500 cancer samples (THCA-CN-F), 58 paired cancer samples (THCA-CN-P) and 58 random cancer samples (THCA-CN-R) in comparison to 58 normal samples for segregating cancer and normal samples. (PNG)

S8 Fig The threshold of the selected models on the webservers (a) all transcript (THCA-EL-SVC-L1) training model (b) all transcript (THCA-EL-SVC-L1) validation model (c) protein coding (THCA-EL-PC) training model (d) protein coding (THCA-EL-PC) validation model (e) Cancer Normal (THCA-CN-F) training model (f) Cancer Normal (THCA-CN-F) validation model.
(PNG)

S1 Dataset (ZIP)
