# Supplementary material for: Expression based biomarkers and models to classify early and late-stage samples of Papillary Thyroid Carcinoma
Source: PLoS One. 2020 Apr 23;15(4):e0231629. doi: 10.1371/journal.pone.0231629 (PMC7179925; doi:10.1371/journal.pone.0231629)
Supplement: S1 Table — (DOCX) [file pone.0231629.s001.docx]

Table S1 : Number of different types of 60,483 transcripts according to GENCODE version 22

| **S.No** | **Type of Transcript** | **Number of transcripts** |
| --- | --- | --- |
| 1 | protein_coding | 19814 |
| 2 | processed_pseudogene | 10304 |
| 3 | lincRNA | 7656 |
| 4 | antisense | 5565 |
| 5 | miRNA | 4093 |
| 6 | unprocessed_pseudogene | 2574 |
| 7 | misc_RNA | 2298 |
| 8 | snRNA | 1896 |
| 9 | TEC | 1045 |
| 10 | snoRNA | 961 |
| 11 | sense_intronic | 920 |
| 12 | transcribed_unprocessed_pseudogene | 663 |
| 13 | rRNA | 544 |
| 14 | processed_transcript | 484 |
| 15 | transcribed_processed_pseudogene | 443 |
| 16 | sense_overlapping | 197 |
| 17 | IG_V_pseudogene | 180 |
| 18 | unitary_pseudogene | 169 |
| 19 | IG_V_gene | 142 |
| 20 | TR_V_gene | 106 |
| 21 | TR_J_gene | 73 |
| 22 | polymorphic_pseudogene | 59 |
| 23 | scaRNA | 49 |
| 24 | IG_D_gene | 37 |
| 25 | pseudogene | 36 |
| 26 | TR_V_pseudogene | 30 |
| 27 | 3prime_overlapping_ncrna | 29 |
| 28 | Mt_tRNA | 22 |
| 29 | sRNA | 20 |
| 30 | IG_J_gene | 18 |
| 31 | IG_C_gene | 14 |
| 32 | IG_C_pseudogene | 9 |
| 33 | ribozyme | 8 |
| 34 | TR_C_gene | 5 |
| 35 | TR_J_pseudogene | 4 |
| 36 | IG_J_pseudogene | 3 |
| 37 | non_coding | 3 |
| 38 | TR_D_gene | 3 |
| 39 | Mt_rRNA | 2 |
| 40 | macro_lncRNA | 1 |
| 41 | transcribed_unitary_pseudogene | 1 |
| 42 | translated_processed_pseudogene | 1 |
| 43 | translated_unprocessed_pseudogene | 1 |
| 44 | vaultRNA | 1 |
