# Supplementary material for: Expression based biomarkers and models to classify early and late-stage samples of Papillary Thyroid Carcinoma
Source: PLoS One. 2020 Apr 23;15(4):e0231629. doi: 10.1371/journal.pone.0231629 (PMC7179925; doi:10.1371/journal.pone.0231629)
Supplement: S2 Table — (DOCX) [file pone.0231629.s002.docx]

Table S2: Transcripts with AUROC greater than 0.60 for differentiation between early and late stage samples.

| **Transcript ID** | **Transcript type** | **Mapped HGNC symbol** | **Mean in Early stage patients** | **Mean in Late stage patients** | **Accuracy** | **AUROC** | **Threshold** | **Adjusted p-value** |
| --- | --- | --- | --- | --- | --- | --- | --- | --- |
| **ENSG00000064205.9** | protein_coding | WISP2 | 0.38 | 0.89 | 70.85 | 0.62 | less than 1.00 | 0.000146591 |
| **ENSG00000254343.2** | lincRNA | RP11-760H22.2 | 3.26 | 2.69 | 63.57 | 0.63 | more than 2.87 | 0.000603974 |
| **ENSG00000123500.8** | protein_coding | COL10A1 | 0.93 | 1.87 | 71.36 | 0.64 | less than 2.00 | 0.000809487 |
| **ENSG00000155792.8** | protein_coding | DEPTOR | 4.23 | 3.57 | 56.53 | 0.61 | more than 4.22 | 0.000854368 |
| **ENSG00000159166.12** | protein_coding | LAD1 | 3.57 | 4.13 | 64.82 | 0.62 | less than 4.25 | 0.001273911 |
| **ENSG00000178163.6** | protein_coding | ZNF518B | 1.8 | 1.48 | 72.11 | 0.61 | more than 1.21 | 0.001390877 |
| **ENSG00000104918.6** | protein_coding | RETN | 0.61 | 1.17 | 68.09 | 0.63 | less than 1.00 | 0.001410445 |
| **ENSG00000197614.9** | protein_coding | MFAP5 | 0.67 | 1.34 | 67.59 | 0.64 | less than 1.00 | 0.003027578 |
| **ENSG00000145423.4** | protein_coding | SFRP2 | 2.91 | 4.39 | 62.06 | 0.64 | less than 3.00 | 0.006261291 |
| **ENSG00000104808.6** | protein_coding | DHDH | 0.94 | 1.33 | 59.3 | 0.62 | less than 1.00 | 0.006773445 |
| **ENSG00000060718.17** | protein_coding | COL11A1 | 0.44 | 1.24 | 70.6 | 0.63 | less than 1.00 | 0.007004765 |
| **ENSG00000137745.10** | protein_coding | MMP13 | 0.29 | 0.88 | 70.1 | 0.6 | less than 1.00 | 0.007327267 |
| **ENSG00000166455.12** | protein_coding | C16orf46 | 1.53 | 1.29 | 68.09 | 0.61 | more than 1.13 | 0.009349647 |
| **ENSG00000037280.14** | protein_coding | FLT4 | 2.24 | 1.8 | 56.28 | 0.61 | more than 2.33 | 0.011416456 |
| **ENSG00000151883.15** | protein_coding | PARP8 | 2.87 | 2.61 | 71.11 | 0.6 | more than 2.31 | 0.012269238 |
| **ENSG00000172061.8** | protein_coding | LRRC15 | 0.49 | 1.25 | 68.34 | 0.62 | less than 1.00 | 0.012357178 |
| **ENSG00000139329.4** | protein_coding | LUM | 3.47 | 4.57 | 69.1 | 0.64 | less than 5.07 | 0.015671244 |
| **ENSG00000129038.14** | protein_coding | LOXL1 | 1.99 | 2.45 | 63.82 | 0.63 | less than 2.22 | 0.016542179 |
| **ENSG00000180543.4** | protein_coding | TSPYL5 | 2.77 | 2.44 | 68.09 | 0.61 | more than 2.35 | 0.019069775 |
| **ENSG00000106819.10** | protein_coding | ASPN | 2.17 | 2.96 | 69.35 | 0.62 | less than 3.02 | 0.019351366 |
| **ENSG00000126217.19** | protein_coding | MCF2L | 1.43 | 1.22 | 64.57 | 0.64 | more than 1.25 | 0.023117995 |
| **ENSG00000163762.5** | protein_coding | TM4SF18 | 2.21 | 1.81 | 60.8 | 0.63 | more than 2.13 | 0.023400187 |
| **ENSG00000168152.11** | protein_coding | THAP9 | 1.49 | 1.33 | 64.07 | 0.62 | more than 1.33 | 0.024563625 |
| **ENSG00000137713.14** | protein_coding | PPP2R1B | 2.07 | 1.93 | 54.02 | 0.61 | more than 2.10 | 0.024923129 |
| **ENSG00000127083.7** | protein_coding | OMD | 0.67 | 1.23 | 66.33 | 0.63 | less than 1.00 | 0.030944649 |
| **ENSG00000183688.4** | protein_coding | FAM101B | 2.97 | 2.56 | 55.53 | 0.62 | more than 3.14 | 0.03171918 |
| **ENSG00000102970.9** | protein_coding | CCL17 | 1.56 | 2.44 | 55.53 | 0.61 | less than 1.00 | 0.035418102 |
| **ENSG00000271147.6** | processed_transcript | RP4-769N13.6 | 1.25 | 1.11 | 67.34 | 0.6 | more than 1.04 | 0.036710915 |
| **ENSG00000102359.5** | protein_coding | SRPX2 | 0.85 | 1.39 | 67.59 | 0.65 | less than 1.03 | 0.036974528 |
| **ENSG00000011465.15** | protein_coding | DCN | 2.18 | 3.03 | 68.59 | 0.66 | less than 3.01 | 0.037329002 |
| **ENSG00000166741.6** | protein_coding | NNMT | 2.16 | 2.83 | 60.3 | 0.63 | less than 2.16 | 0.042050945 |
| **ENSG00000145703.14** | protein_coding | IQGAP2 | 2.4 | 1.84 | 69.85 | 0.62 | more than 1.14 | 0.046900048 |
| **ENSG00000198542.12** | protein_coding | ITGBL1 | 0.66 | 1.12 | 69.35 | 0.65 | less than 1.00 | 0.047233706 |
| **ENSG00000106483.10** | protein_coding | SFRP4 | 1.76 | 2.71 | 62.31 | 0.62 | less than 2.00 | 0.048707543 |
| **ENSG00000122861.14** | protein_coding | PLAU | 4.82 | 5.66 | 68.84 | 0.63 | less than 6.34 | 0.057177372 |
| **ENSG00000105483.15** | protein_coding | CARD8 | 1.86 | 1.67 | 60.8 | 0.6 | more than 1.72 | 0.058260984 |
| **ENSG00000186340.13** | protein_coding | THBS2 | 2.12 | 2.92 | 69.85 | 0.64 | less than 3.22 | 0.063098112 |
| **ENSG00000087095.11** | protein_coding | NLK | 2.89 | 2.75 | 59.05 | 0.62 | more than 2.84 | 0.073953715 |
| **ENSG00000112715.19** | protein_coding | VEGFA | 4.64 | 4.13 | 67.09 | 0.61 | more than 3.86 | 0.089447101 |
| **ENSG00000138399.16** | protein_coding | FASTKD1 | 2.08 | 1.9 | 55.53 | 0.6 | more than 2.06 | 0.090275006 |
| **ENSG00000127663.13** | protein_coding | KDM4B | 2.46 | 2.28 | 62.06 | 0.61 | more than 2.28 | 0.095192888 |
| **ENSG00000237172.3** | protein_coding | B3GNT9 | 4.18 | 3.92 | 65.08 | 0.6 | more than 3.90 | 0.098535507 |
| **ENSG00000108821.12** | protein_coding | COL1A1 | 4.96 | 6.02 | 64.32 | 0.62 | less than 5.81 | 0.102700805 |
| **ENSG00000275395.3** | protein_coding | FCGBP | 5 | 4.42 | 57.04 | 0.61 | more than 4.81 | 0.108513875 |
| **ENSG00000230838.1** | lincRNA | AC093850.2 | 0.4 | 0.86 | 70.85 | 0.61 | less than 1.00 | 0.134861387 |
| **ENSG00000133048.11** | protein_coding | CHI3L1 | 4.73 | 6.08 | 56.53 | 0.6 | less than 5.03 | 0.139466671 |
| **ENSG00000149090.10** | protein_coding | PAMR1 | 1.23 | 0.97 | 59.05 | 0.6 | more than 1.10 | 0.144612674 |
| **ENSG00000130201.6** | protein_coding | EXOC3L2 | 3.62 | 3.08 | 64.82 | 0.62 | more than 3.05 | 0.16562433 |
| **ENSG00000124191.16** | protein_coding | TOX2 | 1.59 | 1.33 | 64.57 | 0.62 | more than 1.30 | 0.195575762 |
| **ENSG00000149346.13** | protein_coding | SLX4IP | 1.42 | 1.32 | 54.27 | 0.61 | more than 1.48 | 0.209146751 |
| **ENSG00000265458.1** | antisense | RP13-20L14.6 | 1.26 | 1.05 | 55.78 | 0.6 | more than 1.23 | 0.217219747 |
| **ENSG00000047346.11** | protein_coding | FAM214A | 2.68 | 2.47 | 57.29 | 0.61 | more than 2.71 | 0.243830979 |
| **ENSG00000106333.11** | protein_coding | PCOLCE | 3 | 3.49 | 56.78 | 0.62 | less than 2.86 | 0.244369876 |
| **ENSG00000047648.20** | protein_coding | ARHGAP6 | 2.38 | 2.11 | 61.31 | 0.61 | more than 2.23 | 0.246545872 |
| **ENSG00000080802.17** | protein_coding | CNOT4 | 2.26 | 2.12 | 60.8 | 0.61 | more than 2.22 | 0.255420904 |
| **ENSG00000077942.16** | protein_coding | FBLN1 | 2.09 | 2.83 | 64.57 | 0.63 | less than 2.32 | 0.259968359 |
| **ENSG00000106624.7** | protein_coding | AEBP1 | 4.37 | 4.89 | 63.57 | 0.61 | less than 4.90 | 0.262277499 |
| **ENSG00000116031.8** | protein_coding | CD207 | 1.24 | 1.82 | 59.3 | 0.61 | less than 1.00 | 0.271087905 |
| **ENSG00000133055.7** | protein_coding | MYBPH | 0.71 | 1.16 | 65.08 | 0.61 | less than 1.00 | 0.30998813 |
| **ENSG00000117461.13** | protein_coding | PIK3R3 | 2.65 | 2.32 | 60.3 | 0.6 | more than 2.47 | 0.329700628 |
| **ENSG00000143387.11** | protein_coding | CTSK | 3.38 | 3.98 | 62.06 | 0.6 | less than 3.79 | 0.332599579 |
| **ENSG00000166669.12** | protein_coding | ATF7IP2 | 1.24 | 1.03 | 60.55 | 0.62 | more than 1.13 | 0.339951383 |
| **ENSG00000120833.12** | protein_coding | SOCS2 | 1.62 | 1.39 | 64.57 | 0.62 | more than 1.34 | 0.361393288 |
| **ENSG00000054690.12** | protein_coding | PLEKHH1 | 3.22 | 2.83 | 66.08 | 0.6 | more than 2.70 | 0.39087085 |
| **ENSG00000167874.6** | protein_coding | TMEM88 | 3.21 | 2.72 | 65.08 | 0.63 | more than 2.70 | 0.410007275 |
| **ENSG00000158477.6** | protein_coding | CD1A | 1.07 | 1.65 | 61.56 | 0.61 | less than 1.00 | 0.445925315 |
| **ENSG00000258227.5** | protein_coding | CLEC5A | 0.91 | 1.18 | 60.55 | 0.61 | less than 1.00 | 0.449015825 |
| **ENSG00000179954.13** | protein_coding | SSC5D | 0.87 | 1.22 | 65.58 | 0.62 | less than 1.10 | 0.462796499 |
| **ENSG00000103966.8** | protein_coding | EHD4 | 3.69 | 3.5 | 65.08 | 0.61 | more than 3.47 | 0.472879149 |
| **ENSG00000162366.6** | protein_coding | PDZK1IP1 | 3.78 | 4.81 | 62.06 | 0.6 | less than 5.01 | 0.502250094 |
| **ENSG00000124772.10** | protein_coding | CPNE5 | 1.44 | 1.19 | 59.55 | 0.61 | more than 1.25 | 0.524313782 |
| **ENSG00000163472.17** | protein_coding | TMEM79 | 2.54 | 2.92 | 63.07 | 0.62 | less than 2.95 | 0.573608544 |
| **ENSG00000185115.4** | protein_coding | NDNL2 | 4.03 | 3.84 | 63.57 | 0.61 | more than 3.85 | 0.606326309 |
| **ENSG00000262655.3** | protein_coding | SPON1 | 0.9 | 1.42 | 64.32 | 0.62 | less than 1.02 | 0.632692679 |
| **ENSG00000128052.8** | protein_coding | KDR | 3.87 | 3.37 | 53.27 | 0.6 | more than 4.28 | 0.63808728 |
| **ENSG00000051108.13** | protein_coding | HERPUD1 | 5 | 4.76 | 65.83 | 0.61 | more than 4.63 | 0.640789739 |
| **ENSG00000179144.4** | protein_coding | GIMAP7 | 3.8 | 3.39 | 65.33 | 0.61 | more than 3.28 | 0.648997575 |
| **ENSG00000110075.13** | protein_coding | PPP6R3 | 3.81 | 3.67 | 66.83 | 0.61 | more than 3.64 | 0.66857255 |
| **ENSG00000115414.17** | protein_coding | FN1 | 7.85 | 8.95 | 67.09 | 0.61 | less than 10.12 | 0.759035953 |
| **ENSG00000177707.9** | protein_coding | PVRL3 | 2.72 | 2.35 | 60.3 | 0.6 | more than 2.43 | 0.808488521 |
| **ENSG00000222047.7** | protein_coding | C10orf55 | 0.74 | 1.02 | 64.32 | 0.6 | less than 1.01 | 0.815304683 |
| **ENSG00000121058.4** | protein_coding | COIL | 2.98 | 2.85 | 62.31 | 0.61 | more than 2.89 | 0.850255517 |
| **ENSG00000158481.11** | protein_coding | CD1C | 1.22 | 1.75 | 57.79 | 0.6 | less than 1.00 | 0.852028111 |
| **ENSG00000187730.7** | protein_coding | GABRD | 2.17 | 1.76 | 56.28 | 0.6 | more than 2.14 | 0.877420673 |
| **ENSG00000189377.7** | protein_coding | CXCL17 | 2.48 | 3.29 | 60.55 | 0.6 | less than 3.00 | 0.886649038 |
| **ENSG00000147642.15** | protein_coding | SYBU | 1.45 | 1.18 | 62.31 | 0.6 | more than 1.11 | 0.894061714 |
| **ENSG00000159640.13** | protein_coding | ACE | 2.09 | 1.76 | 66.33 | 0.61 | more than 1.47 | 0.90532138 |
| **ENSG00000167123.17** | protein_coding | CERCAM | 3.06 | 3.35 | 59.55 | 0.6 | less than 3.21 | 0.907191144 |
| **ENSG00000102802.8** | protein_coding | MEDAG | 2.52 | 2.96 | 65.83 | 0.63 | less than 3.15 | 0.936055197 |
| **ENSG00000006638.10** | protein_coding | TBXA2R | 1.37 | 1.13 | 59.05 | 0.61 | more than 1.25 | 1 |
| **ENSG00000006831.9** | protein_coding | ADIPOR2 | 3.82 | 3.6 | 58.04 | 0.61 | more than 3.75 | 1 |
| **ENSG00000008952.15** | protein_coding | SEC62 | 4.96 | 4.79 | 61.06 | 0.62 | more than 4.88 | 1 |
| **ENSG00000009765.13** | protein_coding | IYD | 5.65 | 5.02 | 61.31 | 0.6 | more than 5.30 | 1 |
| **ENSG00000038427.14** | protein_coding | VCAN | 1.33 | 1.93 | 67.59 | 0.62 | less than 2.02 | 1 |
| **ENSG00000070610.13** | protein_coding | GBA2 | 3.91 | 3.73 | 67.84 | 0.61 | more than 3.61 | 1 |
| **ENSG00000075223.12** | protein_coding | SEMA3C | 0.76 | 1.09 | 65.33 | 0.61 | less than 1.03 | 1 |
| **ENSG00000078098.12** | protein_coding | FAP | 0.67 | 1.14 | 68.09 | 0.63 | less than 1.03 | 1 |
| **ENSG00000084733.9** | protein_coding | RAB10 | 5.25 | 5.11 | 60.8 | 0.6 | more than 5.17 | 1 |
| **ENSG00000087116.12** | protein_coding | ADAMTS2 | 0.95 | 1.27 | 63.82 | 0.61 | less than 1.07 | 1 |
| **ENSG00000088826.16** | protein_coding | SMOX | 2.2 | 2.05 | 67.59 | 0.6 | more than 1.73 | 1 |
| **ENSG00000099940.10** | protein_coding | SNAP29 | 3.03 | 2.95 | 61.31 | 0.62 | more than 3.03 | 1 |
| **ENSG00000101665.7** | protein_coding | SMAD7 | 3.66 | 3.42 | 62.06 | 0.61 | more than 3.48 | 1 |
| **ENSG00000103855.16** | protein_coding | CD276 | 3.63 | 3.85 | 62.81 | 0.6 | less than 3.94 | 1 |
| **ENSG00000105664.9** | protein_coding | COMP | 2.57 | 3.4 | 61.81 | 0.62 | less than 3.00 | 1 |
| **ENSG00000106070.16** | protein_coding | GRB10 | 2.84 | 2.57 | 54.52 | 0.6 | more than 2.91 | 1 |
| **ENSG00000106809.9** | protein_coding | OGN | 0.72 | 1.18 | 64.57 | 0.6 | less than 1.00 | 1 |
| **ENSG00000111799.19** | protein_coding | COL12A1 | 1.31 | 1.84 | 70.35 | 0.62 | less than 2.11 | 1 |
| **ENSG00000117640.16** | protein_coding | MTFR1L | 3.59 | 3.43 | 59.55 | 0.62 | more than 3.55 | 1 |
| **ENSG00000122691.11** | protein_coding | TWIST1 | 0.77 | 1.05 | 66.58 | 0.61 | less than 1.03 | 1 |
| **ENSG00000123610.4** | protein_coding | TNFAIP6 | 0.8 | 1.1 | 65.58 | 0.61 | less than 1.00 | 1 |
| **ENSG00000125869.8** | protein_coding | LAMP5 | 2.03 | 2.61 | 60.05 | 0.62 | less than 2.00 | 1 |
| **ENSG00000126368.5** | protein_coding | NR1D1 | 4.11 | 4.51 | 57.29 | 0.6 | less than 4.21 | 1 |
| **ENSG00000130413.14** | protein_coding | STK33 | 1.78 | 1.65 | 69.6 | 0.6 | more than 1.46 | 1 |
| **ENSG00000130600.14** | processed_transcript | H19 | 1.01 | 1.54 | 60.55 | 0.6 | less than 1.00 | 1 |
| **ENSG00000130635.14** | protein_coding | COL5A1 | 2.14 | 2.74 | 62.06 | 0.61 | less than 2.30 | 1 |
| **ENSG00000131018.21** | protein_coding | SYNE1 | 1.49 | 1.23 | 62.31 | 0.6 | more than 1.14 | 1 |
| **ENSG00000131459.11** | protein_coding | GFPT2 | 0.94 | 1.21 | 64.07 | 0.62 | less than 1.06 | 1 |
| **ENSG00000132031.11** | protein_coding | MATN3 | 0.5 | 0.81 | 70.6 | 0.6 | less than 1.02 | 1 |
| **ENSG00000132386.9** | protein_coding | SERPINF1 | 3.1 | 3.66 | 64.07 | 0.62 | less than 3.66 | 1 |
| **ENSG00000133110.13** | protein_coding | POSTN | 2.96 | 3.87 | 68.84 | 0.63 | less than 4.15 | 1 |
| **ENSG00000133169.5** | protein_coding | BEX1 | 2.85 | 2.26 | 62.81 | 0.6 | more than 2.04 | 1 |
| **ENSG00000135218.16** | protein_coding | CD36 | 1.8 | 1.31 | 64.07 | 0.6 | more than 1.03 | 1 |
| **ENSG00000135549.13** | protein_coding | PKIB | 0.82 | 1.04 | 66.83 | 0.6 | less than 1.09 | 1 |
| **ENSG00000137502.8** | protein_coding | RAB30 | 1.6 | 1.42 | 65.33 | 0.6 | more than 1.30 | 1 |
| **ENSG00000137673.7** | protein_coding | MMP7 | 1.57 | 2.32 | 64.32 | 0.61 | less than 2.00 | 1 |
| **ENSG00000137809.15** | protein_coding | ITGA11 | 0.62 | 0.9 | 68.59 | 0.6 | less than 1.01 | 1 |
| **ENSG00000140274.12** | protein_coding | DUOXA2 | 3.72 | 3.16 | 62.81 | 0.6 | more than 3.05 | 1 |
| **ENSG00000142552.6** | protein_coding | RCN3 | 2.41 | 2.8 | 64.57 | 0.61 | less than 2.77 | 1 |
| **ENSG00000142669.12** | protein_coding | SH3BGRL3 | 6.49 | 6.78 | 56.28 | 0.61 | less than 6.43 | 1 |
| **ENSG00000144791.8** | protein_coding | LIMD1 | 2.41 | 2.22 | 56.03 | 0.61 | more than 2.49 | 1 |
| **ENSG00000144843.10** | protein_coding | ADPRH | 2.05 | 1.91 | 58.79 | 0.6 | more than 2.04 | 1 |
| **ENSG00000149428.17** | protein_coding | HYOU1 | 4.52 | 4.14 | 60.3 | 0.61 | more than 4.32 | 1 |
| **ENSG00000153767.8** | protein_coding | GTF2E1 | 1.76 | 1.64 | 61.56 | 0.6 | more than 1.68 | 1 |
| **ENSG00000153822.12** | protein_coding | KCNJ16 | 6.24 | 5.85 | 67.59 | 0.6 | more than 5.68 | 1 |
| **ENSG00000154133.13** | protein_coding | ROBO4 | 2.71 | 2.36 | 57.54 | 0.62 | more than 2.75 | 1 |
| **ENSG00000154144.11** | protein_coding | TBRG1 | 2.37 | 2.27 | 65.08 | 0.6 | more than 2.25 | 1 |
| **ENSG00000158270.11** | protein_coding | COLEC12 | 0.87 | 1.21 | 63.57 | 0.61 | less than 1.02 | 1 |
| **ENSG00000162639.14** | protein_coding | HENMT1 | 1.23 | 1.06 | 58.54 | 0.61 | more than 1.17 | 1 |
| **ENSG00000163171.7** | protein_coding | CDC42EP3 | 2.57 | 2.93 | 60.8 | 0.6 | less than 2.81 | 1 |
| **ENSG00000163359.14** | protein_coding | COL6A3 | 2.43 | 3.05 | 67.84 | 0.6 | less than 3.42 | 1 |
| **ENSG00000163389.9** | protein_coding | POGLUT1 | 1.63 | 1.53 | 59.8 | 0.61 | more than 1.60 | 1 |
| **ENSG00000163520.12** | protein_coding | FBLN2 | 2.88 | 3.24 | 65.08 | 0.61 | less than 3.43 | 1 |
| **ENSG00000163864.13** | protein_coding | NMNAT3 | 1.21 | 1.11 | 61.56 | 0.6 | more than 1.05 | 1 |
| **ENSG00000163884.3** | protein_coding | KLF15 | 1.43 | 1.21 | 60.05 | 0.6 | more than 1.17 | 1 |
| **ENSG00000164078.11** | protein_coding | MST1R | 1.64 | 1.87 | 64.32 | 0.6 | less than 2.09 | 1 |
| **ENSG00000164692.16** | protein_coding | COL1A2 | 4.78 | 5.52 | 67.09 | 0.61 | less than 5.81 | 1 |
| **ENSG00000164935.5** | protein_coding | DCSTAMP | 3.56 | 4.64 | 59.8 | 0.61 | less than 5.00 | 1 |
| **ENSG00000165113.11** | protein_coding | GKAP1 | 2.11 | 1.97 | 66.58 | 0.6 | more than 1.90 | 1 |
| **ENSG00000166582.8** | protein_coding | CENPV | 2.08 | 1.87 | 63.57 | 0.6 | more than 1.78 | 1 |
| **ENSG00000166847.8** | protein_coding | DCTN5 | 2.64 | 2.55 | 59.55 | 0.61 | more than 2.62 | 1 |
| **ENSG00000167105.6** | protein_coding | TMEM92 | 0.96 | 1.42 | 60.05 | 0.61 | less than 1.00 | 1 |
| **ENSG00000167434.8** | protein_coding | CA4 | 1.88 | 1.31 | 63.32 | 0.63 | more than 1.00 | 1 |
| **ENSG00000168542.11** | protein_coding | COL3A1 | 5.13 | 5.9 | 67.84 | 0.63 | less than 6.10 | 1 |
| **ENSG00000172361.5** | protein_coding | CFAP53 | 1.72 | 1.53 | 64.32 | 0.6 | more than 1.47 | 1 |
| **ENSG00000174348.12** | protein_coding | PODN | 1.01 | 1.37 | 62.81 | 0.62 | less than 1.02 | 1 |
| **ENSG00000176971.3** | protein_coding | FIBIN | 1.33 | 1.68 | 57.79 | 0.61 | less than 1.00 | 1 |
| **ENSG00000179913.9** | protein_coding | B3GNT3 | 2.95 | 3.75 | 56.28 | 0.6 | less than 3.00 | 1 |
| **ENSG00000180447.6** | protein_coding | GAS1 | 1.02 | 1.33 | 64.32 | 0.62 | less than 1.08 | 1 |
| **ENSG00000180626.9** | protein_coding | ZNF594 | 1.39 | 1.26 | 64.07 | 0.6 | more than 1.21 | 1 |
| **ENSG00000182326.13** | protein_coding | C1S | 2.72 | 3.32 | 62.31 | 0.62 | less than 3.14 | 1 |
| **ENSG00000183160.8** | protein_coding | TMEM119 | 1.5 | 1.93 | 66.08 | 0.62 | less than 2.04 | 1 |
| **ENSG00000184517.10** | protein_coding | ZFP1 | 2.29 | 2.15 | 62.81 | 0.62 | more than 2.19 | 1 |
| **ENSG00000186187.10** | protein_coding | ZNRF1 | 2.82 | 2.68 | 67.59 | 0.62 | more than 2.58 | 1 |
| **ENSG00000187244.9** | protein_coding | BCAM | 6 | 5.73 | 63.57 | 0.61 | more than 5.66 | 1 |
| **ENSG00000204634.11** | protein_coding | TBC1D8 | 1.37 | 1.25 | 68.34 | 0.6 | more than 1.09 | 1 |
| **ENSG00000213593.8** | protein_coding | TMX2 | 5.04 | 4.88 | 59.8 | 0.61 | more than 4.97 | 1 |
| **ENSG00000217555.11** | protein_coding | CKLF | 3.08 | 3.44 | 69.1 | 0.61 | less than 3.71 | 1 |
| **ENSG00000232453.4** | lincRNA | RP4-794H19.1 | 1.33 | 1.15 | 57.04 | 0.61 | more than 1.33 | 1 |
| **ENSG00000232470.1** | lincRNA | RP11-313D6.3 | 1.18 | 1.03 | 64.57 | 0.61 | more than 1.00 | 1 |
| **ENSG00000232956.7** | lincRNA | SNHG15 | 1.77 | 1.57 | 68.09 | 0.6 | more than 1.20 | 1 |
| **ENSG00000235109.6** | protein_coding | ZSCAN31 | 1.93 | 1.63 | 62.31 | 0.61 | more than 1.44 | 1 |
| **ENSG00000238243.3** | protein_coding | OR2W3 | 1.63 | 1.26 | 51.51 | 0.6 | more than 2.00 | 1 |
| **ENSG00000242640.1** | processed_pseudogene | RP11-302F12.1 | 2.37 | 3.11 | 60.8 | 0.61 | less than 3.00 | 1 |
| **ENSG00000245573.6** | antisense | BDNF-AS | 1.38 | 1.26 | 62.56 | 0.61 | more than 1.28 | 1 |
| **ENSG00000260314.2** | protein_coding | MRC1 | 1.8 | 2.24 | 62.56 | 0.61 | less than 2.07 | 1 |
| **ENSG00000261786.1** | lincRNA | RP4-555D20.2 | 2.06 | 1.74 | 56.03 | 0.6 | more than 2.10 | 1 |
| **ENSG00000266916.4** | antisense | ZNF793-AS1 | 1.82 | 1.65 | 62.81 | 0.6 | more than 1.63 | 1 |
| **ENSG00000269176.2** | antisense | RP11-727F15.12 | 2 | 1.83 | 60.55 | 0.61 | more than 1.89 | 1 |
| **ENSG00000276180.1** | protein_coding | HIST1H4I | 2.84 | 2.66 | 60.8 | 0.61 | more than 2.68 | 1 |
