# Supplementary material for: Expression based biomarkers and models to classify early and late-stage samples of Papillary Thyroid Carcinoma
Source: PLoS One. 2020 Apr 23;15(4):e0231629. doi: 10.1371/journal.pone.0231629 (PMC7179925; doi:10.1371/journal.pone.0231629)
Supplement: S3 Table — (DOCX) [file pone.0231629.s003.docx]

Table S3 : Oncogenic signatures enriched in the transcripts showing Area under the curve differentiation between early and late stage

| **Term** | **Overlap** | **Adjusted P-value** | **Genes** |
| --- | --- | --- | --- |
| ESC_V6.5_UP_EARLY.V1_DN | 11/172 | 0.00 | *COL1A1;COL3A1;COL1A2;FAP;PLAU;SERPINF1;COL11A1;PCOLCE;AEBP1;FBLN2;LOXL1* |
| ATF2_S_UP.V1_DN | 11/187 | 0.00 | *COMP;SFRP4;TNFAIP6;COL11A1;MYBPH;CHI3L1;ITGBL1;GAS1;FBLN2;GABRD;CCL17* |
| RB_P107_DN.V1_UP | 9/140 | 0.00 | *COL1A1;COL1A2;SRPX2;COL5A1;SERPINF1;TMEM119;PCOLCE;FBLN2;LOXL1* |
| CAHOY_ASTROGLIAL | 6/100 | 0.01 | *COL5A1;OGN;OMD;ITGBL1;AEBP1;WISP2* |
| BMI1_DN.V1_UP | 7/147 | 0.01 | *MFAP5;LRRC15;MMP7;SRPX2;FAP;TNFAIP6;GFPT2* |
| AKT_UP.V1_DN | 7/187 | 0.02 | *SFRP2;C1S;OGN;GAS1;ITGBL1;NR1D1;LIMD1* |
| PRC2_EZH2_UP.V1_DN | 7/194 | 0.02 | *SRPX2;PPP2R1B;TNFAIP6;GFPT2;COL10A1;PAMR1;ASPN* |
| MEL18_DN.V1_UP | 6/141 | 0.02 | *MMP7;SRPX2;TNFAIP6;FAP;GFPT2;VEGFA* |
| BMI1_DN_MEL18_DN.V1_UP | 6/145 | 0.02 | *LRRC15;MMP7;FAP;TNFAIP6;GFPT2;VEGFA* |
