# Supplementary material for: Expression based biomarkers and models to classify early and late-stage samples of Papillary Thyroid Carcinoma
Source: PLoS One. 2020 Apr 23;15(4):e0231629. doi: 10.1371/journal.pone.0231629 (PMC7179925; doi:10.1371/journal.pone.0231629)
Supplement: S4 Table — (DOCX) [file pone.0231629.s004.docx]

Table S4: Performance measures of 179 transcripts set selected by expression threshold based AUROC ranking on training model and independent validation dataset by implementing SVC using Scikit and various other machine-learning algorithms

| **Classifier** | **Dataset** | **TP** | **FP** | **TN** | **FN** | **Recall**  **(%)** | **Precision**  **(%)** | **Spec**  **(%)** | **Accuracy**  **(%)** | **MCC** | **AUROC with 95% CI** | **F1 score** |
| --- | --- | --- | --- | --- | --- | --- | --- | --- | --- | --- | --- | --- |
| SVC | Training | 196 | 53 | 80 | 69 | 73.96 | 78.71 | 60.15 | 69.35 | 0.33 | 0.72(0.67-0.78) | 0.69 |
|  | Validation | 54 | 20 | 14 | 14 | 79.41 | 72.97 | 41.18 | 66.67 | 0.22 | 0.7(0.59-0.82) | 0.67 |
| SMO | Training | 239 | 80 | 53 | 26 | 90.19 | 74.92 | 39.85 | 73.37 | 0.36 | 0.65(0;60-0.70) | 0.73 |
|  | Validation | 62 | 26 | 8 | 6 | 91.18 | 70.45 | 23.53 | 68.63 | 0.2 | 0.57(0.49-0.65) | 0.69 |
| J48 | Training | 82 | 46 | 87 | 83 | 68.68 | 64.06 | 65.41 | 67.59 | 0.33 | 0.68(0.62-0.74) | 0.6 |
|  | Validation | 41 | 20 | 14 | 27 | 60.29 | 67.21 | 41.18 | 53.92 | 0.01 | 0.50(0.38-0.61) | 0.54 |
| NB | Training | 198 | 56 | 77 | 67 | 74.72 | 77.95 | 57.89 | 69.1 | 0.32 | 0.67(0.62-0.72) | 0.69 |
|  | Validation | 56 | 21 | 13 | 12 | 82.35 | 72.73 | 38.24 | 67.65 | 0.23 | 0.64(0.54-0.74) | 0.68 |
| RF | Training | 183 | 48 | 85 | 82 | 69.06 | 79.22 | 63.91 | 67.34 | 0.32 | 0.68(0.63-0.74) | 0.58 |
|  | Validation | 45 | 16 | 18 | 23 | 66.18 | 73.77 | 52.94 | 61.76 | 0.18 | 0.65(0.54-0.77) | 0.62 |
