# Supplementary material for: Expression based biomarkers and models to classify early and late-stage samples of Papillary Thyroid Carcinoma
Source: PLoS One. 2020 Apr 23;15(4):e0231629. doi: 10.1371/journal.pone.0231629 (PMC7179925; doi:10.1371/journal.pone.0231629)
Supplement: S5 Table — (DOCX) [file pone.0231629.s005.docx]

Table S5: List of 100 protein coding transcripts selected using F_ANOVA

| **S No.** | **Transcript ID** | **Gene Symbol** |
| --- | --- | --- |
| 1 | ENSG00000142611.15 | *PRDM16* |
| 2 | ENSG00000117461.13 | *PIK3R3* |
| 3 | ENSG00000060718.17 | *COL11A1* |
| 4 | ENSG00000163207.6 | *IVL* |
| 5 | ENSG00000196154.10 | *S100A4* |
| 6 | ENSG00000163472.17 | *TMEM79* |
| 7 | ENSG00000162746.13 | *FCRLB* |
| 8 | ENSG00000116260.15 | *QSOX1* |
| 9 | ENSG00000159166.12 | *LAD1* |
| 10 | ENSG00000133055.7 | *MYBPH* |
| 11 | ENSG00000186007.8 | *LEMD1* |
| 12 | ENSG00000132031.11 | *MATN3* |
| 13 | ENSG00000168754.12 | *FAM178B* |
| 14 | ENSG00000078098.12 | *FAP* |
| 15 | ENSG00000138399.16 | *FASTKD1* |
| 16 | ENSG00000198919.11 | *DZIP3* |
| 17 | ENSG00000177707.9 | *PVRL3* |
| 18 | ENSG00000114098.16 | *ARMC8* |
| 19 | ENSG00000163762.5 | *TM4SF18* |
| 20 | ENSG00000180044.4 | *C3orf80* |
| 21 | ENSG00000172061.8 | *LRRC15* |
| 22 | ENSG00000178163.6 | *ZNF518B* |
| 23 | ENSG00000109705.7 | *NKX3-2* |
| 24 | ENSG00000163071.9 | *SPATA18* |
| 25 | ENSG00000129128.11 | *SPCS3* |
| 26 | ENSG00000151883.15 | *PARP8* |
| 27 | ENSG00000113621.13 | *TXNDC15* |
| 28 | ENSG00000183876.8 | *ARSI* |
| 29 | ENSG00000204764.11 | *RANBP17* |
| 30 | ENSG00000037280.14 | *FLT4* |
| 31 | ENSG00000172201.9 | *ID4* |
| 32 | ENSG00000124602.8 | *UNC5CL* |
| 33 | ENSG00000112715.19 | *VEGFA* |
| 34 | ENSG00000123500.8 | *COL10A1* |
| 35 | ENSG00000164520.10 | *RAET1E* |
| 36 | ENSG00000181472.4 | *ZBTB2* |
| 37 | ENSG00000080802.17 | *CNOT4* |
| 38 | ENSG00000171115.3 | *GIMAP8* |
| 39 | ENSG00000179144.4 | *GIMAP7* |
| 40 | ENSG00000133561.14 | *GIMAP6* |
| 41 | ENSG00000180543.4 | *TSPYL5* |
| 42 | ENSG00000147654.13 | *EBAG9* |
| 43 | ENSG00000155792.8 | *DEPTOR* |
| 44 | ENSG00000008513.13 | *ST3GAL1* |
| 45 | ENSG00000156049.6 | *GNA14* |
| 46 | ENSG00000106819.10 | *ASPN* |
| 47 | ENSG00000044574.7 | *HSPA5* |
| 48 | ENSG00000167123.17 | *CERCAM* |
| 49 | ENSG00000138316.9 | *ADAMTS14* |
| 50 | ENSG00000095596.10 | *CYP26A1* |
| 51 | ENSG00000203805.9 | *PPAPDC1A* |
| 52 | ENSG00000110066.13 | *SUV420H1* |
| 53 | ENSG00000110075.13 | *PPP6R3* |
| 54 | ENSG00000175536.6 | *LIPT2* |
| 55 | ENSG00000118363.10 | *SPCS2* |
| 56 | ENSG00000137745.10 | *MMP13* |
| 57 | ENSG00000137713.14 | *PPP2R1B* |
| 58 | ENSG00000150433.8 | *TMEM218* |
| 59 | ENSG00000197614.9 | *MFAP5* |
| 60 | ENSG00000185432.11 | *METTL7A* |
| 61 | ENSG00000166598.11 | *HSP90B1* |
| 62 | ENSG00000150977.10 | *RILPL2* |
| 63 | ENSG00000178882.12 | *FAM101A* |
| 64 | ENSG00000133121.19 | *STARD13* |
| 65 | ENSG00000102738.7 | *MRPS31* |
| 66 | ENSG00000102543.13 | *CDADC1* |
| 67 | ENSG00000102580.13 | *DNAJC3* |
| 68 | ENSG00000198542.12 | *ITGBL1* |
| 69 | ENSG00000053770.10 | *AP5M1* |
| 70 | ENSG00000133961.18 | *NUMB* |
| 71 | ENSG00000172345.12 | *STARD5* |
| 72 | ENSG00000102970.9 | *CCL17* |
| 73 | ENSG00000141098.11 | *GFOD2* |
| 74 | ENSG00000141084.9 | *RANBP10* |
| 75 | ENSG00000166455.12 | *C16orf46* |
| 76 | ENSG00000184860.8 | *SDR42E1* |
| 77 | ENSG00000230989.5 | *HSBP1* |
| 78 | ENSG00000167874.6 | *TMEM88* |
| 79 | ENSG00000006695.9 | *COX10* |
| 80 | ENSG00000198863.6 | *RUNDC1* |
| 81 | ENSG00000108828.14 | *VAT1* |
| 82 | ENSG00000153822.12 | *KCNJ16* |
| 83 | ENSG00000141219.14 | *C17orf80* |
| 84 | ENSG00000176170.12 | *SPHK1* |
| 85 | ENSG00000177576.9 | *C18orf32* |
| 86 | ENSG00000171791.11 | *BCL2* |
| 87 | ENSG00000166396.11 | *SERPINB7* |
| 88 | ENSG00000176533.11 | *GNG7* |
| 89 | ENSG00000104918.6 | *RETN* |
| 90 | ENSG00000132000.10 | *PODNL1* |
| 91 | ENSG00000105483.15 | *CARD8* |
| 92 | ENSG00000104808.6 | *DHDH* |
| 93 | ENSG00000179954.13 | *SSC5D* |
| 94 | ENSG00000078804.11 | *TP53INP2* |
| 95 | ENSG00000064205.9 | *WISP2* |
| 96 | ENSG00000182162.8 | *P2RY8* |
| 97 | ENSG00000124343.11 | *XG* |
| 98 | ENSG00000078596.9 | *ITM2A* |
| 99 | ENSG00000102359.5 | *SRPX2* |
| 100 | ENSG00000102385.11 | *DRP2* |
