# Supplementary material for: Expression based biomarkers and models to classify early and late-stage samples of Papillary Thyroid Carcinoma
Source: PLoS One. 2020 Apr 23;15(4):e0231629. doi: 10.1371/journal.pone.0231629 (PMC7179925; doi:10.1371/journal.pone.0231629)
Supplement: S6 Table — (DOCX) [file pone.0231629.s006.docx]

Table S6: Performance measures of 100 protein coding mRNA feature set (THCA-EL-F-PC) selected by F_ANOVA method on training model and independent validation dataset by implementing various machine-learning algorithms

| **Classifier** | **Dataset** | **TP** | **FP** | **TN** | **FN** | **Recall**  **(%)** | **Precision**  **(%)** | **Spec**  **(%)** | **Acc**  **(%)** | **MCC** | **AUROC with 95% CI** | **F1 score** |
| --- | --- | --- | --- | --- | --- | --- | --- | --- | --- | --- | --- | --- |
| **SVC** | Training | 202 | 52 | 81 | 63 | 76.23 | 79.53 | 60.9 | 71.11 | 0.36 | 0.73(0.68-0.79) | 0.71 |
|  | Validation | 45 | 10 | 24 | 23 | 66.18 | 81.82 | 70.59 | 67.65 | 0.35 | 0.71(0.60-0.82) | 0.68 |
| **SMO** | Training | 250 | 83 | 50 | 15 | 94.34 | 75.08 | 37.59 | 75.38 | 0.41 | 0.66(0.61-0.70) | 0.75 |
|  | Validation | 60 | 23 | 11 | 8 | 88.24 | 72.29 | 32.35 | 69.61 | 0.25 | 0.60(0.59-0.69) | 0.7 |
| **J48** | Training | 215 | 83 | 50 | 50 | 81.13 | 72.15 | 37.59 | 66.58 | 0.2 | 0.59(0.53-0.65) | 0.66 |
|  | Validation | 51 | 19 | 15 | 17 | 75 | 72.86 | 44.12 | 64.71 | 0.19 | 0.59(0.47-0.71) | 0.65 |
| **NB** | Training | 202 | 61 | 72 | 63 | 76.23 | 76.81 | 54.14 | 68.84 | 0.30 | 0.66(0.61-0.71) | 0.69 |
|  | Validation | 49 | 11 | 23 | 19 | 72.06 | 81.67 | 67.65 | 70.59 | 0.38 | 0.69(0.58-0.79) | 0.71 |
| **RF** | Training | 184 | 53 | 80 | 81 | 69.43 | 77.64 | 60.15 | 66.33 | 0.28 | 0.71(0.65-0.76) | 0.58 |
|  | Validation | 32 | 8 | 26 | 36 | 47.06 | 80.00 | 76.47 | 56.86 | 0.23 | 0.67(0.55-0.79) | 0.57 |
