# Supplementary material for: Expression based biomarkers and models to classify early and late-stage samples of Papillary Thyroid Carcinoma
Source: PLoS One. 2020 Apr 23;15(4):e0231629. doi: 10.1371/journal.pone.0231629 (PMC7179925; doi:10.1371/journal.pone.0231629)
Supplement: S7 Table — (DOCX) [file pone.0231629.s007.docx]

Table S7: Performance of SVC based models and WEKA based models on the hallmark transcripts selected by WEKA (THCA-EL-H) on training dataset and independent validation dataset

| **Classifier** | **Dataset** | **TP** | **FP** | **TN** | **FN** | **Recall**  **(%)** | **Precision**  **(%)** | **Spec**  **(%)** | **Accuracy**  **(%)** | **MCC** | **AUROC with 95% CI** | **F1 score** |
| --- | --- | --- | --- | --- | --- | --- | --- | --- | --- | --- | --- | --- |
| **SVC** | Training | 180 | 42 | 91 | 85 | 67.92 | 81.08 | 68.42 | 68.09 | 0.35 | 0.71  (0.66-0.77 | 0.68 |
|  | Validation | 47 | 11 | 23 | 21 | 69.12 | 81.03 | 67.65 | 68.63 | 0.35 | 0.73  (0.61-0.85) | 0.69 |
| **SMO** | Training | 235 | 83 | 50 | 30 | 88.68 | 73.90 | 37.59 | 71.61 | 0.31 | 0.63  (0.59-0.68) | 0.72 |
|  | Validation | 60 | 18 | 16 | 8 | 88.24 | 76.92 | 47.06 | 74.51 | 0.39 | 0.68  (0.58-0.77) | 0.75 |
| **J48** | Training | 173 | 44 | 89 | 92 | 65.28 | 79.72 | 66.92 | 65.83 | 0.31 | 0.69  (0.630.74) | 0.65 |
|  | Validation | 44 | 19 | 15 | 24 | 64.71 | 69.84 | 44.12 | 57.84 | 0.09 | 0.50  (0.38-0.68) | 0.58 |
| **NB** | Training | 173 | 45 | 88 | 92 | 65.28 | 79.36 | 66.17 | 65.58 | 0.3 | 0.71  (0.66-0.77) | 0.66 |
|  | Validation | 36 | 12 | 22 | 32 | 52.94 | 75.00 | 64.71 | 56.86 | 0.17 | 0.66  (0.54-0.79) | 0.57 |
| **RF** | Training | 211 | 53 | 80 | 54 | 79.62 | 79.92 | 60.15 | 73.12 | 0.4 | 0.75  (0.70-0.80) | 0.72 |
|  | Validation | 50 | 14 | 20 | 18 | 73.53 | 78.13 | 58.82 | 68.63 | 0.32 | 0.72  (0.61-0.82) | 0.66 |
