# Supplementary material for: Expression based biomarkers and models to classify early and late-stage samples of Papillary Thyroid Carcinoma
Source: PLoS One. 2020 Apr 23;15(4):e0231629. doi: 10.1371/journal.pone.0231629 (PMC7179925; doi:10.1371/journal.pone.0231629)
Supplement: S8 Table — (DOCX) [file pone.0231629.s008.docx]

Table S8: Performance measures of 50 hallmark protein transcripts (THCA-EL-FH) feature set selected by F_ANOVA method on training model and independent validation dataset by implementing SVC using Scikit and various other machine-learning algorithms using WEKA

| **Classifier** | **Dataset** | **TP** | **FP** | **TN** | **FN** | **Recall**  **(%)** | **Precision**  **(%)** | **Spec**  **(%)** | **Accuracy**  **(%)** | **MCC** | **AUROC with 95% CI** | **F1 score** |
| --- | --- | --- | --- | --- | --- | --- | --- | --- | --- | --- | --- | --- |
| **SVC** | Training | 193 | 53 | 80 | 72 | 72.83 | 78.46 | 60.15 | 68.59 | 0.32 | 0.69(0.63-0.74) | 0.69 |
|  | Validation | 44 | 13 | 21 | 24 | 64.71 | 77.19 | 61.76 | 63.73 | 0.25 | 0.68(0.56-0.79) | 0.64 |
| **SMO** | Training | 248 | 90 | 43 | 17 | 93.58 | 73.37 | 32.33 | 73.12 | 0.34 | 0.62(0.59-0.67) | 0.73 |
|  | Validation | 61 | 22 | 12 | 7 | 89.71 | 73.49 | 35.29 | 71.57 | 0.3 | 0.62(0.54-0.71) | 0.72 |
| **J48** | Training | 229 | 99 | 34 | 36 | 86.42 | 69.82 | 25.56 | 66.08 | 0.15 | 0.53(0.46-0.59) | 0.66 |
|  | Validation | 53 | 16 | 18 | 15 | 77.94 | 76.81 | 52.94 | 69.61 | 0.31 | 0.69(0.58-0.79) | 0.7 |
| **NB** | Training | 188 | 57 | 76 | 77 | 70.94 | 76.73 | 57.14 | 66.33 | 0.27 | 0.67(0.61-0.72) | 0.66 |
|  | Validation | 42 | 13 | 21 | 26 | 61.76 | 76.36 | 61.76 | 61.76 | 0.22 | 0.65(0.54-0.75) | 0.62 |
| **RF** | Training | 170 | 46 | 87 | 95 | 64.15 | 78.70 | 65.41 | 64.57 | 0.28 | 0.68(0.62-0.74) | 0.61 |
|  | Validation | 37 | 13 | 21 | 31 | 54.41 | 74.00 | 61.76 | 56.86 | 0.15 | 0.65(0.53-0.77) | 0.55 |
