# Supplementary material for: Expression based biomarkers and models to classify early and late-stage samples of Papillary Thyroid Carcinoma
Source: PLoS One. 2020 Apr 23;15(4):e0231629. doi: 10.1371/journal.pone.0231629 (PMC7179925; doi:10.1371/journal.pone.0231629)
Supplement: S9 Table — (DOCX) [file pone.0231629.s009.docx]

Table S9: Performance measures of 78-full feature set (THA-EL-All-WEKA) selected by FCBF-WEKA on training model and independent validation dataset by implementing various machine-learning algorithms.

| **Classifier** | **Dataset** | **TP** | **FP** | **TN** | **FN** | **Recall**  **(%)** | **Precision**  **(%)** | **Spec**  **(%)** | **Accuracy**  **(%)** | **MCC** | **AUROC with 95% CI** | **F1 score** |
| --- | --- | --- | --- | --- | --- | --- | --- | --- | --- | --- | --- | --- |
| **SVC** | Training | 207 | 29 | 104 | 58 | 78.11 | 87.71 | 78.2 | 78.14 | 0.54 | 0.86(0.83-0.90) | 0.78 |
|  | Validation | 49 | 12 | 22 | 19 | 72.06 | 80.33 | 64.71 | 69.61 | 0.35 | 0.73(0.63-0.84) | 0.70 |
| **SMO** | Training | 257 | 65 | 68 | 8 | 96.98 | 79.81 | 51.13 | 81.66 | 0.58 | 0.52(0.50-0.54) | 0.82 |
|  | Validation | 63 | 23 | 11 | 5 | 92.65 | 73.26 | 32.35 | 72.55 | 0.32 | 0.74(0.70-0.78) | 0.73 |
| **J48** | Training | 199 | 38 | 95 | 66 | 75.09 | 83.97 | 71.43 | 73.87 | 0.45 | 0.74(0.69-0.79) | 0.74 |
|  | Validation | 134 | 64 | 4 | 4 | 97.06 | 67.68 | 5.88 | 66.67 | 0.07 | 0.51(0.47-0.54) | 0.64 |
| **NB** | Training | 187 | 25 | 108 | 78 | 70.57 | 88.21 | 81.2 | 74.12 | 0.49 | 0.81(0.77-0.86) | 0.74 |
|  | Validation | 46 | 13 | 21 | 22 | 67.65 | 77.97 | 61.76 | 65.69 | 0.28 | 0.68(0.57-0.79) | 0.66 |
| **RF** | Training | 204 | 36 | 97 | 61 | 76.98 | 85.00 | 72.93 | 75.63 | 0.48 | 0.81(0.76-0.86) | 0.65 |
|  | Validation | 45 | 13 | 21 | 23 | 66.18 | 77.59 | 61.76 | 64.71 | 0.27 | 0.67(0.56-0.79) | 0.62 |
