# Supplementary material for: Expression based biomarkers and models to classify early and late-stage samples of Papillary Thyroid Carcinoma
Source: PLoS One. 2020 Apr 23;15(4):e0231629. doi: 10.1371/journal.pone.0231629 (PMC7179925; doi:10.1371/journal.pone.0231629)
Supplement: S10 Table — (DOCX) [file pone.0231629.s010.docx]

Table S10: Features selected from WEKA based feature selection using all types of transcripts (THCA-EL-All)

| **Transcript ID** | **Mean in Early** | **Mean in Late** | **Type of transcript** | **Gene Symbol** |
| --- | --- | --- | --- | --- |
| ENSG00000254608.1 | 0.11 | 0.22 | unprocessed_pseudogene | *RP11-56A10.1* |
| ENSG00000229031.1 | 0.02 | 0.01 | unprocessed_pseudogene | *RP11-666G4.1* |
| ENSG00000214198.6 | 0.29 | 0.22 | unitary_pseudogene | *RP11-642P15.1* |
| ENSG00000214856.10 | 0.02 | 0.02 | transcribed_unprocessed_pseudogene | *KRT16P1* |
| ENSG00000224287.2 | 0.20 | 0.14 | transcribed_processed_pseudogene | *MSL3P1* |
| ENSG00000279420.1 | 0.01 | 0.00 | TEC | *RP11-42H13.1* |
| ENSG00000274607.1 | 0.05 | 0.03 | snRNA | *U6* |
| ENSG00000248285.1 | 0.06 | 0.03 | sense_overlapping | *RP11-158J3.2* |
| ENSG00000277386.1 | 0.42 | 0.27 | sense_intronic | *RP11-141M1.4* |
| ENSG00000232031.1 | 0.03 | 0.01 | sense_intronic | *RP5-991C6.4* |
| ENSG00000279336.1 | 0.03 | 0.01 | pseudogene | *AL353662.2* |
| ENSG00000164362.17 | 0.01 | 0.05 | protein_coding | *TERT* |
| ENSG00000128040.9 | 1.41 | 1.77 | protein_coding | *SPINK2* |
| ENSG00000037280.14 | 2.25 | 1.79 | protein_coding | *FLT4* |
| ENSG00000139318.7 | 6.15 | 6.43 | protein_coding | *DUSP6* |
| ENSG00000108219.13 | 2.76 | 2.61 | protein_coding | *TSPAN14* |
| ENSG00000188763.4 | 0.14 | 0.07 | protein_coding | *FZD9* |
| ENSG00000169895.5 | 3.55 | 3.45 | protein_coding | *SYAP1* |
| ENSG00000139679.14 | 3.22 | 3.14 | protein_coding | *LPAR6* |
| ENSG00000164398.11 | 0.09 | 0.06 | protein_coding | *ACSL6* |
| ENSG00000089169.13 | 0.06 | 0.04 | protein_coding | *RPH3A* |
| ENSG00000047648.20 | 2.39 | 2.15 | protein_coding | *ARHGAP6* |
| ENSG00000174469.16 | 0.20 | 0.12 | protein_coding | *CNTNAP2* |
| ENSG00000129968.14 | 2.56 | 2.52 | protein_coding | *ABHD17A* |
| ENSG00000178662.14 | 1.02 | 0.92 | protein_coding | *CSRNP3* |
| ENSG00000101844.16 | 2.91 | 2.78 | protein_coding | *ATG4A* |
| ENSG00000119919.10 | 0.32 | 0.20 | protein_coding | *NKX2-3* |
| ENSG00000042813.6 | 0.06 | 0.04 | protein_coding | *ZPBP* |
| ENSG00000073803.12 | 2.26 | 2.20 | protein_coding | *MAP3K13* |
| ENSG00000103194.14 | 3.83 | 3.78 | protein_coding | *USP10* |
| ENSG00000213934.5 | 0.01 | 0.00 | protein_coding | *HBG1* |
| ENSG00000171960.9 | 3.80 | 3.83 | protein_coding | *PPIH* |
| ENSG00000113456.17 | 2.19 | 2.28 | protein_coding | *RAD1* |
| ENSG00000107821.13 | 1.12 | 1.31 | protein_coding | *KAZALD1* |
| ENSG00000116035.2 | 0.18 | 0.21 | protein_coding | *VAX2* |
| ENSG00000196787.3 | 0.70 | 0.57 | protein_coding | *HIST1H2AG* |
| ENSG00000115138.9 | 0.83 | 0.56 | protein_coding | *POMC* |
| ENSG00000123576.5 | 0.00 | 0.00 | protein_coding | *ESX1* |
| ENSG00000173702.6 | 0.02 | 0.03 | protein_coding | *MUC13* |
| ENSG00000174912.7 | 0.61 | 0.55 | processed_pseudogene | *METTL15P1* |
| ENSG00000231715.1 | 0.08 | 0.12 | processed_pseudogene | *COX6CP2* |
| ENSG00000253884.1 | 0.13 | 0.30 | processed_pseudogene | *RP11-726G23.2* |
| ENSG00000253908.1 | 0.07 | 0.06 | processed_pseudogene | *RP11-252I14.2* |
| ENSG00000269001.1 | 1.75 | 1.70 | processed_pseudogene | *ZNF818P* |
| ENSG00000214298.3 | 0.04 | 0.02 | processed_pseudogene | *MRPS21P6* |
| ENSG00000271137.1 | 0.01 | 0.01 | processed_pseudogene | *RP5-966M1.4* |
| ENSG00000224725.3 | 0.02 | 0.01 | processed_pseudogene | *CEP57L1P1* |
| ENSG00000231404.2 | 0.01 | 0.01 | processed_pseudogene | *RAC1P3* |
| ENSG00000259493.2 | 0.02 | 0.01 | processed_pseudogene | *RP11-621H8.2* |
| ENSG00000250787.1 | 0.16 | 0.11 | processed_pseudogene | *HMGN1P17* |
| ENSG00000239279.3 | 0.05 | 0.02 | misc_RNA | *RN7SL184P* |
| ENSG00000266463.1 | 0.05 | 0.18 | miRNA | *MIR3196* |
| ENSG00000274115.1 | 0.09 | 0.04 | miRNA | *MIR6081* |
| ENSG00000236385.1 | 0.03 | 0.09 | lincRNA | *RP11-114M1.2* |
| ENSG00000233393.1 | 0.16 | 0.06 | lincRNA | *AP000688.29* |
| ENSG00000258976.1 | 0.25 | 0.29 | lincRNA | *CTD-2207P18.2* |
| ENSG00000272264.1 | 0.41 | 0.42 | lincRNA | *RP11-92K15.3* |
| ENSG00000236106.1 | 0.00 | 0.01 | lincRNA | *AC010729.2* |
| ENSG00000259434.1 | 0.01 | 0.00 | lincRNA | *RP11-720L8.1* |
| ENSG00000261325.1 | 0.00 | 0.00 | lincRNA | *AC140542.2* |
| ENSG00000251273.1 | 0.04 | 0.02 | lincRNA | *RP11-549K20.1* |
| ENSG00000228826.2 | 0.02 | 0.00 | lincRNA | *RP11-344P13.4* |
| ENSG00000261512.2 | 1.65 | 1.53 | lincRNA | *RP11-46D6.1* |
| ENSG00000281904.1 | 0.01 | 0.01 | lincRNA | *CH17-132F21.5* |
| ENSG00000266869.1 | 0.06 | 0.04 | lincRNA | *RP6-114E22.1* |
| ENSG00000211972.2 | 1.43 | 1.04 | IG_V_gene | *IGHV3-66* |
| ENSG00000227673.1 | 0.02 | 0.04 | antisense | *RP11-243J18.2* |
| ENSG00000246174.6 | 0.75 | 0.65 | antisense | *KCTD21-AS1* |
| ENSG00000272975.1 | 0.00 | 0.01 | antisense | *MYHAS* |
| ENSG00000253616.4 | 0.22 | 0.30 | antisense | *RP11-875O11.3* |
| ENSG00000198711.5 | 0.14 | 0.12 | antisense | *SSBP3-AS1* |
| ENSG00000233191.1 | 0.04 | 0.02 | antisense | *AC006372.6* |
| ENSG00000235875.3 | 0.03 | 0.02 | antisense | *ARHGEF7-AS2* |
| ENSG00000259221.4 | 0.02 | 0.01 | antisense | *CTD-2050N2.1* |
| ENSG00000249307.4 | 0.07 | 0.10 | antisense | *LINC01088* |
| ENSG00000254400.1 | 0.02 | 0.01 | antisense | *RP11-732A19.8* |
| ENSG00000255084.1 | 0.04 | 0.02 | antisense | *RP11-843A23.1* |
| ENSG00000253596.1 | 0.05 | 0.03 | antisense | *CTD-2320G14.2* |
