# Supplementary material for: Expression based biomarkers and models to classify early and late-stage samples of Papillary Thyroid Carcinoma
Source: PLoS One. 2020 Apr 23;15(4):e0231629. doi: 10.1371/journal.pone.0231629 (PMC7179925; doi:10.1371/journal.pone.0231629)
Supplement: S11 Table — (DOCX) [file pone.0231629.s011.docx]

Table S11: The 100 F_ANOVA features selected from all types of transcripts (THCA-EL-All-F)

| **Transcript ID** | **Transcript Type** | **Gene Symbol** |
| --- | --- | --- |
| ENSG00000118363.10 | protein_coding | *SPCS2* |
| ENSG00000168300.12 | protein_coding | *PCMTD1* |
| ENSG00000109705.7 | protein_coding | *NKX3-2* |
| ENSG00000117461.13 | protein_coding | *PIK3R3* |
| ENSG00000198863.6 | protein_coding | *RUNDC1* |
| ENSG00000169359.12 | protein_coding | *SLC33A1* |
| ENSG00000163207.6 | protein_coding | *IVL* |
| ENSG00000104918.6 | protein_coding | *RETN* |
| ENSG00000006695.9 | protein_coding | *COX10* |
| ENSG00000108828.14 | protein_coding | *VAT1* |
| ENSG00000124343.11 | protein_coding | *XG* |
| ENSG00000254343.2 | lincRNA | *RP11-760H22.2* |
| ENSG00000271147.6 | processed_transcript | *RP4-769N13.6* |
| ENSG00000178882.12 | protein_coding | *FAM101A* |
| ENSG00000272970.1 | lincRNA | *RP11-329B9.4* |
| ENSG00000171115.3 | protein_coding | *GIMAP8* |
| ENSG00000185432.11 | protein_coding | *METTL7A* |
| ENSG00000112715.19 | protein_coding | *VEGFA* |
| ENSG00000182162.8 | protein_coding | *P2RY8* |
| ENSG00000008513.13 | protein_coding | *ST3GAL1* |
| ENSG00000153989.7 | protein_coding | *NUS1* |
| ENSG00000071243.14 | protein_coding | *ING3* |
| ENSG00000181472.4 | protein_coding | *ZBTB2* |
| ENSG00000186007.8 | protein_coding | *LEMD1* |
| ENSG00000261295.1 | antisense | *RP11-524D16__A.3* |
| ENSG00000167842.14 | protein_coding | *MIS12* |
| ENSG00000247934.4 | antisense | *RP11-967K21.1* |
| ENSG00000110066.13 | protein_coding | *SUV420H1* |
| ENSG00000141219.14 | protein_coding | *C17orf80* |
| ENSG00000147642.15 | protein_coding | *SYBU* |
| ENSG00000179954.13 | protein_coding | *SSC5D* |
| ENSG00000130449.5 | protein_coding | *ZSWIM6* |
| ENSG00000037280.14 | protein_coding | *FLT4* |
| ENSG00000102359.5 | protein_coding | *SRPX2* |
| ENSG00000151883.15 | protein_coding | *PARP8* |
| ENSG00000172061.8 | protein_coding | *LRRC15* |
| ENSG00000114098.16 | protein_coding | *ARMC8* |
| ENSG00000229692.3 | sense_intronic | *SOS1-IT1* |
| ENSG00000257267.2 | unitary_pseudogene | *ZNF271P* |
| ENSG00000159166.12 | protein_coding | *LAD1* |
| ENSG00000183876.8 | protein_coding | *ARSI* |
| ENSG00000060718.17 | protein_coding | *COL11A1* |
| ENSG00000232679.1 | lincRNA | *RP11-400N13.3* |
| ENSG00000129128.11 | protein_coding | *SPCS3* |
| ENSG00000132000.10 | protein_coding | *PODNL1* |
| ENSG00000174839.11 | protein_coding | *DENND6A* |
| ENSG00000255364.1 | lincRNA | *RP11-94A24.1* |
| ENSG00000133055.7 | protein_coding | *MYBPH* |
| ENSG00000236385.1 | lincRNA | *RP11-114M1.2* |
| ENSG00000136161.11 | protein_coding | *RCBTB2* |
| ENSG00000203805.9 | protein_coding | *PPAPDC1A* |
| ENSG00000163686.12 | protein_coding | *ABHD6* |
| ENSG00000102760.12 | protein_coding | *RGCC* |
| ENSG00000078098.12 | protein_coding | *FAP* |
| ENSG00000135241.15 | protein_coding | *PNPLA8* |
| ENSG00000254142.2 | lincRNA | *RP11-53M11.3* |
| ENSG00000178163.6 | protein_coding | *ZNF518B* |
| ENSG00000137745.10 | protein_coding | *MMP13* |
| ENSG00000163527.8 | protein_coding | *STT3B* |
| ENSG00000180543.4 | protein_coding | *TSPYL5* |
| ENSG00000108389.8 | protein_coding | *MTMR4* |
| ENSG00000171791.11 | protein_coding | *BCL2* |
| ENSG00000163788.12 | protein_coding | *SNRK* |
| ENSG00000133121.19 | protein_coding | *STARD13* |
| ENSG00000077254.13 | protein_coding | *USP33* |
| ENSG00000164219.8 | protein_coding | *PGGT1B* |
| ENSG00000078596.9 | protein_coding | *ITM2A* |
| ENSG00000147654.13 | protein_coding | *EBAG9* |
| ENSG00000064205.9 | protein_coding | *WISP2* |
| ENSG00000182253.13 | protein_coding | *SYNM* |
| ENSG00000150433.8 | protein_coding | *TMEM218* |
| ENSG00000078804.11 | protein_coding | *TP53INP2* |
| ENSG00000172671.18 | protein_coding | *ZFAND4* |
| ENSG00000168754.12 | protein_coding | *FAM178B* |
| ENSG00000124602.8 | protein_coding | *UNC5CL* |
| ENSG00000174749.5 | protein_coding | *C4orf32* |
| ENSG00000183688.4 | protein_coding | *FAM101B* |
| ENSG00000123500.8 | protein_coding | *COL10A1* |
| ENSG00000230838.1 | lincRNA | *AC093850.2* |
| ENSG00000135070.12 | protein_coding | *ISCA1* |
| ENSG00000104808.6 | protein_coding | *DHDH* |
| ENSG00000133561.14 | protein_coding | *GIMAP6* |
| ENSG00000137713.14 | protein_coding | *PPP2R1B* |
| ENSG00000053770.10 | protein_coding | *AP5M1* |
| ENSG00000176170.12 | protein_coding | *SPHK1* |
| ENSG00000155792.8 | protein_coding | *DEPTOR* |
| ENSG00000120833.12 | protein_coding | *SOCS2* |
| ENSG00000102580.13 | protein_coding | *DNAJC3* |
| ENSG00000134443.8 | protein_coding | *GRP* |
| ENSG00000163762.5 | protein_coding | *TM4SF18* |
| ENSG00000196911.8 | protein_coding | *KPNA5* |
| ENSG00000138399.16 | protein_coding | *FASTKD1* |
| ENSG00000099377.12 | protein_coding | *HSD3B7* |
| ENSG00000170011.12 | protein_coding | *MYRIP* |
| ENSG00000102970.9 | protein_coding | *CCL17* |
| ENSG00000095596.10 | protein_coding | *CYP26A1* |
| ENSG00000110075.13 | protein_coding | *PPP6R3* |
| ENSG00000198919.11 | protein_coding | *DZIP3* |
| ENSG00000261327.4 | lincRNA | *RP11-863P13.3* |
| ENSG00000243742.4 | transcribed_processed_pseudogene | *RPLP0P2* |
