# Supplementary material for: Expression based biomarkers and models to classify early and late-stage samples of Papillary Thyroid Carcinoma
Source: PLoS One. 2020 Apr 23;15(4):e0231629. doi: 10.1371/journal.pone.0231629 (PMC7179925; doi:10.1371/journal.pone.0231629)
Supplement: S12 Table — (DOCX) [file pone.0231629.s012.docx]

Table S12: Performance measures of 100 (THCA-EL-All-F) all transcripts set selected by F_ANOVA method on training model and independent validation dataset by implementing SVC using Scikit and various other machine-learning algorithms using WEKA

| **Classifier** | **Dataset** | **TP** | **FP** | **TN** | **FN** | **Recall**  **(%)** | **Precision**  **(%)** | **Spec**  **(%)** | **Acc**  **(%)** | **MCC** | **AUROC with 95% CI** | **F1 score** |
| --- | --- | --- | --- | --- | --- | --- | --- | --- | --- | --- | --- | --- |
| **SVC** | Training | 203 | 50 | 83 | 62 | 76.6 | 80.24 | 62.41 | 71.86 | 0.38 | 0.71  (0.66-0.77) | 0.72 |
|  | Validation | 47 | 17 | 17 | 21 | 69.12 | 73.44 | 50 | 62.75 | 0.19 | 0.68  (0.56-0.79) | 0.63 |
| **SMO** | Training | 244 | 78 | 55 | 21 | 92.08 | 75.78 | 41.35 | 75.13 | 0.4 | 0.67  (0.62-0.71) | 0.75 |
|  | Validation | 66 | 24 | 10 | 2 | 97.06 | 73.33 | 29.41 | 74.51 | 0.39 | 0.63  (0.55-0.71) | 0.75 |
| **J48** | Training | 211 | 71 | 62 | 54 | 79.62 | 74.82 | 46.62 | 68.59 | 0.27 | 0.62  (0.58-0.68) | 0.69 |
|  | Validation | 48 | 19 | 15 | 20 | 70.59 | 71.64 | 44.12 | 61.76 | 0.15 | 0.60  (0.48-0.73) | 0.62 |
| **NB** | Training | 208 | 53 | 80 | 57 | 78.49 | 79.69 | 60.15 | 72.36 | 0.38 | 0.70  (0.65-0.76) | 0.72 |
|  | Validation | 47 | 19 | 15 | 21 | 69.12 | 71.21 | 44.12 | 60.78 | 0.13 | 0.55  (0.44-0.66) | 0.61 |
| **RF** | Training | 176 | 52 | 81 | 89 | 66.42 | 77.19 | 60.9 | 64.57 | 0.26 | 0.70  (0.64-0.76) | 0.59 |
|  | Validation | 35 | 8 | 26 | 33 | 51.47 | 81.40 | 76.47 | 59.8 | 0.27 | 0.67  (0.55-0.79) | 0.58 |
