# Supplementary material for: Expression based biomarkers and models to classify early and late-stage samples of Papillary Thyroid Carcinoma
Source: PLoS One. 2020 Apr 23;15(4):e0231629. doi: 10.1371/journal.pone.0231629 (PMC7179925; doi:10.1371/journal.pone.0231629)
Supplement: S13 Table — (DOCX) [file pone.0231629.s013.docx]

Table S13: 27 transcripts (THCA-EL-CODO) selected using F_ANOVA at first step and a wrapper based approach combining human opinion dynamics optimizer and SVC at second steps

| **Transcript ID** | **Transcript Type** | **Gene Symbol** |
| --- | --- | --- |
| ENSG00000064601.15 | protein_coding | *CTSA* |
| ENSG00000118363.10 | protein_coding | *SPCS2* |
| ENSG00000237424.1 | antisense | *FOXD2-AS1* |
| ENSG00000102007.9 | protein_coding | *PLP2* |
| ENSG00000132031.11 | protein_coding | *MATN3* |
| ENSG00000142552.6 | protein_coding | *RCN3* |
| ENSG00000166140.16 | protein_coding | *ZFYVE19* |
| ENSG00000258227.5 | protein_coding | *CLEC5A* |
| ENSG00000109705.7 | protein_coding | *NKX3-2* |
| ENSG00000164932.11 | protein_coding | *CTHRC1* |
| ENSG00000197956.8 | protein_coding | *S100A6* |
| ENSG00000198863.6 | protein_coding | *RUNDC1* |
| ENSG00000106333.11 | protein_coding | *PCOLCE* |
| ENSG00000113555.5 | protein_coding | *PCDH12* |
| ENSG00000112837.15 | protein_coding | *TBX18* |
| ENSG00000175536.6 | protein_coding | *LIPT2* |
| ENSG00000163207.6 | protein_coding | *IVL* |
| ENSG00000006695.9 | protein_coding | *COX10* |
| ENSG00000108828.14 | protein_coding | *VAT1* |
| ENSG00000180044.4 | protein_coding | *C3orf80* |
| ENSG00000124343.11 | protein_coding | *XG* |
| ENSG00000088002.10 | protein_coding | *SULT2B1* |
| ENSG00000271147.6 | processed_transcript | *RP4-769N13.6* |
| ENSG00000178882.12 | protein_coding | *FAM101A* |
| ENSG00000272970.1 | lincRNA | *RP11-329B9.4* |
| ENSG00000171115.3 | protein_coding | *GIMAP8* |
| ENSG00000008513.13 | protein_coding | *ST3GAL1* |
