# Supplementary material for: Expression based biomarkers and models to classify early and late-stage samples of Papillary Thyroid Carcinoma
Source: PLoS One. 2020 Apr 23;15(4):e0231629. doi: 10.1371/journal.pone.0231629 (PMC7179925; doi:10.1371/journal.pone.0231629)
Supplement: S14 Table — (DOCX) [file pone.0231629.s014.docx]

Table S14: Performance measures of 27-full features (THCA-EL-CODO) set selected by human opinion dynamics wrapper approach on training model and independent validation dataset

| **Technique** | **Training** | | | | | | **Validation** | | | | | |
| --- | --- | --- | --- | --- | --- | --- | --- | --- | --- | --- | --- | --- |
|  | **Sens**  **(%)** | **Spec**  **(%)** | **Acc**  **(%)** | **MCC** | **F1-score** | **AUROC with 95% CI** | **Sens**  **(%)** | **Spec**  **(%)** | **Acc**  **(%)** | **MCC** | **F1-score** | **AUROC**  **with 95% CI** |
| **SVC** | 59.56 | 77.46 | 71.49 | 0.37 | 0.59 | 0.69  (0.63-0.74) | 69.7 | 65.67 | 67 | 0.33 | 0.58 | 0.68  (0.56-0.79) |
| **NB** | 72.3 | 56.8 | 72.25 | 0.33 | 0.70 | 0.72  (0.67-0.78) | 72 | 61.1 | 72 | 0.34 | 0.72 | 0.73  (0.62-0.84) |
| **RF** | 70.8 | 61.9 | 70.75 | 0.33 | 0.71 | 0.7  (0.64-0.76) | 69 | 63.2 | 69 | 0.32 | 0.69 | 0.7  0.59-0.82) |
| **SMO** | 71.8 | 64.7 | 71.75 | 0.37 | 0.72 | 0.68  (0.62-0.74) | 70 | 62.2 | 70 | 0.32 | 0.70 | 0.66  (0.54-0.78) |
| **j48** | 63.8 | 58.4 | 63.75 | 0.22 | 0.64 | 0.63  (0.55-0.67) | 64 | 68.4 | 64 | 0.31 | 0.65 | 0.66  (0.54-0.78) |
| MCC: Matthews Correlation Coefficient; AUROC: Area under Receiver operating Characterstic curve | | | | | | | | | | | | |
