# Supplementary material for: Expression based biomarkers and models to classify early and late-stage samples of Papillary Thyroid Carcinoma
Source: PLoS One. 2020 Apr 23;15(4):e0231629. doi: 10.1371/journal.pone.0231629 (PMC7179925; doi:10.1371/journal.pone.0231629)
Supplement: S15 Table — (DOCX) [file pone.0231629.s015.docx]

Table S15: 36 features selected using SVC-L1 feature selection

| **Transcript ID** | **Transcript type** | **Gene Symbol** |
| --- | --- | --- |
| ENSG00000237424.1 | antisense | *FOXD2-AS1* |
| ENSG00000249307.4 | antisense | *LINC01088* |
| ENSG00000233968.5 | antisense | *RP11-354E11.2* |
| ENSG00000257732.1 | antisense | *RP11-818F20.5* |
| ENSG00000266955.1 | antisense | *RP11-820I16.3* |
| ENSG00000249602.1 | antisense | *RP11-98D18.3* |
| ENSG00000273203.1 | lincRNA | *AC006946.16* |
| ENSG00000225166.1 | lincRNA | *AC012462.2* |
| ENSG00000236385.1 | lincRNA | *RP11-114M1.2* |
| ENSG00000272970.1 | lincRNA | *RP11-329B9.4* |
| ENSG00000274698.1 | lincRNA | *RP11-71L14.4* |
| ENSG00000274115.1 | miRNA | *MIR6081* |
| ENSG00000278109.1 | misc_RNA | *Metazoa_SRP* |
| ENSG00000221957.7 | polymorphic_pseudogene | *KIR2DS4* |
| ENSG00000258988.1 | processed_pseudogene | *RP11-125H8.1* |
| ENSG00000229979.1 | processed_pseudogene | *U82670.9* |
| ENSG00000162746.13 | protein_coding | *FCRLB* |
| ENSG00000162639.14 | protein_coding | *HENMT1* |
| ENSG00000196787.3 | protein_coding | *HIST1H2AG* |
| ENSG00000105610.4 | protein_coding | *KLF1* |
| ENSG00000128594.6 | protein_coding | *LRRC4* |
| ENSG00000164520.10 | protein_coding | *RAET1E* |
| ENSG00000204764.11 | protein_coding | *RANBP17* |
| ENSG00000170615.13 | protein_coding | *SLC26A5* |
| ENSG00000143570.16 | protein_coding | *SLC39A1* |
| ENSG00000077327.14 | protein_coding | *SPAG6* |
| ENSG00000163071.9 | protein_coding | *SPATA18* |
| ENSG00000277893.1 | protein_coding | *SRD5A2* |
| ENSG00000112837.15 | protein_coding | *TBX18* |
| ENSG00000164362.17 | protein_coding | *TERT* |
| ENSG00000124191.16 | protein_coding | *TOX2* |
| ENSG00000064205.9 | protein_coding | *WISP2* |
| ENSG00000178163.6 | protein_coding | *ZNF518B* |
| ENSG00000235389.2 | sense_intronic | *RP11-134K1.3* |
| ENSG00000243742.4 | transcribed_processed_pseudogene | *RPLP0P2* |
| ENSG00000224295.2 | unprocessed_pseudogene | *AC087380.14* |
