# Supplementary material for: Expression based biomarkers and models to classify early and late-stage samples of Papillary Thyroid Carcinoma
Source: PLoS One. 2020 Apr 23;15(4):e0231629. doi: 10.1371/journal.pone.0231629 (PMC7179925; doi:10.1371/journal.pone.0231629)
Supplement: S16 Table — (DOCX) [file pone.0231629.s016.docx]

Table S16: 107 RNA transcripts selected using SVC L1 for multiclass classification (THCA-NEL-M)

| Transcript | Transcript type | Mapped Name |
| --- | --- | --- |
| ENSG00000269210.2 | antisense | RP11-173C1.1 |
| ENSG00000267746.1 | antisense | RP11-379L18.1 |
| ENSG00000266955.1 | antisense | RP11-820I16.3 |
| ENSG00000255480.1 | antisense | RP4-710M3.2 |
| ENSG00000254873.1 | antisense | RP11-770J1.5 |
| ENSG00000254545.1 | antisense | RP11-84A19.3 |
| ENSG00000254499.1 | antisense | AC002056.5 |
| ENSG00000254163.1 | antisense | CTC-340I23.2 |
| ENSG00000249307.4 | antisense | LINC01088 |
| ENSG00000237424.1 | antisense | FOXD2-AS1 |
| ENSG00000235899.1 | antisense | LINC01564 |
| ENSG00000233191.1 | antisense | AC006372.6 |
| ENSG00000232725.1 | antisense | U52111.14 |
| ENSG00000232194.1 | antisense | RP1-313L4.3 |
| ENSG00000229498.1 | antisense | AC105053.3 |
| ENSG00000226409.1 | antisense | RP11-735G4.1 |
| ENSG00000253338.1 | IG_V_pseudogene | IGLV3-29 |
| ENSG00000274698.1 | lincRNA | RP11-71L14.4 |
| ENSG00000273203.1 | lincRNA | AC006946.16 |
| ENSG00000272970.1 | lincRNA | RP11-329B9.4 |
| ENSG00000271367.1 | lincRNA | RP3-483K16.4 |
| ENSG00000270022.3 | lincRNA | RNU12 |
| ENSG00000267651.1 | lincRNA | RP11-95O2.1 |
| ENSG00000259907.1 | lincRNA | RP6-91H8.2 |
| ENSG00000254142.2 | lincRNA | RP11-53M11.3 |
| ENSG00000251388.1 | lincRNA | RP11-427M20.1 |
| ENSG00000251381.5 | lincRNA | LINC00958 |
| ENSG00000239268.2 | lincRNA | RP11-384F7.2 |
| ENSG00000236385.1 | lincRNA | RP11-114M1.2 |
| ENSG00000233730.1 | lincRNA | RP4-666F24.3 |
| ENSG00000233420.1 | lincRNA | AC002127.4 |
| ENSG00000230021.6 | lincRNA | RP5-857K21.4 |
| ENSG00000228459.3 | lincRNA | LINC01546 |
| ENSG00000266589.1 | miRNA | MIR4512 |
| ENSG00000264539.1 | miRNA | MIR548AR |
| ENSG00000207574.1 | miRNA | MIR661 |
| ENSG00000278109.1 | misc_RNA | Metazoa_SRP |
| ENSG00000276309.1 | misc_RNA | 7SK |
| ENSG00000239279.3 | misc_RNA | RN7SL184P |
| ENSG00000221957.7 | polymorphic_pseudogene | KIR2DS4 |
| ENSG00000262636.1 | processed_pseudogene | CTD-3088G3.4 |
| ENSG00000258988.1 | processed_pseudogene | RP11-125H8.1 |
| ENSG00000258153.1 | processed_pseudogene | HSPE1P4 |
| ENSG00000254463.1 | processed_pseudogene | RP11-484D2.3 |
| ENSG00000234566.1 | processed_pseudogene | RPL7AP71 |
| ENSG00000229890.1 | processed_pseudogene | AC023672.1 |
| ENSG00000218261.1 | processed_pseudogene | RP11-96J19.1 |
| ENSG00000213731.2 | processed_pseudogene | RAB5CP1 |
| ENSG00000266964.4 | protein_coding | FXYD1 |
| ENSG00000215186.6 | protein_coding | GOLGA6B |
| ENSG00000213759.7 | protein_coding | UGT2B11 |
| ENSG00000205221.11 | protein_coding | VIT |
| ENSG00000204764.11 | protein_coding | RANBP17 |
| ENSG00000204065.2 | protein_coding | TCEAL5 |
| ENSG00000203722.6 | protein_coding | RAET1G |
| ENSG00000198890.7 | protein_coding | PRMT6 |
| ENSG00000196787.3 | protein_coding | HIST1H2AG |
| ENSG00000189058.7 | protein_coding | APOD |
| ENSG00000189056.12 | protein_coding | RELN |
| ENSG00000187730.7 | protein_coding | GABRD |
| ENSG00000183615.5 | protein_coding | FAM167B |
| ENSG00000179915.19 | protein_coding | NRXN1 |
| ENSG00000179403.11 | protein_coding | VWA1 |
| ENSG00000178163.6 | protein_coding | ZNF518B |
| ENSG00000177182.9 | protein_coding | CLVS1 |
| ENSG00000170615.13 | protein_coding | SLC26A5 |
| ENSG00000170485.15 | protein_coding | NPAS2 |
| ENSG00000168754.12 | protein_coding | FAM178B |
| ENSG00000167123.17 | protein_coding | CERCAM |
| ENSG00000163071.9 | protein_coding | SPATA18 |
| ENSG00000162746.13 | protein_coding | FCRLB |
| ENSG00000162639.14 | protein_coding | HENMT1 |
| ENSG00000155511.16 | protein_coding | GRIA1 |
| ENSG00000149090.10 | protein_coding | PAMR1 |
| ENSG00000147588.6 | protein_coding | PMP2 |
| ENSG00000143570.16 | protein_coding | SLC39A1 |
| ENSG00000136542.7 | protein_coding | GALNT5 |
| ENSG00000129455.14 | protein_coding | KLK8 |
| ENSG00000128594.6 | protein_coding | LRRC4 |
| ENSG00000124343.11 | protein_coding | XG |
| ENSG00000118946.10 | protein_coding | PCDH17 |
| ENSG00000114646.8 | protein_coding | CSPG5 |
| ENSG00000113555.5 | protein_coding | PCDH12 |
| ENSG00000113552.14 | protein_coding | GNPDA1 |
| ENSG00000111087.8 | protein_coding | GLI1 |
| ENSG00000109832.11 | protein_coding | DDX25 |
| ENSG00000104918.6 | protein_coding | RETN |
| ENSG00000104313.16 | protein_coding | EYA1 |
| ENSG00000100784.8 | protein_coding | RPS6KA5 |
| ENSG00000072657.7 | protein_coding | TRHDE |
| ENSG00000056291.16 | protein_coding | NPFFR2 |
| ENSG00000016402.11 | protein_coding | IL20RA |
| ENSG00000011465.15 | protein_coding | DCN |
| ENSG00000004399.11 | protein_coding | PLXND1 |
| ENSG00000279336.1 | pseudogene | AL353662.2 |
| ENSG00000232031.1 | sense_intronic | RP5-991C6.4 |
| ENSG00000228939.1 | sense_intronic | AKT3-IT1 |
| ENSG00000251405.2 | sense_overlapping | CTB-109A12.1 |
| ENSG00000265185.4 | snoRNA | SNORD3B-1 |
| ENSG00000273768.1 | snRNA | RNU1-1 |
| ENSG00000270722.1 | snRNA | U1 |
| ENSG00000279187.1 | TEC | RP11-455O6.5 |
| ENSG00000211695.2 | TR_V_gene | TRGV9 |
| ENSG00000224287.2 | transcribed_processed_pseudogene | MSL3P1 |
| ENSG00000269552.1 | unprocessed_pseudogene | AC005255.5 |
| ENSG00000254608.1 | unprocessed_pseudogene | RP11-56A10.1 |
| ENSG00000232894.1 | unprocessed_pseudogene | MRPS31P2 |
