# Supplementary material for: Expression based biomarkers and models to classify early and late-stage samples of Papillary Thyroid Carcinoma
Source: PLoS One. 2020 Apr 23;15(4):e0231629. doi: 10.1371/journal.pone.0231629 (PMC7179925; doi:10.1371/journal.pone.0231629)
Supplement: S17 Table — (DOCX) [file pone.0231629.s017.docx]

Table S17: Top genes showing AUROC of >= 0.85 for differentiation between cancer and normal samples

| **Transcript** | **Transcript type** | **Mapped HGNC Gene symbol** | **Mean expression in cancer** | **Mean expression in late** | **AUROC** | **Accuracy** | **Threshold** |
| --- | --- | --- | --- | --- | --- | --- | --- |
| ENSG00000260912.1 | sense_overlapping | *RP11-363E7.4* | 2.59 | 1.41 | 0.96 | 94.84 | more than 1.74 |
| ENSG00000162981.13 | protein_coding | *FAM84A* | 2.10 | 0.64 | 0.95 | 92.83 | more than 1.12 |
| ENSG00000170439.6 | protein_coding | *METTL7B* | 5.03 | 1.62 | 0.95 | 91.03 | more than 3.22 |
| ENSG00000160180.15 | protein_coding | *TFF3* | 3.80 | 9.36 | 0.95 | 92.60 | less than 8.07 |
| ENSG00000183248.10 | protein_coding | *PRR36* | 2.38 | 0.65 | 0.95 | 92.15 | more than 1.14 |
| ENSG00000168528.10 | protein_coding | *SERINC2* | 5.46 | 3.73 | 0.95 | 90.36 | more than 4.69 |
| ENSG00000185090.13 | protein_coding | *MANEAL* | 2.53 | 1.29 | 0.94 | 89.24 | more than 1.76 |
| ENSG00000175832.11 | protein_coding | *ETV4* | 3.62 | 0.98 | 0.94 | 92.38 | more than 2.11 |
| ENSG00000174307.6 | protein_coding | *PHLDA3* | 4.59 | 2.89 | 0.94 | 92.15 | more than 3.62 |
| ENSG00000026652.12 | protein_coding | *AGPAT4* | 0.58 | 1.48 | 0.94 | 92.15 | less than 1.08 |
| ENSG00000147257.12 | protein_coding | *GPC3* | 0.65 | 2.96 | 0.94 | 93.72 | less than 2.00 |
| ENSG00000198626.14 | protein_coding | *RYR2* | 0.36 | 1.94 | 0.94 | 91.93 | less than 1.01 |
| ENSG00000254489.1 | antisense | *RP5-1024C24.1* | 0.30 | 1.69 | 0.94 | 91.93 | less than 1.00 |
| ENSG00000153246.10 | protein_coding | *PLA2R1* | 1.60 | 4.42 | 0.93 | 91.48 | less than 3.16 |
| ENSG00000174567.7 | protein_coding | *GOLT1A* | 3.20 | 0.69 | 0.93 | 87.89 | more than 2.04 |
| ENSG00000162873.13 | protein_coding | *KLHDC8A* | 3.46 | 0.38 | 0.93 | 93.05 | more than 1.05 |
| ENSG00000134569.8 | protein_coding | *LRP4* | 4.27 | 1.14 | 0.93 | 89.46 | more than 2.29 |
| ENSG00000169302.13 | protein_coding | *STK32A* | 3.08 | 1.10 | 0.93 | 87.44 | more than 2.13 |
| ENSG00000138356.12 | protein_coding | *AOX1* | 0.65 | 2.73 | 0.93 | 94.17 | less than 2.00 |
| ENSG00000163898.8 | protein_coding | *LIPH* | 3.68 | 0.31 | 0.93 | 87.22 | more than 2.02 |
| ENSG00000009694.12 | protein_coding | *TENM1* | 2.52 | 0.35 | 0.93 | 87.22 | more than 1.01 |
| ENSG00000074706.12 | protein_coding | *IPCEF1* | 1.18 | 4.14 | 0.93 | 90.58 | less than 3.08 |
| ENSG00000204381.10 | protein_coding | *LAYN* | 1.78 | 3.52 | 0.93 | 88.79 | less than 2.49 |
| ENSG00000128573.21 | protein_coding | *FOXP2* | 0.43 | 1.34 | 0.93 | 92.15 | less than 1.00 |
| ENSG00000198774.4 | protein_coding | *RASSF9* | 0.49 | 1.78 | 0.93 | 90.36 | less than 1.00 |
| ENSG00000143196.4 | protein_coding | *DPT* | 1.70 | 5.66 | 0.93 | 90.36 | less than 4.00 |
| ENSG00000117308.13 | protein_coding | *GALE* | 4.00 | 1.63 | 0.93 | 90.13 | more than 2.08 |
| ENSG00000050767.14 | protein_coding | *COL23A1* | 3.26 | 5.90 | 0.93 | 90.13 | less than 5.17 |
| ENSG00000196616.11 | protein_coding | *ADH1B* | 0.40 | 2.43 | 0.93 | 88.34 | less than 1.00 |
| ENSG00000163235.14 | protein_coding | *TGFA* | 4.05 | 2.11 | 0.92 | 89.91 | more than 2.64 |
| ENSG00000153823.17 | protein_coding | *PID1* | 0.54 | 2.02 | 0.92 | 88.12 | less than 1.03 |
| ENSG00000143171.11 | protein_coding | *RXRG* | 4.49 | 0.58 | 0.92 | 88.12 | more than 2.04 |
| ENSG00000119655.7 | protein_coding | *NPC2* | 9.34 | 7.00 | 0.92 | 93.27 | more than 7.47 |
| ENSG00000185774.13 | protein_coding | *KCNIP4* | 0.64 | 1.86 | 0.92 | 89.69 | less than 1.07 |
| ENSG00000244405.6 | protein_coding | *ETV5* | 3.94 | 2.48 | 0.92 | 91.26 | more than 3.08 |
| ENSG00000171368.11 | protein_coding | *TPPP* | 1.38 | 3.29 | 0.92 | 89.46 | less than 2.23 |
| ENSG00000062038.12 | protein_coding | *CDH3* | 3.90 | 1.04 | 0.92 | 89.46 | more than 2.06 |
| ENSG00000109586.10 | protein_coding | *GALNT7* | 3.79 | 2.11 | 0.92 | 85.87 | more than 2.96 |
| ENSG00000184489.10 | protein_coding | *PTP4A3* | 4.13 | 2.40 | 0.92 | 89.01 | more than 3.37 |
| ENSG00000150672.15 | protein_coding | *DLG2* | 0.55 | 2.10 | 0.92 | 87.22 | less than 1.04 |
| ENSG00000196666.4 | protein_coding | *FAM180B* | 0.33 | 1.98 | 0.92 | 90.58 | less than 1.00 |
| ENSG00000168702.15 | protein_coding | *LRP1B* | 0.31 | 1.83 | 0.92 | 90.58 | less than 1.00 |
| ENSG00000187730.7 | protein_coding | *GABRD* | 2.04 | 0.42 | 0.92 | 87.00 | more than 1.07 |
| ENSG00000271367.1 | lincRNA | *RP3-483K16.4* | 0.56 | 2.33 | 0.92 | 87.00 | less than 1.00 |
| ENSG00000157111.11 | protein_coding | *TMEM171* | 2.23 | 4.54 | 0.92 | 86.77 | less than 3.19 |
| ENSG00000196781.12 | protein_coding | *TLE1* | 2.17 | 3.35 | 0.92 | 88.34 | less than 2.81 |
| ENSG00000105717.12 | protein_coding | *PBX4* | 1.41 | 3.29 | 0.92 | 88.34 | less than 2.26 |
| ENSG00000147408.13 | protein_coding | *CSGALNACT1* | 3.05 | 5.08 | 0.92 | 86.55 | less than 4.04 |
| ENSG00000109205.15 | protein_coding | *ODAM* | 0.34 | 1.75 | 0.91 | 89.91 | less than 1.00 |
| ENSG00000072682.17 | protein_coding | *P4HA2* | 3.42 | 1.90 | 0.91 | 88.12 | more than 2.77 |
| ENSG00000176532.3 | protein_coding | *PRR15* | 4.90 | 0.72 | 0.91 | 86.32 | more than 2.19 |
| ENSG00000138722.8 | protein_coding | *MMRN1* | 0.82 | 3.22 | 0.91 | 91.48 | less than 2.03 |
| ENSG00000135404.10 | protein_coding | *CD63* | 8.70 | 7.82 | 0.91 | 91.48 | more than 8.12 |
| ENSG00000151491.11 | protein_coding | *EPS8* | 4.31 | 2.76 | 0.91 | 84.53 | more than 3.59 |
| ENSG00000138185.15 | protein_coding | *ENTPD1* | 3.97 | 2.07 | 0.91 | 84.53 | more than 3.14 |
| ENSG00000250343.1 | antisense | *CTC-255N20.1* | 1.70 | 0.40 | 0.91 | 84.53 | more than 1.00 |
| ENSG00000126218.10 | protein_coding | *F10* | 0.55 | 1.80 | 0.91 | 89.69 | less than 1.01 |
| ENSG00000114698.13 | protein_coding | *PLSCR4* | 2.51 | 3.96 | 0.91 | 87.89 | less than 3.17 |
| ENSG00000105711.9 | protein_coding | *SCN1B* | 2.44 | 0.96 | 0.91 | 87.89 | more than 1.44 |
| ENSG00000073792.14 | protein_coding | *IGF2BP2* | 3.49 | 2.13 | 0.91 | 86.10 | more than 3.02 |
| ENSG00000166589.11 | protein_coding | *CDH16* | 1.41 | 5.01 | 0.91 | 91.26 | less than 4.01 |
| ENSG00000159166.12 | protein_coding | *LAD1* | 3.71 | 1.84 | 0.91 | 91.26 | more than 2.25 |
| ENSG00000107281.8 | protein_coding | *NPDC1* | 4.94 | 3.37 | 0.91 | 89.46 | more than 4.08 |
| ENSG00000136111.11 | protein_coding | *TBC1D4* | 2.47 | 4.31 | 0.91 | 94.62 | less than 3.87 |
| ENSG00000132561.12 | protein_coding | *MATN2* | 3.93 | 6.67 | 0.91 | 92.83 | less than 5.93 |
| ENSG00000184574.8 | protein_coding | *LPAR5* | 3.87 | 1.42 | 0.91 | 84.08 | more than 3.07 |
| ENSG00000161714.10 | protein_coding | *PLCD3* | 3.96 | 1.59 | 0.91 | 84.08 | more than 2.65 |
| ENSG00000197635.8 | protein_coding | *DPP4* | 4.36 | 1.09 | 0.91 | 87.44 | more than 2.12 |
| ENSG00000259803.5 | protein_coding | *SLC22A31* | 3.64 | 0.19 | 0.91 | 85.65 | more than 1.00 |
| ENSG00000177191.2 | protein_coding | *B3GNT8* | 2.03 | 0.85 | 0.91 | 89.01 | more than 1.16 |
| ENSG00000198682.11 | protein_coding | *PAPSS2* | 1.67 | 3.99 | 0.91 | 94.17 | less than 3.35 |
| ENSG00000141639.10 | protein_coding | *MAPK4* | 0.49 | 2.32 | 0.91 | 85.43 | less than 1.01 |
| ENSG00000166145.13 | protein_coding | *SPINT1* | 6.10 | 4.66 | 0.91 | 83.63 | more than 5.46 |
| ENSG00000204065.2 | protein_coding | *TCEAL5* | 0.41 | 2.14 | 0.91 | 88.79 | less than 1.00 |
| ENSG00000044524.9 | protein_coding | *EPHA3* | 1.20 | 2.84 | 0.91 | 88.79 | less than 2.03 |
| ENSG00000164764.10 | protein_coding | *SBSPON* | 0.48 | 1.43 | 0.91 | 93.95 | less than 1.05 |
| ENSG00000135205.13 | protein_coding | *CCDC146* | 1.61 | 3.10 | 0.91 | 87.00 | less than 2.33 |
| ENSG00000172667.9 | protein_coding | *ZMAT3* | 3.36 | 2.00 | 0.91 | 92.15 | more than 2.37 |
| ENSG00000147231.12 | protein_coding | *CXorf57* | 0.73 | 1.45 | 0.91 | 92.15 | less than 1.12 |
| ENSG00000130226.15 | protein_coding | *DPP6* | 0.63 | 2.93 | 0.91 | 90.36 | less than 2.00 |
| ENSG00000115380.17 | protein_coding | *EFEMP1* | 3.81 | 5.95 | 0.91 | 88.57 | less than 5.26 |
| ENSG00000104723.19 | protein_coding | *TUSC3* | 3.58 | 1.35 | 0.91 | 88.57 | more than 2.11 |
| ENSG00000091129.18 | protein_coding | *NRCAM* | 2.99 | 1.41 | 0.91 | 88.57 | more than 2.07 |
| ENSG00000151365.2 | protein_coding | *THRSP* | 3.59 | 1.16 | 0.91 | 86.77 | more than 2.24 |
| ENSG00000115828.14 | protein_coding | *QPCT* | 4.51 | 1.86 | 0.91 | 84.98 | more than 3.17 |
| ENSG00000145362.15 | protein_coding | *ANK2* | 0.64 | 2.11 | 0.91 | 83.18 | less than 1.01 |
| ENSG00000135723.12 | protein_coding | *FHOD1* | 3.61 | 2.47 | 0.91 | 88.34 | more than 2.92 |
| ENSG00000258117.2 | lincRNA | *RP11-1022B3.1* | 0.39 | 2.00 | 0.91 | 86.55 | less than 1.00 |
| ENSG00000198919.11 | protein_coding | *DZIP3* | 2.27 | 2.99 | 0.91 | 91.70 | less than 2.78 |
| ENSG00000223914.1 | lincRNA | *AC079630.2* | 3.64 | 0.35 | 0.91 | 89.91 | more than 1.00 |
| ENSG00000110092.3 | protein_coding | *CCND1* | 6.60 | 4.96 | 0.91 | 82.96 | more than 6.10 |
| ENSG00000109182.10 | protein_coding | *CWH43* | 0.99 | 3.24 | 0.91 | 82.96 | less than 2.00 |
| ENSG00000174460.3 | protein_coding | *ZCCHC12* | 6.42 | 1.24 | 0.90 | 88.12 | more than 3.06 |
| ENSG00000166148.3 | protein_coding | *AVPR1A* | 0.95 | 2.52 | 0.90 | 89.69 | less than 2.01 |
| ENSG00000231445.1 | processed_pseudogene | *TIMM8AP1* | 2.17 | 0.24 | 0.90 | 82.74 | more than 1.00 |
| ENSG00000137077.6 | protein_coding | *CCL21* | 2.58 | 7.70 | 0.90 | 87.89 | less than 6.00 |
| ENSG00000075035.8 | protein_coding | *WSCD2* | 1.14 | 3.97 | 0.90 | 87.89 | less than 3.00 |
| ENSG00000070371.14 | protein_coding | *CLTCL1* | 0.90 | 1.53 | 0.90 | 87.89 | less than 1.19 |
| ENSG00000120820.11 | protein_coding | *GLT8D2* | 1.88 | 3.95 | 0.90 | 89.46 | less than 3.23 |
| ENSG00000163485.14 | protein_coding | *ADORA1* | 3.21 | 1.35 | 0.90 | 87.67 | more than 2.11 |
| ENSG00000166780.9 | protein_coding | *C16orf45* | 3.45 | 2.13 | 0.90 | 85.87 | more than 2.77 |
| ENSG00000154175.15 | protein_coding | *ABI3BP* | 1.63 | 3.59 | 0.90 | 91.03 | less than 3.04 |
| ENSG00000277363.3 | protein_coding | *SRCIN1* | 1.48 | 0.26 | 0.90 | 82.29 | more than 1.01 |
| ENSG00000198121.12 | protein_coding | *LPAR1* | 1.76 | 3.04 | 0.90 | 82.29 | less than 2.22 |
| ENSG00000111907.19 | protein_coding | *TPD52L1* | 4.13 | 2.70 | 0.90 | 82.29 | more than 3.47 |
| ENSG00000197467.12 | protein_coding | *COL13A1* | 1.80 | 0.34 | 0.90 | 82.06 | more than 1.08 |
| ENSG00000149573.7 | protein_coding | *MPZL2* | 5.63 | 3.57 | 0.90 | 82.06 | more than 4.87 |
| ENSG00000112232.8 | protein_coding | *KHDRBS2* | 1.07 | 2.92 | 0.90 | 85.43 | less than 2.00 |
| ENSG00000169435.12 | protein_coding | *RASSF6* | 0.45 | 1.73 | 0.90 | 90.58 | less than 1.01 |
| ENSG00000129925.9 | protein_coding | *TMEM8A* | 4.54 | 3.74 | 0.90 | 90.58 | more than 4.07 |
| ENSG00000237396.1 | lincRNA | *LINC01384* | 0.28 | 1.76 | 0.90 | 90.58 | less than 1.00 |
| ENSG00000131015.4 | protein_coding | *ULBP2* | 1.85 | 0.55 | 0.90 | 83.63 | more than 1.06 |
| ENSG00000087088.18 | protein_coding | *BAX* | 4.60 | 3.61 | 0.90 | 88.79 | more than 4.01 |
| ENSG00000147255.16 | protein_coding | *IGSF1* | 4.91 | 1.73 | 0.90 | 81.84 | more than 3.01 |
| ENSG00000165023.5 | protein_coding | *DIRAS2* | 0.45 | 1.52 | 0.90 | 87.00 | less than 1.00 |
| ENSG00000160181.7 | protein_coding | *TFF2* | 0.23 | 1.72 | 0.90 | 92.15 | less than 1.00 |
| ENSG00000014914.18 | protein_coding | *MTMR11* | 1.82 | 0.73 | 0.90 | 83.41 | more than 1.18 |
| ENSG00000156966.6 | protein_coding | *B3GNT7* | 2.55 | 1.02 | 0.90 | 81.61 | more than 2.10 |
| ENSG00000132535.17 | protein_coding | *DLG4* | 2.83 | 1.72 | 0.90 | 81.61 | more than 2.44 |
| ENSG00000256870.2 | protein_coding | *SLC5A8* | 1.65 | 4.52 | 0.90 | 86.77 | less than 4.00 |
| ENSG00000106541.10 | protein_coding | *AGR2* | 3.82 | 1.15 | 0.90 | 86.77 | more than 2.00 |
| ENSG00000125931.9 | protein_coding | *CITED1* | 5.99 | 1.96 | 0.90 | 84.98 | more than 4.05 |
| ENSG00000101938.13 | protein_coding | *CHRDL1* | 1.03 | 3.99 | 0.90 | 83.18 | less than 2.00 |
| ENSG00000233251.6 | antisense | *AC007743.1* | 0.64 | 1.54 | 0.90 | 86.55 | less than 1.03 |
| ENSG00000165886.4 | protein_coding | *UBTD1* | 4.24 | 3.26 | 0.90 | 91.70 | more than 3.63 |
| ENSG00000185238.11 | protein_coding | *PRMT3* | 1.93 | 2.61 | 0.90 | 84.75 | less than 2.24 |
| ENSG00000165061.13 | protein_coding | *ZMAT4* | 0.90 | 2.98 | 0.90 | 84.75 | less than 2.00 |
| ENSG00000137142.4 | protein_coding | *IGFBPL1* | 1.04 | 3.43 | 0.90 | 84.75 | less than 2.00 |
| ENSG00000249249.1 | antisense | *AC010226.4* | 0.64 | 1.30 | 0.90 | 89.91 | less than 1.04 |
| ENSG00000114805.15 | protein_coding | *PLCH1* | 0.74 | 2.29 | 0.90 | 95.07 | less than 2.03 |
| ENSG00000279444.1 | TEC | *RP11-715H19.2* | 0.55 | 2.63 | 0.90 | 82.96 | less than 1.00 |
| ENSG00000146070.15 | protein_coding | *PLA2G7* | 2.63 | 4.12 | 0.90 | 81.17 | less than 3.32 |
| ENSG00000153993.12 | protein_coding | *SEMA3D* | 1.06 | 3.78 | 0.89 | 89.69 | less than 3.00 |
| ENSG00000188906.12 | protein_coding | *LRRK2* | 3.92 | 1.22 | 0.89 | 86.10 | more than 2.12 |
| ENSG00000141985.8 | protein_coding | *SH3GL1* | 4.71 | 4.01 | 0.89 | 91.26 | more than 4.18 |
| ENSG00000006625.16 | protein_coding | *GGCT* | 5.35 | 3.54 | 0.89 | 84.30 | more than 4.27 |
| ENSG00000266524.2 | protein_coding | *GDF10* | 0.59 | 2.44 | 0.89 | 82.51 | less than 1.00 |
| ENSG00000011028.12 | protein_coding | *MRC2* | 4.51 | 2.19 | 0.89 | 82.51 | more than 3.53 |
| ENSG00000105427.8 | protein_coding | *CNFN* | 2.19 | 0.81 | 0.89 | 87.67 | more than 1.18 |
| ENSG00000131018.21 | protein_coding | *SYNE1* | 1.42 | 2.94 | 0.89 | 85.87 | less than 2.14 |
| ENSG00000147606.7 | protein_coding | *SLC26A7* | 4.09 | 7.35 | 0.89 | 84.08 | less than 6.01 |
| ENSG00000213977.6 | protein_coding | *TAX1BP3* | 4.44 | 3.48 | 0.89 | 89.24 | more than 3.77 |
| ENSG00000182348.6 | protein_coding | *ZNF804B* | 0.31 | 2.05 | 0.89 | 87.44 | less than 1.00 |
| ENSG00000164169.11 | protein_coding | *PRMT9* | 2.25 | 3.00 | 0.89 | 87.44 | less than 2.67 |
| ENSG00000154553.12 | protein_coding | *PDLIM3* | 1.22 | 2.92 | 0.89 | 87.44 | less than 2.11 |
| ENSG00000187583.9 | protein_coding | *PLEKHN1* | 1.78 | 0.29 | 0.89 | 80.49 | more than 1.06 |
| ENSG00000184916.7 | protein_coding | *JAG2* | 2.52 | 1.49 | 0.89 | 89.01 | more than 1.93 |
| ENSG00000117298.13 | protein_coding | *ECE1* | 6.21 | 4.87 | 0.89 | 82.06 | more than 5.67 |
| ENSG00000103160.10 | protein_coding | *HSDL1* | 3.14 | 4.01 | 0.89 | 87.22 | less than 3.55 |
| ENSG00000133800.7 | protein_coding | *LYVE1* | 0.86 | 3.11 | 0.89 | 92.38 | less than 2.03 |
| ENSG00000126878.11 | protein_coding | *AIF1L* | 4.36 | 6.03 | 0.89 | 92.38 | less than 5.63 |
| ENSG00000149294.15 | protein_coding | *NCAM1* | 1.71 | 3.67 | 0.89 | 85.43 | less than 3.02 |
| ENSG00000112964.12 | protein_coding | *GHR* | 0.55 | 1.50 | 0.89 | 90.58 | less than 1.07 |
| ENSG00000197249.11 | protein_coding | *SERPINA1* | 7.45 | 3.69 | 0.89 | 81.84 | more than 5.42 |
| ENSG00000109466.12 | protein_coding | *KLHL2* | 3.68 | 3.00 | 0.89 | 81.84 | more than 3.34 |
| ENSG00000158352.14 | protein_coding | *SHROOM4* | 2.78 | 1.05 | 0.89 | 92.15 | more than 1.39 |
| ENSG00000131981.14 | protein_coding | *LGALS3* | 6.76 | 4.66 | 0.89 | 80.04 | more than 5.99 |
| ENSG00000168079.15 | protein_coding | *SCARA5* | 0.45 | 2.68 | 0.89 | 85.20 | less than 1.00 |
| ENSG00000165959.10 | protein_coding | *CLMN* | 0.71 | 1.73 | 0.89 | 83.41 | less than 1.05 |
| ENSG00000136155.15 | protein_coding | *SCEL* | 3.59 | 0.93 | 0.89 | 83.41 | more than 2.03 |
| ENSG00000244694.6 | protein_coding | *PTCHD4* | 2.11 | 0.51 | 0.89 | 88.57 | more than 1.06 |
| ENSG00000174839.11 | protein_coding | *DENND6A* | 2.53 | 3.51 | 0.89 | 93.72 | less than 3.28 |
| ENSG00000136205.15 | protein_coding | *TNS3* | 2.97 | 4.39 | 0.89 | 90.13 | less than 3.87 |
| ENSG00000066248.13 | protein_coding | *NGEF* | 2.79 | 0.30 | 0.89 | 83.18 | more than 1.00 |
| ENSG00000144749.12 | protein_coding | *LRIG1* | 2.53 | 3.97 | 0.89 | 93.50 | less than 3.63 |
| ENSG00000066382.15 | protein_coding | *MPPED2* | 1.07 | 3.19 | 0.89 | 81.39 | less than 2.01 |
| ENSG00000115112.7 | protein_coding | *TFCP2L1* | 1.54 | 4.51 | 0.89 | 86.55 | less than 3.10 |
| ENSG00000256268.1 | lincRNA | *RP11-221N13.3* | 1.52 | 0.13 | 0.89 | 79.60 | more than 1.00 |
| ENSG00000143217.8 | protein_coding | *PVRL4* | 2.78 | 0.42 | 0.89 | 84.75 | more than 1.03 |
| ENSG00000197766.6 | protein_coding | *CFD* | 2.93 | 5.26 | 0.89 | 86.32 | less than 3.90 |
| ENSG00000104611.10 | protein_coding | *SH2D4A* | 3.85 | 2.87 | 0.89 | 86.32 | more than 3.37 |
| ENSG00000196218.10 | protein_coding | *RYR1* | 1.57 | 0.36 | 0.89 | 79.37 | more than 1.01 |
| ENSG00000163900.9 | protein_coding | *TMEM41A* | 2.33 | 1.65 | 0.89 | 79.37 | more than 2.16 |
| ENSG00000141391.12 | protein_coding | *SLMO1* | 1.57 | 0.64 | 0.89 | 79.37 | more than 1.16 |
| ENSG00000167749.10 | protein_coding | *KLK4* | 0.60 | 1.80 | 0.88 | 84.53 | less than 1.00 |
| ENSG00000003436.13 | protein_coding | *TFPI* | 0.85 | 1.80 | 0.88 | 82.74 | less than 1.13 |
| ENSG00000113361.11 | protein_coding | *CDH6* | 1.99 | 0.51 | 0.88 | 80.94 | more than 1.09 |
| ENSG00000064270.11 | protein_coding | *ATP2C2* | 0.61 | 2.28 | 0.88 | 80.94 | less than 1.01 |
| ENSG00000114473.12 | protein_coding | *IQCG* | 1.04 | 1.70 | 0.88 | 91.26 | less than 1.48 |
| ENSG00000115525.15 | protein_coding | *ST3GAL5* | 5.25 | 4.09 | 0.88 | 79.15 | more than 4.91 |
| ENSG00000105707.12 | protein_coding | *HPN* | 4.52 | 3.12 | 0.88 | 79.15 | more than 4.02 |
| ENSG00000164761.7 | protein_coding | *TNFRSF11B* | 3.30 | 5.88 | 0.88 | 89.46 | less than 5.31 |
| ENSG00000157680.14 | protein_coding | *DGKI* | 0.98 | 3.05 | 0.88 | 87.67 | less than 2.04 |
| ENSG00000173715.14 | protein_coding | *C11orf80* | 2.37 | 1.35 | 0.88 | 85.87 | more than 1.69 |
| ENSG00000204839.7 | protein_coding | *MROH6* | 1.70 | 0.57 | 0.88 | 78.92 | more than 1.11 |
| ENSG00000261399.1 | antisense | *LA16c-329F2.1* | 1.28 | 3.33 | 0.88 | 78.92 | less than 2.00 |
| ENSG00000183049.11 | protein_coding | *CAMK1D* | 2.17 | 3.45 | 0.88 | 84.08 | less than 2.78 |
| ENSG00000162545.5 | protein_coding | *CAMK2N1* | 5.28 | 2.79 | 0.88 | 84.08 | more than 3.66 |
| ENSG00000142686.7 | protein_coding | *C1orf216* | 2.54 | 1.87 | 0.88 | 84.08 | more than 2.16 |
| ENSG00000151948.10 | protein_coding | *GLT1D1* | 2.19 | 0.61 | 0.88 | 89.24 | more than 1.02 |
| ENSG00000157404.14 | protein_coding | *KIT* | 1.98 | 4.50 | 0.88 | 82.29 | less than 3.00 |
| ENSG00000135824.11 | protein_coding | *RGS8* | 0.45 | 2.30 | 0.88 | 85.65 | less than 1.00 |
| ENSG00000034971.13 | protein_coding | *MYOC* | 0.46 | 2.77 | 0.88 | 85.65 | less than 1.00 |
| ENSG00000150995.16 | protein_coding | *ITPR1* | 2.13 | 4.26 | 0.88 | 83.86 | less than 3.36 |
| ENSG00000130513.6 | protein_coding | *GDF15* | 4.85 | 1.62 | 0.88 | 83.86 | more than 3.17 |
| ENSG00000161013.15 | protein_coding | *MGAT4B* | 4.96 | 4.01 | 0.88 | 82.06 | more than 4.39 |
| ENSG00000011465.15 | protein_coding | *DCN* | 2.47 | 5.04 | 0.88 | 82.06 | less than 4.01 |
| ENSG00000253250.2 | protein_coding | *C8orf88* | 0.87 | 2.48 | 0.88 | 92.38 | less than 2.00 |
| ENSG00000166455.12 | protein_coding | *C16orf46* | 1.47 | 2.37 | 0.88 | 92.38 | less than 2.13 |
| ENSG00000153094.20 | protein_coding | *BCL2L11* | 2.63 | 3.60 | 0.88 | 92.38 | less than 3.31 |
| ENSG00000184500.13 | protein_coding | *PROS1* | 5.43 | 2.77 | 0.88 | 80.27 | more than 4.27 |
| ENSG00000268307.1 | lincRNA | *CTD-2619J13.13* | 2.31 | 0.47 | 0.88 | 85.43 | more than 1.00 |
| ENSG00000120254.14 | protein_coding | *MTHFD1L* | 2.80 | 1.83 | 0.88 | 90.58 | more than 2.17 |
| ENSG00000139211.6 | protein_coding | *AMIGO2* | 3.01 | 1.43 | 0.88 | 78.48 | more than 2.32 |
| ENSG00000110042.6 | protein_coding | *DTX4* | 5.42 | 2.91 | 0.88 | 78.48 | more than 4.30 |
| ENSG00000104044.14 | protein_coding | *OCA2* | 0.69 | 2.57 | 0.88 | 78.48 | less than 1.00 |
| ENSG00000100600.13 | protein_coding | *LGMN* | 4.96 | 5.84 | 0.88 | 78.48 | less than 5.39 |
| ENSG00000155792.8 | protein_coding | *DEPTOR* | 4.04 | 6.06 | 0.88 | 83.63 | less than 5.22 |
| ENSG00000106683.13 | protein_coding | *LIMK1* | 3.57 | 2.68 | 0.88 | 88.79 | more than 3.02 |
| ENSG00000140743.6 | protein_coding | *CDR2* | 3.68 | 4.61 | 0.88 | 93.95 | less than 4.32 |
| ENSG00000171791.11 | protein_coding | *BCL2* | 2.99 | 4.64 | 0.88 | 81.84 | less than 3.81 |
| ENSG00000234546.2 | lincRNA | *RP3-510D11.2* | 1.51 | 0.74 | 0.88 | 87.00 | more than 1.06 |
| ENSG00000196872.9 | protein_coding | *KIAA1211L* | 2.60 | 1.83 | 0.88 | 92.15 | more than 2.04 |
| ENSG00000172159.14 | protein_coding | *FRMD3* | 4.35 | 2.45 | 0.88 | 80.04 | more than 3.58 |
| ENSG00000118257.15 | protein_coding | *NRP2* | 3.54 | 2.01 | 0.88 | 80.04 | more than 2.68 |
| ENSG00000225342.2 | antisense | *AC079630.4* | 3.15 | 0.79 | 0.88 | 80.04 | more than 2.04 |
| ENSG00000148700.12 | protein_coding | *ADD3* | 4.14 | 5.19 | 0.88 | 85.20 | less than 4.71 |
| ENSG00000241155.1 | antisense | *ARHGAP31-AS1* | 0.63 | 1.20 | 0.88 | 90.36 | less than 1.00 |
| ENSG00000145864.11 | protein_coding | *GABRB2* | 2.81 | 0.10 | 0.88 | 78.25 | more than 1.01 |
| ENSG00000188910.7 | protein_coding | *GJB3* | 2.58 | 0.32 | 0.88 | 83.41 | more than 1.00 |
| ENSG00000163431.12 | protein_coding | *LMOD1* | 2.59 | 5.03 | 0.88 | 83.41 | less than 3.64 |
| ENSG00000155366.15 | protein_coding | *RHOC* | 5.62 | 4.94 | 0.88 | 83.41 | more than 5.28 |
| ENSG00000145087.11 | protein_coding | *STXBP5L* | 0.46 | 1.78 | 0.88 | 83.41 | less than 1.00 |
| ENSG00000182580.2 | protein_coding | *EPHB3* | 2.25 | 0.54 | 0.88 | 81.61 | more than 1.09 |
| ENSG00000113946.3 | protein_coding | *CLDN16* | 3.87 | 0.66 | 0.88 | 81.61 | more than 2.02 |
| ENSG00000134548.8 | protein_coding | *SPX* | 1.45 | 3.58 | 0.88 | 86.77 | less than 3.03 |
| ENSG00000260943.1 | lincRNA | *RP11-476D10.1* | 2.42 | 0.12 | 0.88 | 79.82 | more than 1.00 |
| ENSG00000173218.13 | protein_coding | *VANGL1* | 1.67 | 2.51 | 0.88 | 88.34 | less than 2.16 |
| ENSG00000187824.7 | protein_coding | *TMEM220* | 1.77 | 2.92 | 0.88 | 81.39 | less than 2.25 |
| ENSG00000123700.4 | protein_coding | *KCNJ2* | 2.38 | 0.55 | 0.88 | 81.39 | more than 1.17 |
| ENSG00000271614.1 | lincRNA | *LINC00936* | 0.69 | 1.40 | 0.88 | 86.55 | less than 1.00 |
| ENSG00000109113.16 | protein_coding | *RAB34* | 5.48 | 4.53 | 0.88 | 89.91 | more than 4.72 |
| ENSG00000117791.14 | protein_coding | *Mar-02* | 3.24 | 4.46 | 0.88 | 82.96 | less than 4.06 |
| ENSG00000278962.1 | TEC | *RP11-399B17.1* | 0.99 | 2.29 | 0.88 | 93.27 | less than 2.00 |
| ENSG00000120693.12 | protein_coding | *SMAD9* | 2.64 | 4.29 | 0.88 | 81.17 | less than 3.39 |
| ENSG00000178531.5 | protein_coding | *CTXN1* | 3.54 | 1.36 | 0.88 | 79.37 | more than 2.47 |
| ENSG00000196177.11 | protein_coding | *ACADSB* | 2.26 | 2.97 | 0.88 | 84.53 | less than 2.67 |
| ENSG00000166402.7 | protein_coding | *TUB* | 1.80 | 2.95 | 0.88 | 84.53 | less than 2.41 |
| ENSG00000164442.9 | protein_coding | *CITED2* | 5.75 | 7.64 | 0.88 | 89.69 | less than 6.97 |
| ENSG00000273132.1 | antisense | *RP11-350J20.12* | 1.72 | 0.20 | 0.88 | 77.58 | more than 1.00 |
| ENSG00000171714.10 | protein_coding | *ANO5* | 0.75 | 1.64 | 0.87 | 80.94 | less than 1.03 |
| ENSG00000169071.13 | protein_coding | *ROR2* | 1.26 | 2.69 | 0.87 | 80.94 | less than 2.01 |
| ENSG00000254343.2 | lincRNA | *RP11-760H22.2* | 3.10 | 4.72 | 0.87 | 80.94 | less than 3.87 |
| ENSG00000187513.8 | protein_coding | *GJA4* | 4.85 | 3.36 | 0.87 | 86.10 | more than 3.92 |
| ENSG00000068024.15 | protein_coding | *HDAC4* | 1.04 | 1.84 | 0.87 | 91.26 | less than 1.46 |
| ENSG00000088992.16 | protein_coding | *TESC* | 4.93 | 2.52 | 0.87 | 79.15 | more than 3.83 |
| ENSG00000127616.16 | protein_coding | *SMARCA4* | 4.06 | 3.52 | 0.87 | 84.30 | more than 3.79 |
| ENSG00000172201.9 | protein_coding | *ID4* | 6.52 | 8.06 | 0.87 | 89.46 | less than 7.67 |
| ENSG00000013619.12 | protein_coding | *MAMLD1* | 1.86 | 0.59 | 0.87 | 77.35 | more than 1.20 |
| ENSG00000168447.9 | protein_coding | *SCNN1B* | 1.42 | 2.54 | 0.87 | 82.51 | less than 2.05 |
| ENSG00000165092.11 | protein_coding | *ALDH1A1* | 5.44 | 7.14 | 0.87 | 87.67 | less than 6.75 |
| ENSG00000160183.12 | protein_coding | *TMPRSS3* | 0.66 | 1.66 | 0.87 | 80.72 | less than 1.00 |
| ENSG00000150625.15 | protein_coding | *GPM6A* | 0.61 | 2.37 | 0.87 | 80.72 | less than 1.00 |
| ENSG00000187122.15 | protein_coding | *SLIT1* | 2.42 | 0.14 | 0.87 | 77.13 | more than 1.03 |
| ENSG00000162576.15 | protein_coding | *MXRA8* | 5.78 | 4.05 | 0.87 | 77.13 | more than 5.20 |
| ENSG00000105376.4 | protein_coding | *ICAM5* | 2.18 | 0.40 | 0.87 | 82.29 | more than 1.01 |
| ENSG00000079101.15 | protein_coding | *CLUL1* | 0.71 | 1.87 | 0.87 | 82.29 | less than 1.03 |
| ENSG00000231856.2 | antisense | *RP11-327P2.5* | 1.09 | 1.91 | 0.87 | 82.29 | less than 1.41 |
| ENSG00000197905.7 | protein_coding | *TEAD4* | 2.43 | 3.34 | 0.87 | 87.44 | less than 3.05 |
| ENSG00000008256.14 | protein_coding | *CYTH3* | 3.08 | 4.21 | 0.87 | 92.60 | less than 3.96 |
| ENSG00000184908.16 | protein_coding | *CLCNKB* | 1.42 | 3.34 | 0.87 | 80.49 | less than 2.07 |
| ENSG00000174899.9 | protein_coding | *PQLC2L* | 0.74 | 1.71 | 0.87 | 80.49 | less than 1.00 |
| ENSG00000133121.19 | protein_coding | *STARD13* | 2.15 | 3.46 | 0.87 | 83.86 | less than 2.72 |
| ENSG00000198205.6 | protein_coding | *ZXDA* | 1.02 | 1.45 | 0.87 | 89.01 | less than 1.31 |
| ENSG00000184905.7 | protein_coding | *TCEAL2* | 2.35 | 5.06 | 0.87 | 82.06 | less than 4.01 |
| ENSG00000137460.7 | protein_coding | *FHDC1* | 2.53 | 3.99 | 0.87 | 92.38 | less than 3.64 |
| ENSG00000115705.19 | protein_coding | *TPO* | 5.10 | 9.21 | 0.87 | 80.27 | less than 8.03 |
| ENSG00000149948.12 | protein_coding | *HMGA2* | 1.79 | 0.09 | 0.87 | 78.48 | more than 1.00 |
| ENSG00000131016.15 | protein_coding | *AKAP12* | 2.47 | 3.91 | 0.87 | 83.63 | less than 3.31 |
| ENSG00000008277.13 | protein_coding | *ADAM22* | 0.53 | 1.34 | 0.87 | 88.79 | less than 1.05 |
| ENSG00000238271.2 | unprocessed_pseudogene | *IFNWP19* | 1.77 | 0.55 | 0.87 | 76.68 | more than 1.00 |
| ENSG00000198113.2 | protein_coding | *TOR4A* | 2.97 | 2.11 | 0.87 | 76.68 | more than 2.78 |
| ENSG00000168490.12 | protein_coding | *PHYHIP* | 2.61 | 1.23 | 0.87 | 76.68 | more than 2.05 |
| ENSG00000006534.14 | protein_coding | *ALDH3B1* | 2.64 | 1.06 | 0.87 | 76.68 | more than 2.29 |
| ENSG00000004399.11 | protein_coding | *PLXND1* | 4.24 | 2.90 | 0.87 | 76.68 | more than 3.94 |
| ENSG00000080493.12 | protein_coding | *SLC4A4* | 1.56 | 3.98 | 0.87 | 81.84 | less than 3.01 |
| ENSG00000174482.9 | protein_coding | *LINGO2* | 0.37 | 1.53 | 0.87 | 87.00 | less than 1.00 |
| ENSG00000157600.10 | protein_coding | *TMEM164* | 2.52 | 3.58 | 0.87 | 87.00 | less than 3.24 |
| ENSG00000112562.17 | protein_coding | *SMOC2* | 2.60 | 5.18 | 0.87 | 87.00 | less than 4.08 |
| ENSG00000154928.15 | protein_coding | *EPHB1* | 1.00 | 2.55 | 0.87 | 92.15 | less than 2.08 |
| ENSG00000166482.10 | protein_coding | *MFAP4* | 2.36 | 5.08 | 0.87 | 85.20 | less than 4.03 |
| ENSG00000235978.5 | antisense | *AC018816.3* | 1.68 | 0.37 | 0.87 | 78.25 | more than 1.13 |
| ENSG00000128283.6 | protein_coding | *CDC42EP1* | 4.88 | 3.73 | 0.87 | 76.46 | more than 4.44 |
| ENSG00000165795.19 | protein_coding | *NDRG2* | 2.97 | 3.82 | 0.87 | 84.98 | less than 3.54 |
| ENSG00000140092.13 | protein_coding | *FBLN5* | 2.50 | 3.80 | 0.87 | 84.98 | less than 3.27 |
| ENSG00000118971.6 | protein_coding | *CCND2* | 5.58 | 4.42 | 0.87 | 84.98 | more than 4.98 |
| ENSG00000166426.7 | protein_coding | *CRABP1* | 3.31 | 6.79 | 0.87 | 78.03 | less than 5.00 |
| ENSG00000155849.14 | protein_coding | *ELMO1* | 2.24 | 4.23 | 0.87 | 83.18 | less than 3.45 |
| ENSG00000244968.5 | antisense | *LIFR-AS1* | 0.68 | 1.48 | 0.87 | 83.18 | less than 1.01 |
| ENSG00000163681.13 | protein_coding | *SLMAP* | 2.03 | 2.80 | 0.87 | 88.34 | less than 2.53 |
| ENSG00000108846.14 | protein_coding | *ABCC3* | 3.14 | 1.33 | 0.87 | 86.55 | more than 2.04 |
| ENSG00000259104.2 | lincRNA | *PTCSC3* | 4.77 | 6.40 | 0.87 | 86.55 | less than 6.00 |
| ENSG00000136895.17 | protein_coding | *GARNL3* | 0.66 | 1.30 | 0.87 | 91.70 | less than 1.08 |
| ENSG00000164176.11 | protein_coding | *EDIL3* | 1.94 | 0.85 | 0.87 | 84.75 | more than 1.32 |
| ENSG00000265666.1 | antisense | *RARA-AS1* | 2.07 | 1.18 | 0.87 | 84.75 | more than 1.59 |
| ENSG00000198088.9 | protein_coding | *NUP62CL* | 1.48 | 2.31 | 0.87 | 89.91 | less than 2.07 |
| ENSG00000101412.12 | protein_coding | *E2F1* | 2.17 | 0.90 | 0.87 | 89.91 | more than 1.28 |
| ENSG00000130827.6 | protein_coding | *PLXNA3* | 2.73 | 1.99 | 0.87 | 77.80 | more than 2.50 |
| ENSG00000272482.1 | lincRNA | *RP11-474O21.5* | 3.85 | 1.16 | 0.87 | 76.01 | more than 2.18 |
| ENSG00000256969.1 | lincRNA | *RP11-320N7.2* | 1.55 | 0.33 | 0.87 | 76.01 | more than 1.00 |
| ENSG00000107404.16 | protein_coding | *DVL1* | 4.97 | 4.13 | 0.87 | 81.17 | more than 4.46 |
| ENSG00000105641.3 | protein_coding | *SLC5A5* | 0.70 | 3.57 | 0.87 | 81.17 | less than 1.00 |
| ENSG00000085741.11 | protein_coding | *WNT11* | 0.64 | 1.90 | 0.87 | 81.17 | less than 1.01 |
| ENSG00000094963.12 | protein_coding | *FMO2* | 0.50 | 1.57 | 0.87 | 86.32 | less than 1.00 |
| ENSG00000173805.14 | protein_coding | *HAP1* | 0.36 | 1.27 | 0.87 | 91.48 | less than 1.01 |
| ENSG00000166415.13 | protein_coding | *WDR72* | 2.24 | 4.41 | 0.87 | 79.37 | less than 3.03 |
| ENSG00000260992.1 | antisense | *DOCK9-AS2* | 3.68 | 2.27 | 0.87 | 84.53 | more than 2.78 |
| ENSG00000182199.9 | protein_coding | *SHMT2* | 3.59 | 2.95 | 0.87 | 94.84 | more than 2.94 |
| ENSG00000163827.11 | protein_coding | *LRRC2* | 2.47 | 3.72 | 0.87 | 77.58 | less than 3.08 |
| ENSG00000162069.13 | protein_coding | *CCDC64B* | 2.37 | 1.47 | 0.87 | 77.58 | more than 2.11 |
| ENSG00000134042.11 | protein_coding | *MRO* | 0.59 | 2.22 | 0.87 | 77.58 | less than 1.00 |
| ENSG00000177697.16 | protein_coding | *CD151* | 6.36 | 5.31 | 0.86 | 80.94 | more than 5.90 |
| ENSG00000167513.7 | protein_coding | *CDT1* | 1.87 | 0.86 | 0.86 | 86.10 | more than 1.19 |
| ENSG00000115827.12 | protein_coding | *DCAF17* | 1.76 | 2.49 | 0.86 | 91.26 | less than 2.33 |
| ENSG00000166352.14 | protein_coding | *C11orf74* | 3.14 | 4.95 | 0.86 | 79.15 | less than 3.75 |
| ENSG00000091137.10 | protein_coding | *SLC26A4* | 4.14 | 7.12 | 0.86 | 79.15 | less than 6.03 |
| ENSG00000113594.8 | protein_coding | *LIFR* | 1.76 | 3.77 | 0.86 | 89.46 | less than 3.14 |
| ENSG00000205038.10 | protein_coding | *PKHD1L1* | 1.20 | 4.24 | 0.86 | 77.35 | less than 2.00 |
| ENSG00000163347.5 | protein_coding | *CLDN1* | 6.32 | 3.31 | 0.86 | 85.87 | more than 5.15 |
| ENSG00000134013.14 | protein_coding | *LOXL2* | 2.05 | 0.96 | 0.86 | 84.08 | more than 1.41 |
| ENSG00000101463.5 | protein_coding | *SYNDIG1* | 1.12 | 2.49 | 0.86 | 84.08 | less than 2.01 |
| ENSG00000176623.10 | protein_coding | *RMDN1* | 3.09 | 3.85 | 0.86 | 77.13 | less than 3.25 |
| ENSG00000173599.12 | protein_coding | *PC* | 3.44 | 2.28 | 0.86 | 77.13 | more than 2.91 |
| ENSG00000228878.6 | lincRNA | *SEPT7-AS1* | 0.55 | 1.15 | 0.86 | 92.60 | less than 1.00 |
| ENSG00000250899.3 | lincRNA | *RP11-253E3.3* | 0.75 | 1.66 | 0.86 | 80.49 | less than 1.00 |
| ENSG00000011454.15 | protein_coding | *RABGAP1* | 3.43 | 3.97 | 0.86 | 85.65 | less than 3.79 |
| ENSG00000005884.16 | protein_coding | *ITGA3* | 6.18 | 4.85 | 0.86 | 85.65 | more than 5.11 |
| ENSG00000069702.9 | protein_coding | *TGFBR3* | 1.50 | 2.44 | 0.86 | 90.81 | less than 2.17 |
| ENSG00000197894.9 | protein_coding | *ADH5* | 4.33 | 4.91 | 0.86 | 78.70 | less than 4.53 |
| ENSG00000109819.7 | protein_coding | *PPARGC1A* | 1.30 | 3.14 | 0.86 | 78.70 | less than 2.02 |
| ENSG00000109445.9 | protein_coding | *ZNF330* | 3.89 | 4.58 | 0.86 | 78.70 | less than 4.14 |
| ENSG00000168589.13 | protein_coding | *DYNLRB2* | 0.67 | 1.34 | 0.86 | 83.86 | less than 1.02 |
| ENSG00000154655.13 | protein_coding | *L3MBTL4* | 0.80 | 1.55 | 0.86 | 76.91 | less than 1.04 |
| ENSG00000105854.11 | protein_coding | *PON2* | 4.28 | 3.46 | 0.86 | 82.06 | more than 3.81 |
| ENSG00000135547.7 | protein_coding | *HEY2* | 2.89 | 1.42 | 0.86 | 75.11 | more than 2.56 |
| ENSG00000180113.14 | protein_coding | *TDRD6* | 0.49 | 1.26 | 0.86 | 90.58 | less than 1.04 |
| ENSG00000148795.5 | protein_coding | *CYP17A1* | 0.61 | 1.49 | 0.86 | 83.63 | less than 1.03 |
| ENSG00000065154.10 | protein_coding | *OAT* | 4.29 | 4.94 | 0.86 | 83.63 | less than 4.67 |
| ENSG00000250305.7 | protein_coding | *KIAA1456* | 1.88 | 3.30 | 0.86 | 76.68 | less than 2.32 |
| ENSG00000163472.17 | protein_coding | *TMEM79* | 2.67 | 1.36 | 0.86 | 76.68 | more than 1.84 |
| ENSG00000115414.17 | protein_coding | *FN1* | 8.14 | 3.81 | 0.86 | 76.68 | more than 6.12 |
| ENSG00000100253.11 | protein_coding | *MIOX* | 1.55 | 3.17 | 0.86 | 74.89 | less than 2.00 |
| ENSG00000129451.10 | protein_coding | *KLK10* | 3.50 | 0.38 | 0.86 | 80.04 | more than 1.00 |
| ENSG00000122176.10 | protein_coding | *FMOD* | 4.32 | 6.10 | 0.86 | 90.36 | less than 5.76 |
| ENSG00000258472.7 | protein_coding | *RP11-192H23.4* | 0.77 | 1.71 | 0.86 | 83.41 | less than 1.15 |
| ENSG00000143369.13 | protein_coding | *ECM1* | 3.90 | 1.57 | 0.86 | 76.46 | more than 2.46 |
| ENSG00000137709.8 | protein_coding | *POU2F3* | 1.31 | 0.63 | 0.86 | 76.46 | more than 1.02 |
| ENSG00000257267.2 | unitary_pseudogene | *ZNF271P* | 2.78 | 3.25 | 0.86 | 81.61 | less than 3.02 |
| ENSG00000146409.9 | protein_coding | *SLC18B1* | 2.80 | 3.46 | 0.86 | 81.61 | less than 3.14 |
| ENSG00000100373.8 | protein_coding | *UPK3A* | 0.44 | 1.70 | 0.86 | 86.77 | less than 1.00 |
| ENSG00000138735.14 | protein_coding | *PDE5A* | 4.31 | 2.59 | 0.86 | 74.66 | more than 3.74 |
| ENSG00000069974.14 | protein_coding | *RAB27A* | 4.68 | 3.29 | 0.86 | 74.66 | more than 4.49 |
| ENSG00000204934.9 | antisense | *ATP6V0E2-AS1* | 0.81 | 1.90 | 0.86 | 84.98 | less than 1.15 |
| ENSG00000134574.10 | protein_coding | *DDB2* | 2.81 | 1.84 | 0.86 | 90.13 | more than 2.12 |
| ENSG00000186007.8 | protein_coding | *LEMD1* | 1.73 | 0.68 | 0.86 | 78.03 | more than 1.06 |
| ENSG00000163935.12 | protein_coding | *SFMBT1* | 1.28 | 1.91 | 0.86 | 78.03 | less than 1.51 |
| ENSG00000168785.6 | protein_coding | *TSPAN5* | 1.97 | 2.92 | 0.86 | 76.23 | less than 2.38 |
| ENSG00000147041.10 | protein_coding | *SYTL5* | 2.15 | 0.14 | 0.86 | 76.23 | more than 1.00 |
| ENSG00000107731.11 | protein_coding | *UNC5B* | 3.10 | 1.54 | 0.86 | 93.50 | more than 1.76 |
| ENSG00000076382.15 | protein_coding | *SPAG5* | 1.19 | 2.17 | 0.86 | 76.23 | less than 1.38 |
| ENSG00000106809.9 | protein_coding | *OGN* | 0.89 | 2.92 | 0.86 | 86.55 | less than 2.00 |
| ENSG00000223768.1 | lincRNA | *LINC00205* | 2.13 | 2.98 | 0.86 | 79.60 | less than 2.54 |
| ENSG00000164597.12 | protein_coding | *COG5* | 2.33 | 2.83 | 0.86 | 84.75 | less than 2.71 |
| ENSG00000162804.12 | protein_coding | *SNED1* | 1.88 | 2.76 | 0.86 | 84.75 | less than 2.45 |
| ENSG00000143409.14 | protein_coding | *FAM63A* | 3.81 | 4.74 | 0.86 | 84.75 | less than 4.34 |
| ENSG00000132481.5 | protein_coding | *TRIM47* | 3.69 | 2.37 | 0.86 | 77.80 | more than 3.32 |
| ENSG00000104177.16 | protein_coding | *MYEF2* | 1.33 | 0.67 | 0.86 | 77.80 | more than 1.11 |
| ENSG00000086619.12 | protein_coding | *ERO1LB* | 2.37 | 3.49 | 0.86 | 82.96 | less than 3.01 |
| ENSG00000196878.11 | protein_coding | *LAMB3* | 4.43 | 1.34 | 0.86 | 76.01 | more than 3.13 |
| ENSG00000102265.10 | protein_coding | *TIMP1* | 8.16 | 5.63 | 0.86 | 81.17 | more than 7.06 |
| ENSG00000264229.1 | snRNA | *RNU4ATAC* | 0.52 | 2.13 | 0.86 | 86.32 | less than 1.00 |
| ENSG00000110492.14 | protein_coding | *MDK* | 5.91 | 3.76 | 0.86 | 86.32 | more than 4.35 |
| ENSG00000108639.6 | protein_coding | *SYNGR2* | 5.25 | 4.56 | 0.86 | 86.32 | more than 4.84 |
| ENSG00000141738.12 | protein_coding | *GRB7* | 3.32 | 1.75 | 0.86 | 74.22 | more than 2.79 |
| ENSG00000125505.15 | protein_coding | *MBOAT7* | 4.26 | 3.53 | 0.86 | 74.22 | more than 4.05 |
| ENSG00000171552.11 | protein_coding | *BCL2L1* | 5.37 | 4.41 | 0.86 | 79.37 | more than 4.83 |
| ENSG00000164530.12 | protein_coding | *PI16* | 1.20 | 3.80 | 0.86 | 79.37 | less than 2.00 |
| ENSG00000251615.3 | lincRNA | *RP11-774O3.3* | 1.84 | 2.82 | 0.86 | 79.37 | less than 2.35 |
| ENSG00000161509.12 | protein_coding | *GRIN2C* | 1.17 | 2.96 | 0.86 | 77.58 | less than 2.01 |
| ENSG00000182224.10 | protein_coding | *CYB5D1* | 0.73 | 1.30 | 0.86 | 82.74 | less than 1.00 |
| ENSG00000163191.5 | protein_coding | *S100A11* | 8.76 | 7.25 | 0.86 | 82.74 | more than 7.95 |
| ENSG00000117707.14 | protein_coding | *PROX1* | 0.87 | 2.33 | 0.86 | 93.05 | less than 2.02 |
| ENSG00000100478.13 | protein_coding | *AP4S1* | 1.53 | 1.92 | 0.86 | 86.10 | less than 1.81 |
| ENSG00000167191.10 | protein_coding | *GPRC5B* | 3.87 | 2.84 | 0.86 | 73.99 | more than 3.60 |
| ENSG00000170624.12 | protein_coding | *SGCD* | 1.14 | 2.43 | 0.85 | 84.30 | less than 2.01 |
| ENSG00000105976.13 | protein_coding | *MET* | 5.67 | 3.61 | 0.85 | 84.30 | more than 4.23 |
| ENSG00000160678.10 | protein_coding | *S100A1* | 6.61 | 5.02 | 0.85 | 77.35 | more than 6.09 |
| ENSG00000109610.5 | protein_coding | *SOD3* | 4.11 | 6.33 | 0.85 | 77.35 | less than 5.17 |
| ENSG00000107130.8 | protein_coding | *NCS1* | 3.48 | 4.54 | 0.85 | 82.51 | less than 3.97 |
| ENSG00000067064.9 | protein_coding | *IDI1* | 3.15 | 3.81 | 0.85 | 87.67 | less than 3.57 |
| ENSG00000186153.15 | protein_coding | *WWOX* | 2.21 | 3.21 | 0.85 | 80.72 | less than 2.63 |
| ENSG00000183682.7 | protein_coding | *BMP8A* | 1.27 | 3.90 | 0.85 | 80.72 | less than 2.07 |
| ENSG00000145332.12 | protein_coding | *KLHL8* | 2.33 | 3.08 | 0.85 | 80.72 | less than 2.70 |
| ENSG00000168487.16 | protein_coding | *BMP1* | 3.64 | 2.47 | 0.85 | 73.77 | more than 3.44 |
| ENSG00000115363.12 | protein_coding | *EVA1A* | 2.63 | 1.05 | 0.85 | 73.77 | more than 2.05 |
| ENSG00000124766.5 | protein_coding | *SOX4* | 4.54 | 3.19 | 0.85 | 78.92 | more than 3.76 |
| ENSG00000255020.1 | antisense | *AF131216.5* | 1.05 | 2.66 | 0.85 | 78.92 | less than 2.00 |
| ENSG00000177363.4 | protein_coding | *LRRN4CL* | 0.23 | 1.21 | 0.85 | 94.39 | less than 1.00 |
| ENSG00000011009.9 | protein_coding | *LYPLA2* | 5.32 | 4.51 | 0.85 | 87.44 | more than 4.74 |
| ENSG00000149451.16 | protein_coding | *ADAM33* | 0.42 | 1.22 | 0.85 | 92.60 | less than 1.01 |
| ENSG00000120875.7 | protein_coding | *DUSP4* | 2.73 | 1.13 | 0.85 | 80.49 | more than 2.29 |
| ENSG00000153404.12 | protein_coding | *PLEKHG4B* | 0.52 | 1.67 | 0.85 | 85.65 | less than 1.00 |
| ENSG00000145247.10 | protein_coding | *OCIAD2* | 5.09 | 4.31 | 0.85 | 85.65 | more than 4.57 |
| ENSG00000139684.12 | protein_coding | *ESD* | 4.82 | 5.33 | 0.85 | 78.70 | less than 5.06 |
| ENSG00000159921.13 | protein_coding | *GNE* | 2.08 | 2.60 | 0.85 | 89.01 | less than 2.49 |
| ENSG00000088808.15 | protein_coding | *PPP1R13B* | 3.26 | 3.99 | 0.85 | 89.01 | less than 3.83 |
| ENSG00000274210.1 | snRNA | *U1* | 0.29 | 1.51 | 0.85 | 94.17 | less than 1.00 |
| ENSG00000116194.11 | protein_coding | *ANGPTL1* | 2.36 | 4.33 | 0.85 | 76.91 | less than 3.08 |
| ENSG00000107816.16 | protein_coding | *LZTS2* | 4.45 | 3.70 | 0.85 | 76.91 | more than 4.18 |
| ENSG00000189233.10 | protein_coding | *NUGGC* | 0.53 | 1.81 | 0.85 | 82.06 | less than 1.00 |
| ENSG00000156299.11 | protein_coding | *TIAM1* | 2.41 | 0.75 | 0.85 | 87.22 | more than 1.12 |
| ENSG00000163328.12 | protein_coding | *GPR155* | 1.12 | 1.85 | 0.85 | 75.11 | less than 1.31 |
| ENSG00000150433.8 | protein_coding | *TMEM218* | 1.55 | 2.00 | 0.85 | 75.11 | less than 1.73 |
| ENSG00000149179.12 | protein_coding | *C11orf49* | 3.26 | 2.64 | 0.85 | 75.11 | more than 3.02 |
| ENSG00000183621.14 | protein_coding | *ZNF438* | 2.36 | 2.85 | 0.85 | 80.27 | less than 2.60 |
| ENSG00000166922.7 | protein_coding | *SCG5* | 4.04 | 2.28 | 0.85 | 80.27 | more than 3.14 |
| ENSG00000161896.9 | protein_coding | *IP6K3* | 1.30 | 2.97 | 0.85 | 80.27 | less than 2.01 |
| ENSG00000139318.7 | protein_coding | *DUSP6* | 6.22 | 4.16 | 0.85 | 80.27 | more than 5.39 |
| ENSG00000140945.14 | protein_coding | *CDH13* | 1.45 | 0.54 | 0.85 | 73.32 | more than 1.12 |
| ENSG00000139173.8 | protein_coding | *TMEM117* | 2.00 | 1.25 | 0.85 | 83.63 | more than 1.47 |
| ENSG00000131759.16 | protein_coding | *RARA* | 3.54 | 2.80 | 0.85 | 88.79 | more than 2.99 |
| ENSG00000148948.6 | protein_coding | *LRRC4C* | 0.50 | 1.34 | 0.85 | 87.00 | less than 1.00 |
| ENSG00000164199.14 | protein_coding | *GPR98* | 0.33 | 1.24 | 0.85 | 92.15 | less than 1.01 |
| ENSG00000157765.10 | protein_coding | *SLC34A2* | 7.54 | 3.52 | 0.85 | 74.89 | more than 6.17 |
| ENSG00000143753.11 | protein_coding | *DEGS1* | 5.65 | 5.10 | 0.85 | 74.89 | more than 5.50 |
| ENSG00000085733.14 | protein_coding | *CTTN* | 5.48 | 4.94 | 0.85 | 74.89 | more than 5.31 |
