# Supplementary figures and images for: Expression based biomarkers and models to classify early and late-stage samples of Papillary Thyroid Carcinoma
Source: PLoS One. 2020 Apr 23;15(4):e0231629. doi: 10.1371/journal.pone.0231629 (PMC7179925; doi:10.1371/journal.pone.0231629)

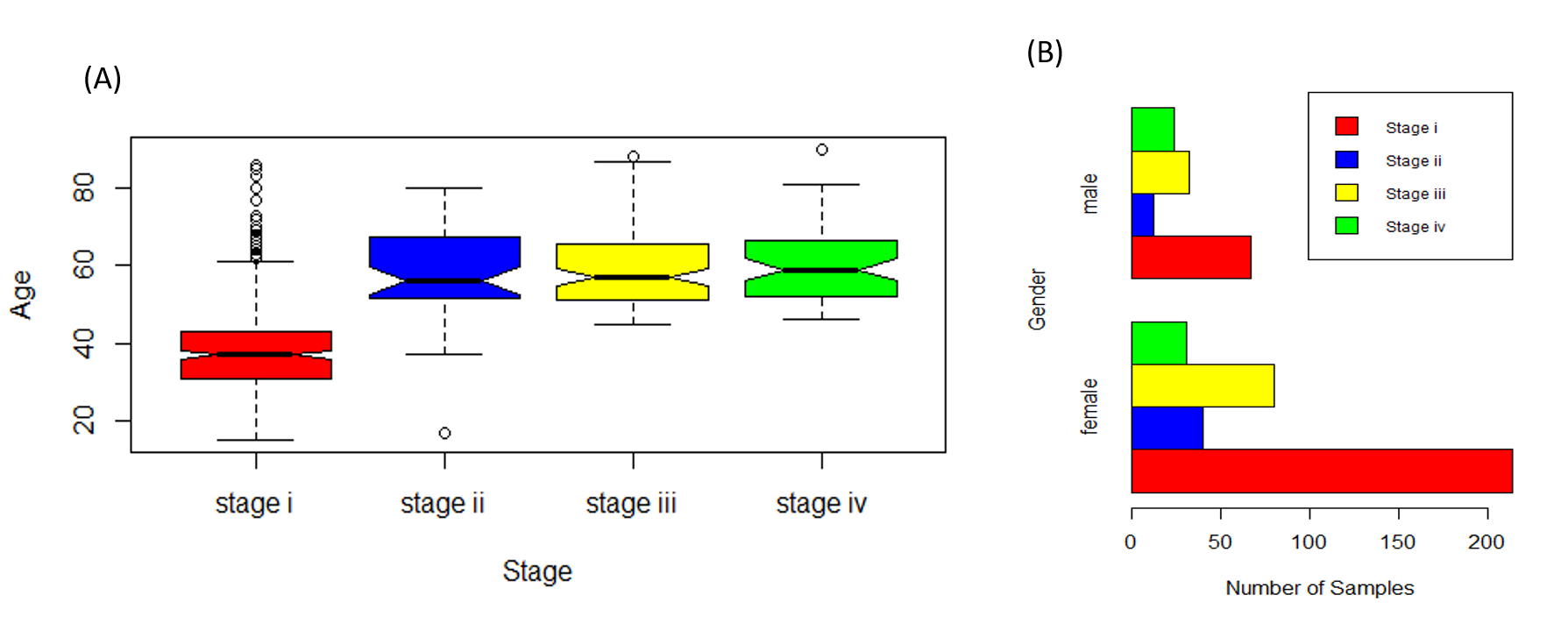

Supplement: S1 Fig — (PNG) [file pone.0231629.s018.png]

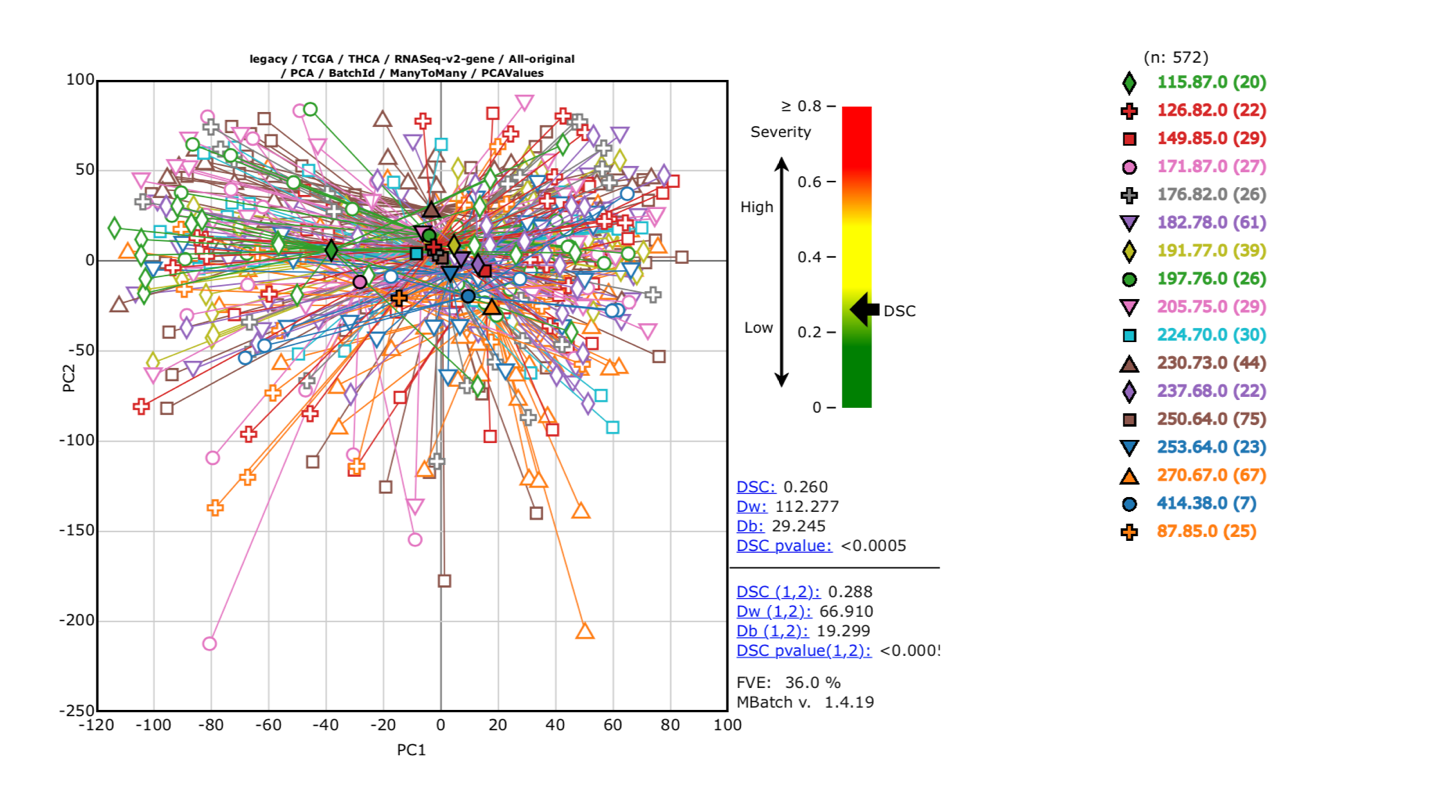

Supplement: S2 Fig — (PNG) [file pone.0231629.s019.png]

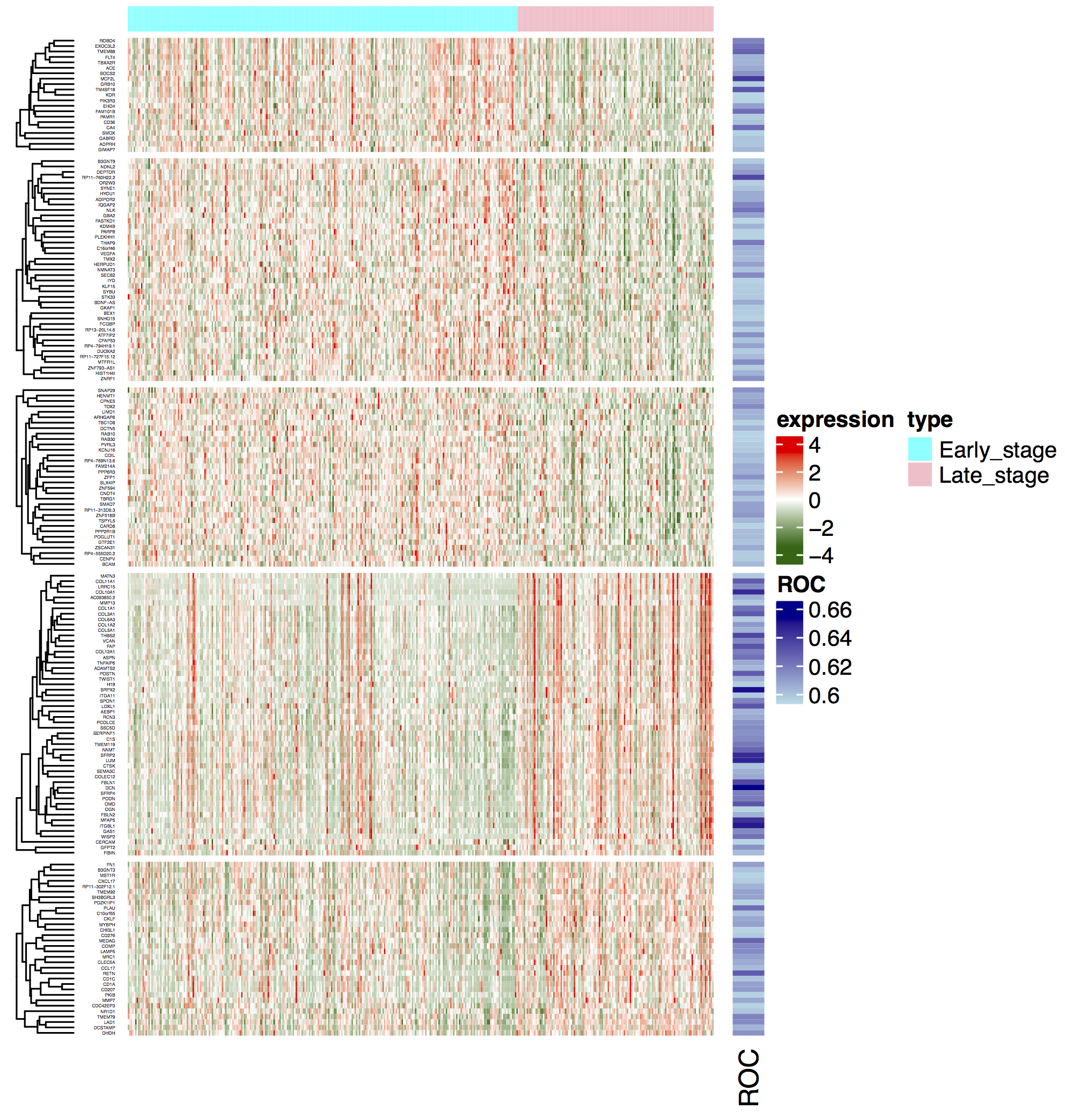

Supplement: S3 Fig — (TIFF) [file pone.0231629.s020.tiff]

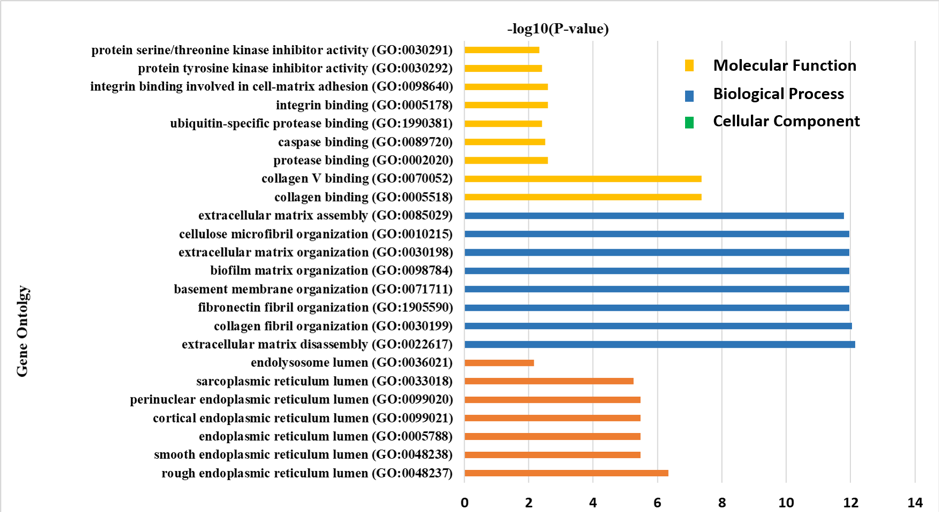

Supplement: S4 Fig — (PNG) [file pone.0231629.s021.png]

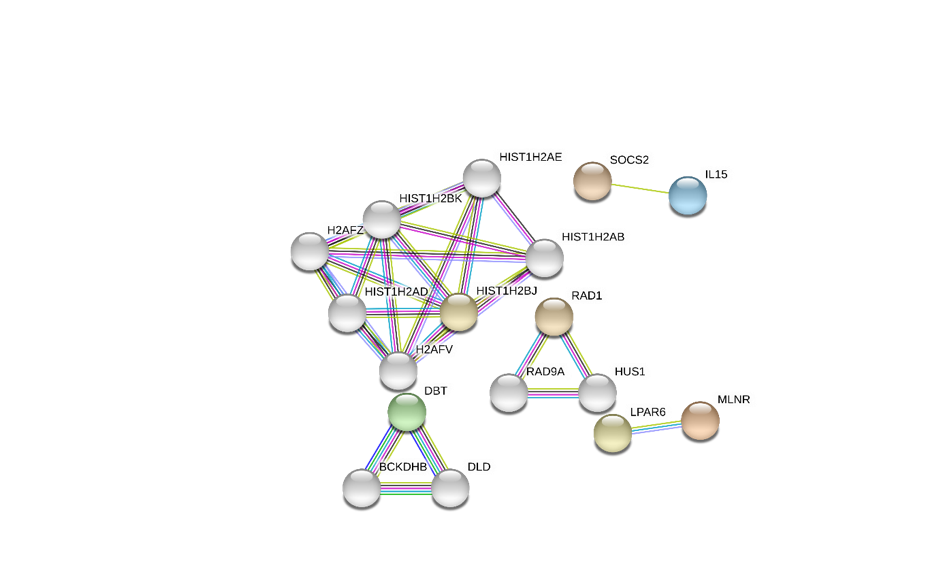

Supplement: S5 Fig — The coloured nodes represent the genes in the signature and white nodes are indirect neighbours added to the network. (PNG) [file pone.0231629.s022.png]

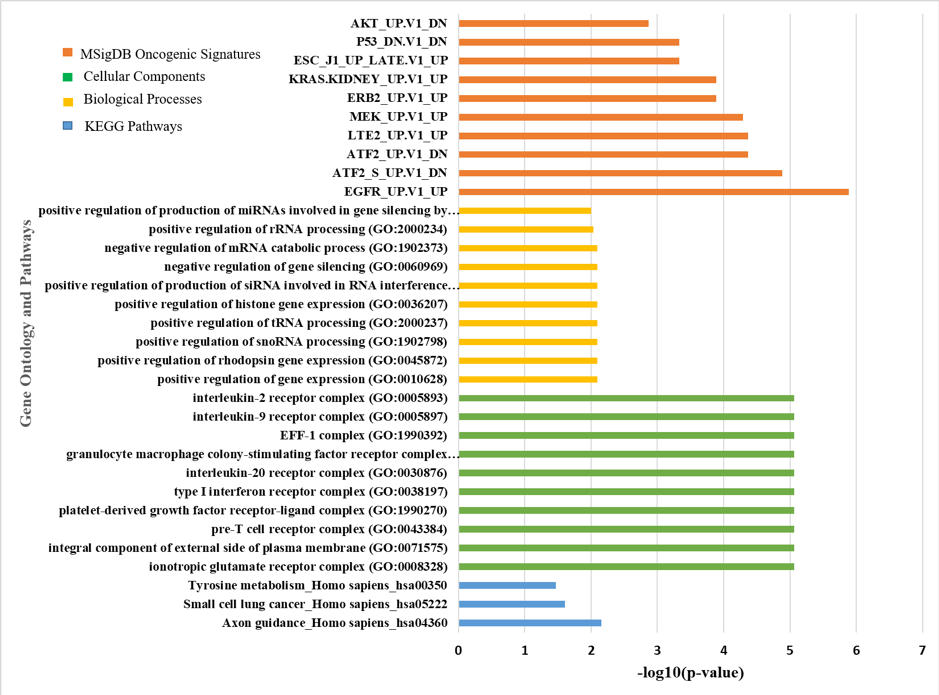

Supplement: S6 Fig — (PNG) [file pone.0231629.s023.png]

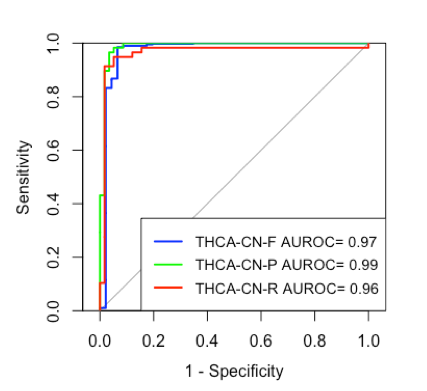

Supplement: S7 Fig — (PNG) [file pone.0231629.s024.png]

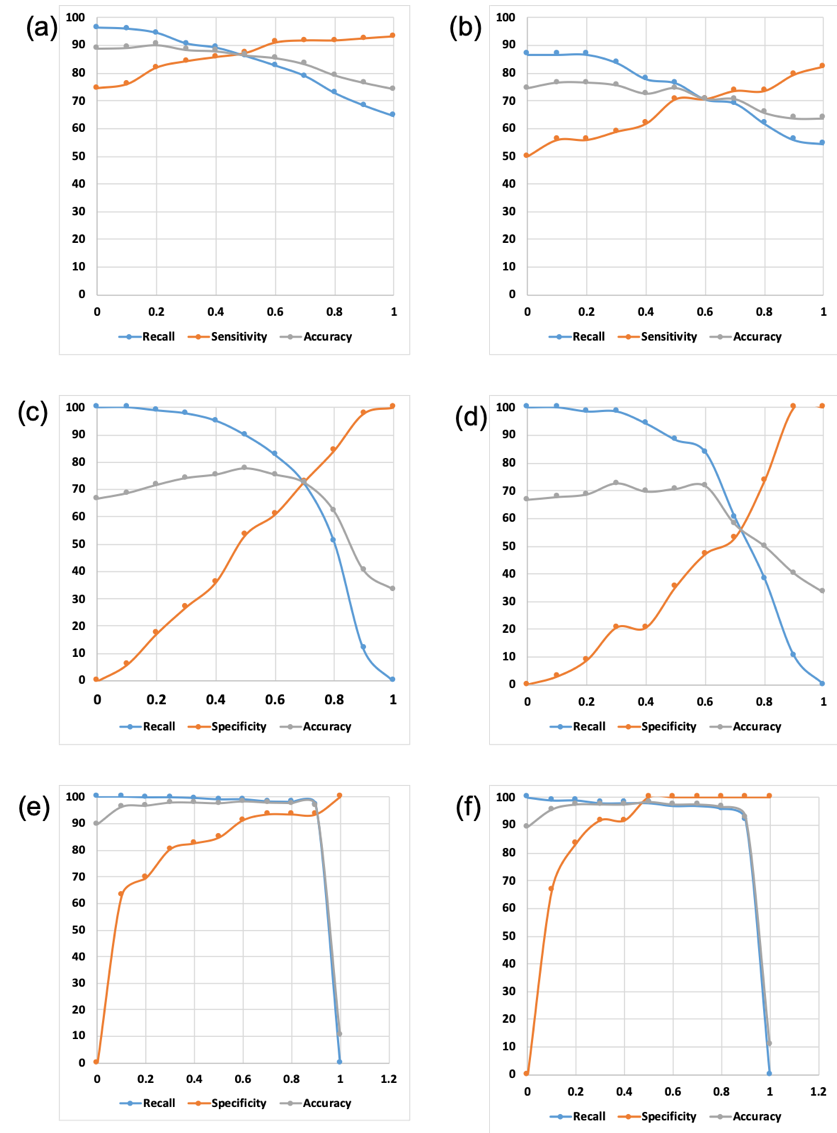

Supplement: S8 Fig — The threshold of the selected models on the webservers (a) all transcript (THCA-EL-SVC-L1) training model (b) all transcript (THCA-EL-SVC-L1) validation model (c) protein coding (THCA-EL-PC) training model (d) protein coding (THCA-EL-PC) validation model (e) Cancer Normal (THCA-CN-F) training model (f) Cancer Normal (THCA-CN-F) validation model. (PNG) [file pone.0231629.s025.png]
